# *N*-Phthalimide as a Site-Protecting and Stereodirecting Group in Rhodium-Catalyzed C–H Functionalization with Donor/Acceptor Carbenes

**DOI:** 10.1021/acs.orglett.3c00844

**Published:** 2023-05-30

**Authors:** Ziyi Chen, Qinyan Cai, Yannick T. Boni, Wenbin Liu, Jiantao Fu, Huw M. L. Davies

**Affiliations:** Department of Chemistry, Emory University, 1515 Dickey Drive, Atlanta, Georgia 30322, United States

## Abstract

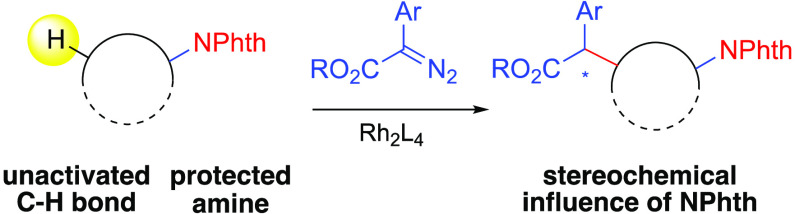

The rhodium-catalyzed enantioselective C–H functionalization of unactivated C–H bonds by means of donor/acceptor carbene-induced C–H insertion was extended to substrates containing nitrogen functionality. The rhodium-stabilized donor/acceptor carbenes were generated by rhodium-catalyzed decomposition of aryldiazoacetates. The phthalimido group was the optimum nitrogen protecting group. C–H functionalization at the most sterically accessible methylene site was achieved using Rh_2_(*S*-2-Cl-5-BrTPCP)_4_ as catalyst, whereas Rh_2_(*S*-TPPTTL)_4_ was the most effective catalyst for C–H functionalization at tertiary C–H bonds and for the desymmetrization of *N*-phthalimidocyclohexane.

The design of new approaches for catalyst-controlled C–H functionalization is a research area of intense current interest.^1^ One particularly useful approach is the C–H functionalization by means of metal carbene-induced C–H insertion.^2,3^ For some time, we have been examining the rhodium-catalyzed enantioselective C–H functionalization chemistry of donor/acceptor carbenes (Scheme 1).^4−6^ They are a privileged class of carbenes because the acceptor group makes the carbene very electrophilic and sufficiently reactive to functionalize unactivated C–H bonds, but the donor group modulates this reactivity so that the reaction outcome is highly susceptible to catalyst control.^5b^ Dirhodium tetracarboxylates are exceptional catalysts for the C–H functionalization chemistry of these donor/acceptor carbenes. Furthermore, when chiral ligands are suitably designed, they self-assemble during ligand exchange to form high symmetry dirhodium complexes (D_2_, C_4_, or C_2_) capable of inducing high levels of asymmetric induction in the carbene reactions.^5a^ We have now prepared a wide variety of structurally well-defined dirhodium catalysts that can dictate which C–H bond in a substrate will be functionalized.^5a^ The original work focused on C–H functionalization of activated C–H bonds such as allylic or benzylic sites, or sites adjacent to oxygen or nitrogen.^5c^ Our more recent work has focused on reactions at unactivated C–H bonds, and catalysts have been designed to distinguish between functionalizing the most accessible, primary, secondary, or tertiary C–H bonds.^7^

**Scheme 1:**
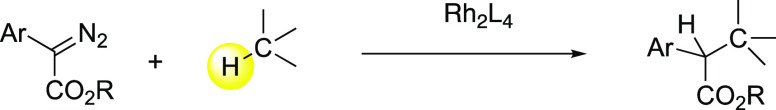
Carbene-Induced C–H Functionalization

One of the most distinctive features of the rhodium-catalyzed C–H functionalization with donor/acceptor carbenes is that the reaction can be conducted in the presence of a variety of functional groups.^5a,5c^ This is especially so when the functionalization is conducted at activated C–H bonds, but even when the reaction is occurring at unactivated C–H bonds, a variety of functionality, such as esters, siloxy, halide, and *p*-substituted aryl can be accommodated into the substrate. Demonstration that the reaction on unactivated C–H bonds can be extended to nitrogen functionality would greatly increase the versatility of the chemistry (Scheme 2). However, amine functionality would offer a number of competing reaction pathways such as ylide formation, insertion into N–H bonds or into the activated C–H bonds adjacent to nitrogen.^8−10^ Furthermore, the amine could poison the catalyst through competing coordination to the axial sites on the dirhodium. We have demonstrated that C–H functionalization at the activated site α to nitrogen can be conducted in the presence of amino functionality suitably protected as the carbamate,^8^ disilazides,^9^ or *N*,*N*-dialkylanilines.^10^ In this manuscript, we describe a successful strategy for C–H functionalization of unactivated sites distal to *N*-phthalimido-protected primary amines.

**Scheme 2:**
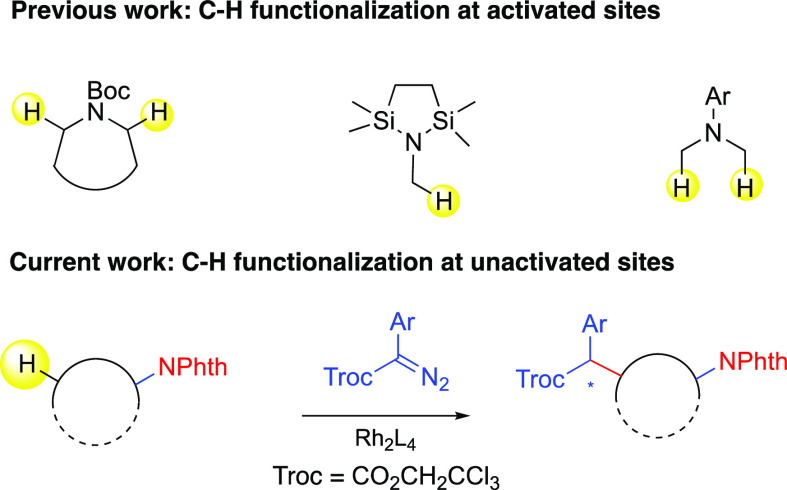
C–H Functionalization in the Presence of Amino Functionality

The study was conducted with two of our more established *C*_4_ symmetric catalysts, Rh_2_(*S*-2-Cl-5-BrTPCP)_4_^11^ and Rh_2_(*S*-TPPTTL)_4_^7b^ (Figure 1). Rh_2_(*S*-2-Cl-5-BrTPCP)_4_ is a sterically demanding catalyst that causes the C–H functionalization to occur at the most accessible methylene site.^11^ Rh_2_(*S*-TPPTTL)_4_ is not as sterically crowded and can cause C–H functionalization to occur at a tertiary site and can even differentiate between relatively similar secondary sites due to the bowl shape of the catalyst.^7b^

**Figure 1:**
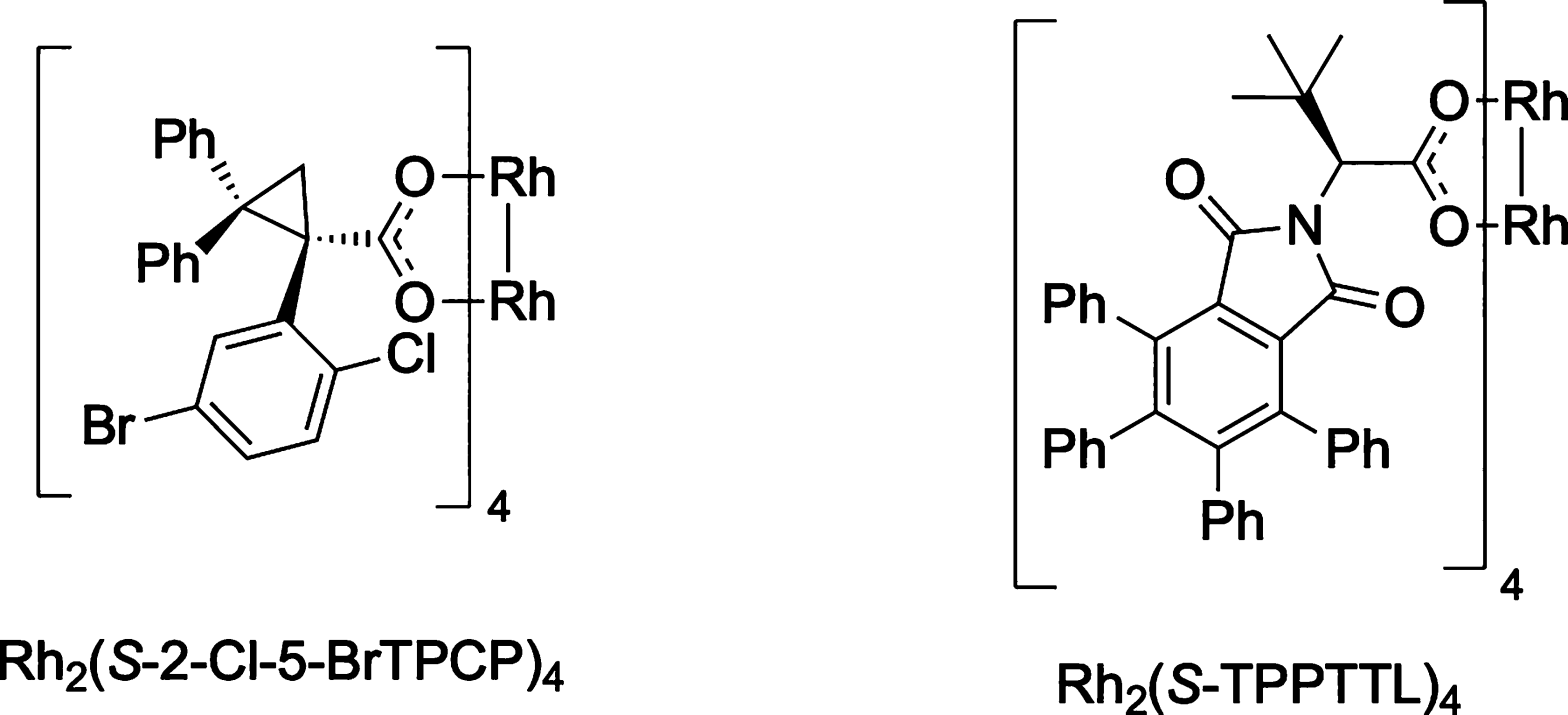
Chiral dirhodium catalysts used in this study.

Initial studies began by determining which nitrogen protecting group would be most suitable, using *N*-protected 1-hexylamines **1** as test substrates (Scheme 3). Rh_2_(*S*-2-Cl-5-BrTPCP)_4_ was utilized for C–H functionalization of the unactivated C–H bonds with the *p*-bromophenyldiazoacetate **2**. The trichloroethyl derivative of **2** was used because it has been shown to give higher yields and levels of enantioselectivity than the methyl ester in functionalization of unactivated C–H bonds.^7^ Neither the Boc protected amine **1a** nor the acetamide **1b** formed the desired C–H functionalization product. In the case of **1a**, N–H insertion was observed, whereas in the case of **1b**, a complex mixture was generated, suggesting that the amide functionality was not inert under these conditions. On the expectation that a more sterically crowded amide would be more compatible with this chemistry, the pivalamide **1c** was examined and in this case the C–H functionalization product **3c** was formed in 24% yield. The most effective system, however, was the phthalimido group, and the reaction of **1d** generated the desired C2 substituted product **3d** in 79% yield. Additionally, **3d** was formed with very high levels of site selectivity (>20:1 r.r.), diastereoselectivity (21:1 d.r.), and enantioselectivity (95:5 e.r.). No reaction occurs at the site adjacent to the nitrogen, presumably because of the steric size and the strong electron-withdrawing character of the phthalimido group.

**Scheme 3:**
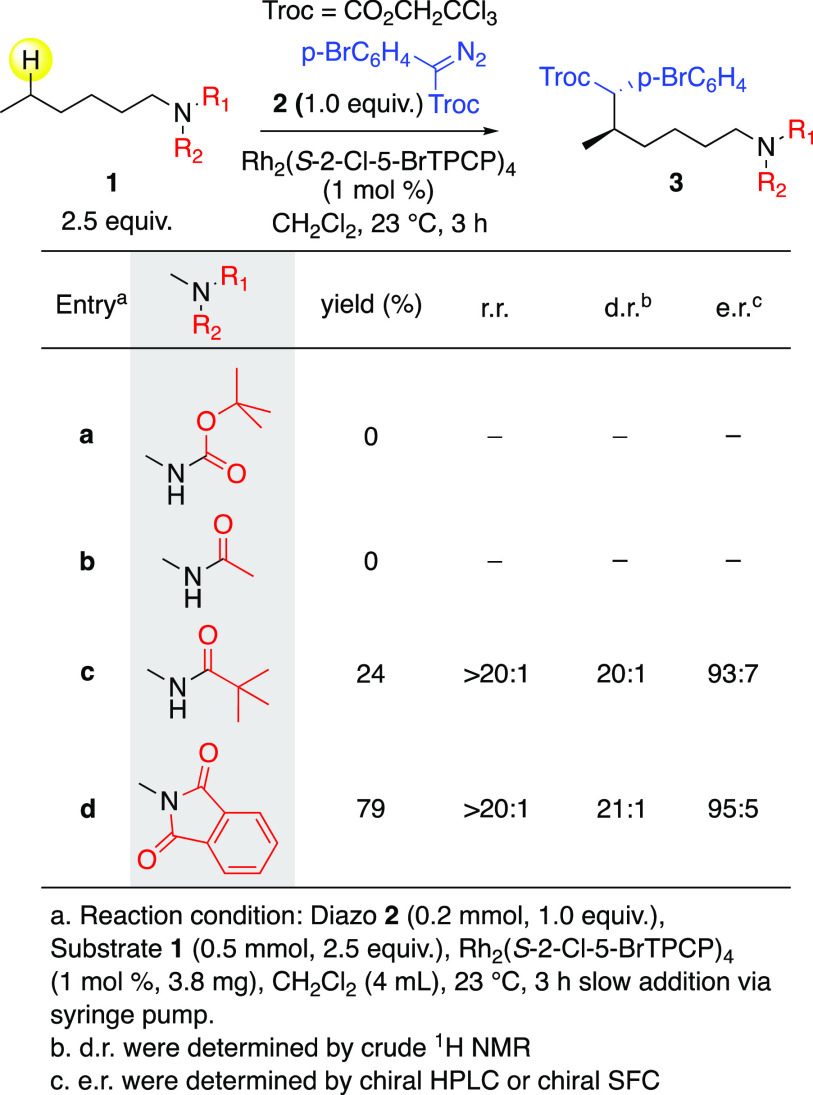
Influence of Amine Protecting Group

The phthalimido group would be expected to be inductively electron-withdrawing, and this inductive effect should protect C–H bonds relatively near to the phthalimido group.^5c^ In order to evaluate the extent of the inductive effect, shorter alkylamine derivatives were examined (Scheme 4). The reaction with pentylamine derivative **4a** was still effective, resulting in the formation of **5a** in 68% yield. Furthermore, the site selectivity remained high, favoring the distal methylene site over the distal methyl site (>20:1 r.r.). In contrast the reaction with the butylamine derivative **4b** resulted in the formation of only traces of **5b** (<10% by NMR). These results indicate that the inductive effect of the phthalimido group still influences the C–H functionalization at sites three carbons away from the group. This effect could be useful in certain cases, because the phthalimido group could protect many relatively close C–H bonds from being prone to C–H functionalization.

**Scheme 4:**
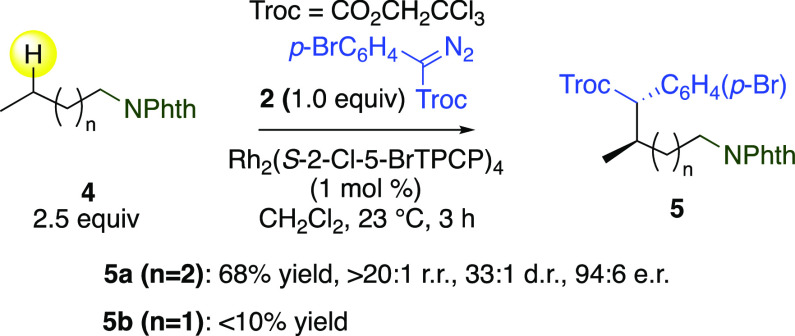
Inductive Effect of NPhth Group

In order to illustrate the potential scope of these reactions, some examples illustrating C–H functionalization at 3° sites were examined. Rh_2_(*S*-2-Cl-5-BrTPCP)_4_ is too sterically demanding for carbene reactions at 3° C–H bonds, and so the less crowded catalyst, Rh_2_(*S*-TPPTTL)_4_, was used. The reaction with phthalimido adamantane **6** proceeded smoothly, generating the tertiary C–H functionalization product **7** in 56% yield and 95:5 e.r. (Scheme 5).

**Scheme 5:**
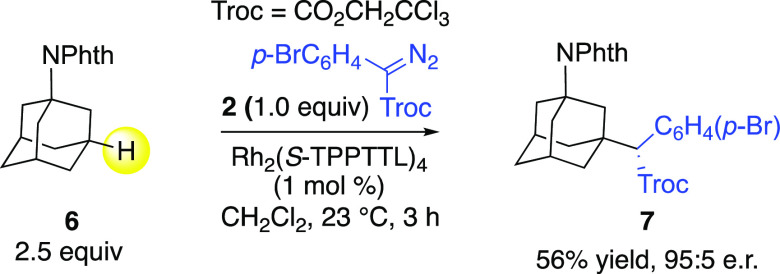
Adamantane C–H Functionalization

The reaction with the 4-methylcyclohexylamine derivative **8** illustrates the subtle control exhibited in this C–H functionalization chemistry (Scheme 6). The commercially available substrate consists of a mixture of trans and cis isomers (**8a** and **8b**). Even so, the reaction resulted in the formation of **9** as a single diastereomer, in which only the cis isomer **8b** had reacted. The relative and absolute stereochemical configuration of **9** was determined by X-ray crystallography. Previously, it has been shown that donor/acceptor carbenes have a strong preference for reaction at equatorial C–H bonds in cyclohexanes (estimated as about 140:1 in favor of equatorial versus axial).^7b^ In the cis isomer **8b** the preferred conformer would have the C4 hydrogen in an equatorial position, but the trans isomer **8a** would have a considerable amount of the drawn conformer with the large *N*-phthalimido group equatorially positioned and, hence, would have a C–H bond axially positioned at C4. Consequently, the cis isomer **8b** would react in preference to the trans isomer **8a**. Further evidence to support this explanation was seen on measurement of the ratio of the residual trapping agent, which was now enriched in the trans isomer **8a**.

**Scheme 6:**
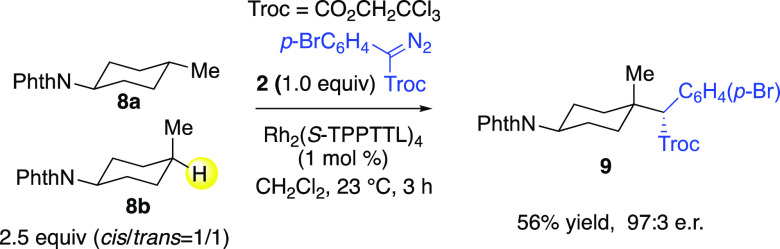
Selective Equatorial C–H Functionalization

All the reactions reported to date have been highly enantioselective. In order to further demonstrate the extent of the chiral influence of the catalyst, the reactions of **2** with the (*S*)-2-amino hexane derivative **10** were examined (Scheme 7). The Rh_2_(*S*-2-Cl-5-BrTPCP)_4_-catalyzed reaction with the aryldiazoacetate **2** gave a preference for one of the *anti*-diastereomers **11** (83:13:2:2 d.r.) of the distal methylene functionalized product (see the Supporting Information for the details on the stereochemical assignment). The Rh_2_(*R*-2-Cl-5-BrTPCP)_4_-catalyzed reaction also resulted in an efficient reaction, but the asymmetric induction was less effective, favoring *anti* diastereomer **12** in a 73:21:3:2 d.r., with the other *anti* diastereomer **11** being the next most prevalent. The Rh_2_(*S*-2-Cl-5-BrTPCP)_4_-catalyzed reaction with **10** is the matched reaction, and in this case, the asymmetric induction on formation of the two new stereogenic centers **11** is very high. In both reactions, the two new stereogenic centers in **11** and **12** are formed with a high level of diastereocontrol in relationship to each other, favoring the *anti*-diastereomers.

**Scheme 7:**
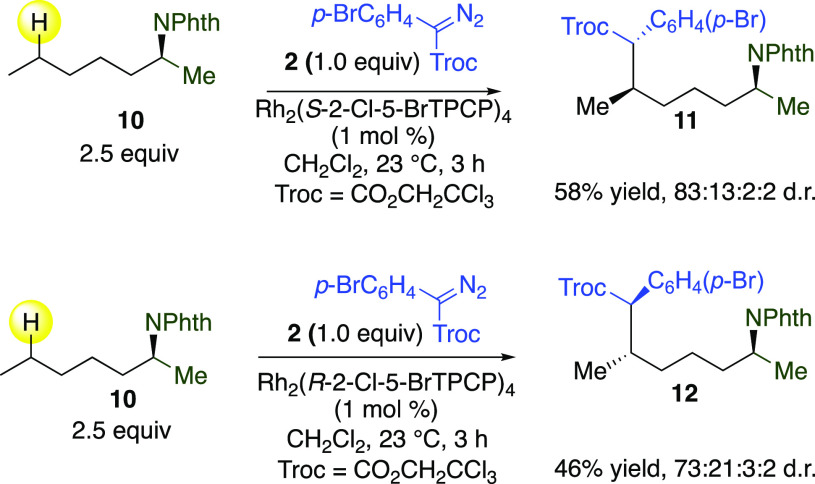
C–H Functionalization of a Chiral Amine Derivative

One of the most impressive examples of site-selective C–H functionalization with donor/acceptor carbenes is the functionalization of alkylcyclohexanes at C3 under Rh_2_(*S*-TPPTTL)_4_ catalysis.^7b^ Therefore, we decided to explore the reaction of the aminoncyclohexane derivative **13** (Scheme 8). The inductive effect of the *N*-phthalimido group should make the C3 site less favorable, but the interference with the ligands of the bowl-shaped catalyst Rh_2_(*S*-TPPTTL)_4_ when attacking the C4 position is still the dominant influence. The reaction required increased temperature (39 °C) to achieve a 63% yield, but it still proceeded cleanly at C3 versus C4 to form **14** in 17:1 r.r. Furthermore, the reaction is highly enantioselective (97:3 e.r.) and leads to effective desymmetrization (>20:1 d.r., selectivity for C3 over C5).

**Scheme 8:**
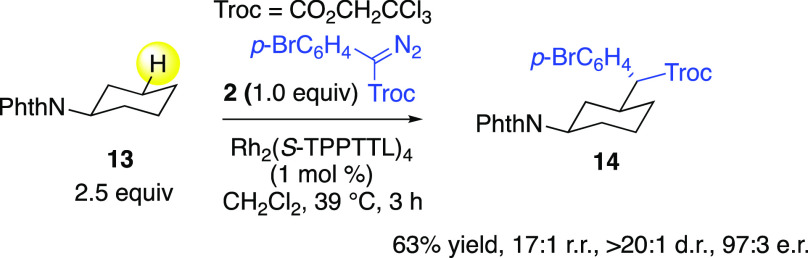
Desymmetrization of Cyclohexane **13**

All the studies to date have been carried out with *p*-bromophenyldiazoacetate as the carbene precursor. However, the chemistry is applicable to a range of aryl and heteroaryldiazoacetate derivatives **15** as illustrated in the Rh_2_(*S*-2-Cl-5-BrTPCP)_4_ reaction of the hexylamine derivative **1d** (Scheme 9). In all instances, C–H functionalization occurs at the distal methylene site to form the C–H functionalization products **16** with high diastereoselectivity (>20:1 d.r.) and with enantioselectivity ranging from 87:13 to 95:5 e.r.

**Scheme 9:**
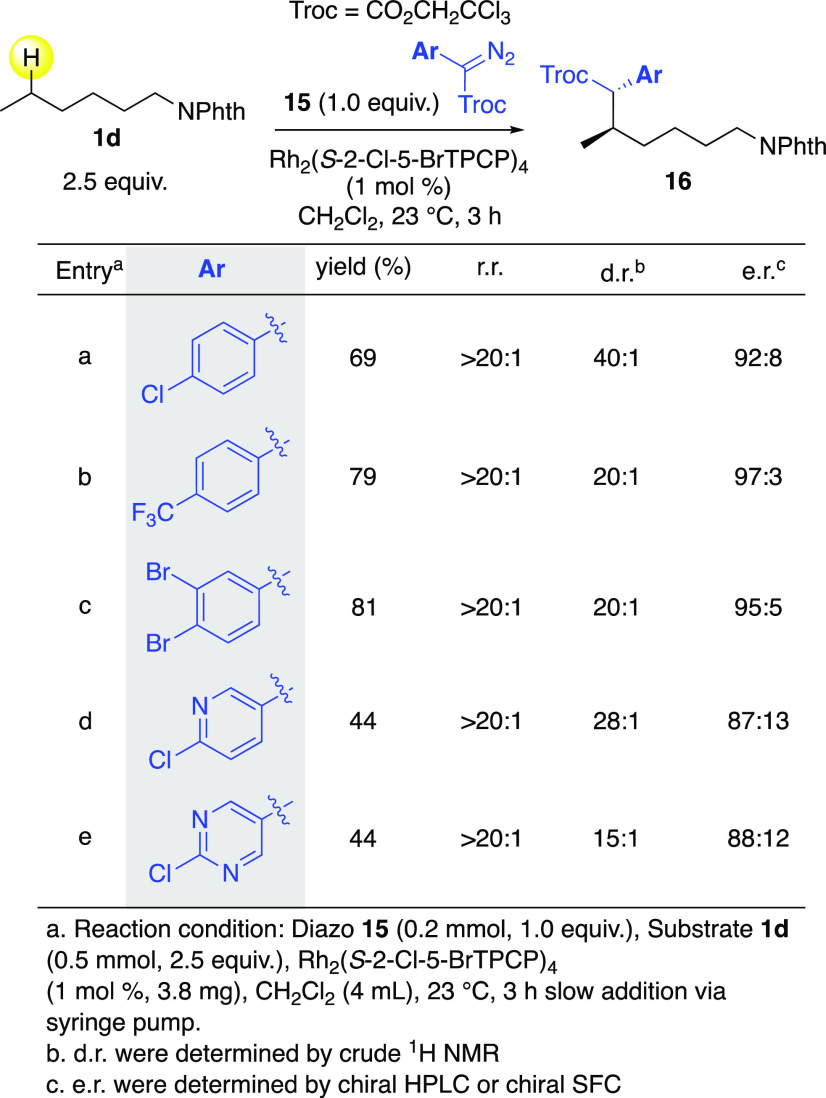
C–H Functionalization with Various Aryl and Heteroaryldiazoacetate Derivatives

In conclusion, The *N*-phthalimido group is effective for protecting primary amines during rhodium-catalyzed functionalization of unactivated C–H bonds with donor/acceptor carbenes. Due to the electron-withdrawing nature of the phthalimido group, it can inductively protect C–H bonds that are close to it. Furthermore, the relatively large nature of the phthalimido group can also protect sites from C–H functionalization and lead to enhanced levels of stereoselectivity. These studies further underscore the functional group compatibility of donor/acceptor carbenes even when they are reacting with unactivated C–H bonds.

## Data Availability

The data underlying this study are available in the published article and its online Supporting Information.

## Supporting Information Available

Complete experimental procedures and compound characterization (PDF)
