# Supplementary material for: N-Phthalimide as a Site-Protecting and Stereodirecting Group in Rhodium-Catalyzed C–H Functionalization with Donor/Acceptor Carbenes
Source: Org Lett. 2023 May 30;25(22):3995–9. doi: 10.1021/acs.orglett.3c00844 (PMC10262272; doi:10.1021/acs.orglett.3c00844)
Supplement: Supplementary file 1 — ol3c00844_si_001.pdf [file ol3c00844_si_001.pdf]

Supplemental Information

N-Phthalimide as a Site-protecting and Stereodirecting Group in Rhodium-Catalyzed C–H Functionalization with Donor/Acceptor Carbenes

Ziyi Chen, Qinyan Cai, Yannick T. Boni, Wenbin Liu, Jiantao Fu and Huw M. L. Davies*

Department of Chemistry, Emory University, 1515 Dickey Drive, Atlanta, GA 30322, US

Table of Contents

1. ***General Information*** 2
2. ***Preparation and Characterization of Substrates*** 4
3. ***Rhodium Catalyzed C–H Functionalization of N-Phthalimido Derivatives and Characterization of Products*** 10
4. ***Reference*** 25
5. ***NMR Spectra*** 26
6. ***Crude NMR for determination of regioselectivity and diastereoselectivity*** 46
7. ***Enantioselectivity Determination by HPLC or SFC*** 59
8. ***X-Ray Crystallographic Data for Compound 9*** 70

1. General Information

All reagents and solvents were used as purchase from commercial sources (Sigma) for substrate synthesis unless otherwise noted. Dichloromethane used in C–H insertion reactions was prepared from solvent purification system. 4 Å molecular sieves was activated at 220 °C for 4 hours under vacuum and stored in an oven over 100 °C. All column chromatography was performed on silica gel (SiliaFlash® P60, 40-63 μm). Thin layer chromatographic (TLC) analysis was performed with aluminum-sheet silica gel plates.

^{1}H, ^{13}C and ^{19}F NMR spectra were recorded at 600 MHz on Bruker-600 spectrometer or Varian IVONA-600 spectrometer (^{13}C at 151 MHz), 500 MHz on Varian INOVA-500 spectrometer, or 400 MHz (^{13}C at 101 MHz, ^{19}F at 376 MHz) on Bruker-400 spectrometer and all were reported in parts per million (ppm). Unless otherwise noted, ^{1}H, ^{13}C and ^{19}F NMR spectra were performed in solutions of deuterated chloroform ($CDCl_3$) with the residue chloroform set as an internal standard (7.26 ppm for ^{1}H, and 77.16 ppm for ^{13}C). Abbreviations for signal multiplicity are as follow: br = broad, s = singlet, d = doublet, t = triplet, q = quartet, m = multiplet, dd = doublet of doublet, tt = triplet of triplet, qt = quartet of triplet, dtd = doublet of triplet of doublet. Coupling constants (J values) were calculated directly from the spectra.

IR spectra were collected on a Nicolet iS10 FT-IR spectrometer.

Mass spectra were taken on a Thermo Finnigan LTQ-FTMS spectrometer with APCI, ESI or NSI.

Melting points (m.p.) were measured in open capillary tubes with a Mel-Temp Electrothermal melting points apparatus and are uncorrected.

Enantiomeric excess data were obtained from either Agilent 1100 series instrument High Performance Liquid Chromatography (HPLC) or Waters ACQUITY UPC-2 Supercritical Fluid Chromatography (SFC). The HPLC system operated with HPLC grade isopropanol/n-hexane gradient and commercial ChiralPak/ChiralCel columns from Daicel Chemical Industries, notably ChiralPak AD-H (5 μm particle size, 4.6 mm vs. 250 mm), ChiralCel OD-H (5 μm particle size, 4.6 mm vs. 250 mm), ChiralPak AS-H (5 μm particle size, 4.6 mm vs. 250 mm), and Regis (R,R) Whelk-O 1 from Regis Technologies (5 μm particle size, 4.6 mm vs. 250 mm). The SFC system operated with HPLC grade (50% methanol in isopropanol with 0.2% formic acid) and commercial ChiralPak/ChiralCel columns from Daicel Chemical Industries, notably ChiralCel OJ-3 (3 μm particle size, 3.0 mm vs. 150 mm), ChiralCel OZ-3 (3 μm particle size, 3.0 mm vs. 150 mm), Regis (S,S) Whelk-O 1 from Regis Technologies (3.5 μm particle size, 3.0 mm vs. 150 mm), and Trefoil AMY1 from Waters (2.5 μm particle size, 3.0 mm vs. 150 mm).

Chiral HPLC or SFC conditions were determined by obtaining separation of the racemic products using Rh_2(*R/S*-TPPTTL)$_4$[1] as catalyst for C–H functionalization with substrates **6, 8a/8b, 13** and Rh_2(*R/S*-2-Cl-5-BrTPCP)$_4$[2] as catalyst for C–H functionalization with other substrates.

2. Preparation and Characterization of Substrates

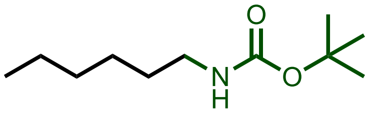

1a

***tert*-Butyl hexylcarbamate (1a)**

1-Hexylamine (10 mmol, 1.01 g, 1.0 equiv) and di-*tert*-butyl dicarbonate (15 mmol, 3.27 g, 1.5 equiv) were dissolved in water (150 mL) and the mixture was stirred for 12 h at room temperature. Chloroform (200 mL) was then added and the mixture was extracted three times with water (3×200 mL). The organic layer was then dried over sodium sulfate and the solvent was removed under reduced pressure. The material was obtained in 60% yield (1.21 g, colorless liquid) and no further purification was needed. The NMR spectroscopic data are in agreement with those reported in the literature[13].

^{1}H NMR (500 MHz, $CDCl_3$) δ 4.49 (s, 1H), 3.10 (q, J = 6.7 Hz, 2H), 1.49–1.44 (m, 11H), 1.34–1.25 (m, 6H), 0.88 (t, J = 6.9 Hz, 3H).

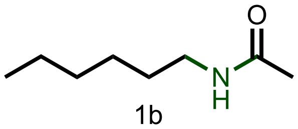

1b

N-hexylacetamide (1b)

1-Hexylamine (10 mmol, 1.01 g, 1.0 equiv) was dissolved in acetonitrile (20 mL) and cooled to 0 °C in an ice water bath. Triethylamine (20 mmol, 2.79 mL, 2.0 equiv) and acetic anhydride (40 mmol, 3.77 mL, 4.0 equiv) were then added to the solution, which was left stirring overnight at 0 °C. After that, the mixture was concentrated before stirring with deionized water (10 mL) for 15 min. Then, the mixture was extracted with dichloromethane (3×15 mL) and the organic phase was washed with sulfuric acid (1 M, 1×15 mL), saturated aqueous solution of sodium bicarbonate (1×15 mL) and water (1×15 mL). The organic layer was dried over anhydrous sodium sulfate, concentrated to afford the product in 52% yield (745 mg, colorless liquid) without further purification. The NMR spectroscopic data are in agreement with those reported in the literature[11].

^{1}H NMR (500 MHz, $CDCl_3$) δ 5.89 (br s, 1H), 3.23 (td, J = 7.3, 5.5 Hz, 2H), 1.97 (s, 3H), 1.51 – 1.45 (m, 2H), 1.34 – 1.25 (m, 6H), 0.87 (t, J = 7.1 Hz, 3H).

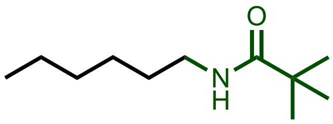

1c

N-hexylpivalamide (1c)

1-Hexylamine (10 mmol, 1.01 g, 1.0 equiv) was dissolved in acetonitrile (20 mL) and cooled to 0 °C in an ice water bath. Triethylamine (20 mmol, 2.79 mL, 2.0 equiv) and pivalic anhydride (40 mmol, 8.12 mL, 4.0 equiv) were then added to the solution, which was left stirring overnight at 0 °C. The mixture was concentrated before stirring with water (10 mL) for 15 min. Then, the solution was extracted with dichloromethane (3×15 mL) and the organic phase was washed with sulfuric acid (1 M, 1×15 mL), saturated aqueous solution of sodium bicarbonate (1×15 mL) and deionized water (1×15 mL), which was then dried over anhydrous sodium sulfate, concentrated to afford the product in 61% yield (1.13 g, colorless liquid) without further purification. The NMR spectroscopic data are in agreement with those reported in the literature[12].

^{1}H NMR (500 MHz, $CDCl_3$) δ 5.61 (br s, 1H), 3.22 (td, *J* = 7.2, 5.6 Hz, 2H), 1.52 – 1.44 (m, 2H), 1.33 – 1.28 (m, 6H), 1.19 (s, 9H), 0.87 (t, *J* = 7.2 Hz, 3H).

General Procedures for Formation of Phthalimides

General Procedure A

Phthalic anhydride (10 mmol, 1.0 equiv) and the desired amine (10 mmol, 1.0 equiv) were added to a round-bottom flask (50 mL) equipped with a large egg-shaped magnetic stir-bar and a condenser. *N,N*-Dimethylformamide (20 mL) was added and the stirred mixture was heated at 120 °C in an oil bath for 12 h. The hot reaction mixture was then poured onto crushed ice prepared in a beaker (150 mL). The resulting precipitate was vacuum filtered, rinsed with deionized water three times and then dried under reduced pressure. The obtained crude product was further purified by silica gel chromatography.

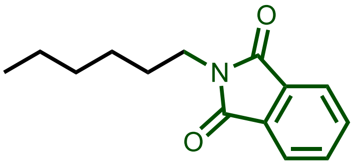

1d

2-Hexylisoindoline-1,3-dione (1d)

The product was obtained in 73% yield (1.69 g, white solid) by the general procedure A with 1-hexylamine (10 mmol, 1.01 g). The product was sufficiently pure that no silica gel chromatographic purification was needed. The spectroscopic data are in agreement with those reported in the literature[7].

^{1}H NMR (500 MHz, $CDCl_3$) δ 7.86 – 7.82 (m, 2H), 7.72 – 7.68 (m, 2H), 3.67 (t, J = 7.4 Hz, 2H), 1.71 – 1.62 (m, 2H), 1.38 – 1.26 (m, 6H), 0.87 (t, J = 7.4 Hz, 3H).

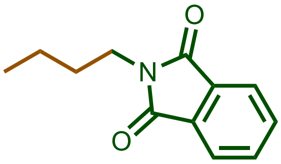

4a

2-Butylisoindoline-1,3-dione (4a)

The product was obtained by the general procedure A in 70% yield (2.84 g, colorless liquid) by the general procedure A with 1-butylamine (20 mmol, 1.98 mL). The silica gel chromatography was run by Biotage system with 20% ethyl acetate/hexane as solvent. The spectroscopic data are in agreement with those reported in the literature[5].

^{1}H NMR (400 MHz, $CDCl_3$) δ 7.85 – 7.78 (m, 2H), 7.72 – 7.65 (m, 2H), 3.66 (t, J = 7.2 Hz, 2H), 1.64 (tt, J = 7.6, 6.3 Hz, 2H), 1.35 (dq, J = 14.9, 7.4 Hz, 2H), 0.92 (t, J = 7.4 Hz, 3H).

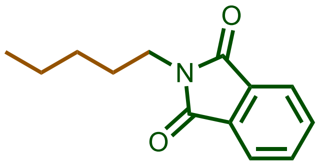

4b

2-Pentylisoindoline-1,3-dione (4b)

The product was obtained by the general procedure A in 72% yield (1.56 g, colorless liquid) by the general procedure A with 1-pentylamine (10 mmol, 2.30 mL). The silica gel chromatography was run by Biotage system with 10%-15% diethyl ether/hexane as solvent gradient. The spectroscopic data are in agreement with those reported in the literature[6].

^{1}H NMR (500 MHz, $CDCl_3$) δ 7.86 – 7.81 (m, 2H), 7.74 – 7.66 (m, 2H), 3.67 (t, J = 7.4 Hz, 2H), 1.67 (p, J = 7.4 Hz, 2H), 1.40 – 1.26 (m, 4H), 0.89 (t, J = 7.0 Hz, 3H).

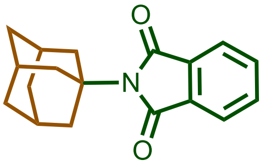

6

2-(Adamantan-1-yl)isoindoline-1,3-dione (6)

The product was obtained by general procedure A in 64% (1.79 g, white solid) yield by the general procedure A with adamantan-1-amine (9.92 mmol, 1.47 g). The silica gel chromatography was run by Biotage with 10% ethyl acetate/hexane. Spectroscopic data are in agreement with those reported in the literature[9].

^{1}H NMR (400 MHz, $CDCl_3$) δ 7.74 (td, J = 5.2, 2.0 Hz, 2H), 7.67 (td, J = 5.2, 2.0 Hz, 2H), 2.51 (d, J = 2.8 Hz, 6H), 2.16 (dd, J = 4.9, 2.1 Hz, 3H), 1.79 (dt, J = 12.5, 2.1 Hz, 3H), 1.71 (dt, J = 12.5, 3.2 Hz, 3H).

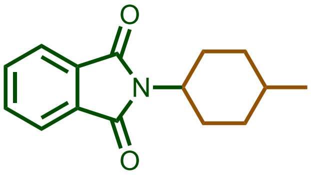

8a/8b = 1/1

2-(4-Methylcyclohexyl)isoindoline-1,3-dione (mixture of 8a/8b = 1/1)

The product was obtained in 55% yield (1.22 g, white solid) by general procedure A with the corresponding 4-methylcyclohexylamine (9.13 mmol, 1.20 mL) purchased from Sigma-Aldrich

(trans/cis = 1/1). The silica gel chromatography was run by Biotage with 8%-10% ethyl acetate/hexane. Spectroscopic data are in agreement with those reported in the literature[10].

^{1}H NMR (400 MHz, $CDCl_3$) δ 7.84 – 7.78 (m, 2H), 7.72 – 7.66 (m, 2H), 4.14 – 4.03 (m, 1H), 2.51 – 2.37 (m, 0.9H), 2.26 (qd, J = 12.8, 3.6 Hz, 1.1H), 1.98 (ddq, J = 10.7, 7.1, 3.5 Hz, 0.5H), 1.86 – 1.77 (m, 1.1H), 1.75 – 1.67 (m, 1.2H), 1.67 – 1.60 (m, 1.8H), 1.48 (ddd, J = 13.0, 6.3, 3.9 Hz, 1.5H), 1.14 – 1.02 (m, 2.5H), 0.92 (d, J = 6.5 Hz, 1.7H). (Mixture of diastereomers)

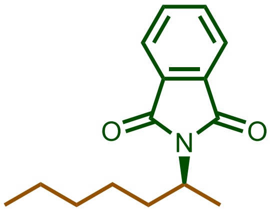

10

(*S*)-2-(Heptan-2-yl)isoindoline-1,3-dione (10)

The product was obtained in 71% yield (1.74 g, colorless liquid) by the general procedure A with (*S*)-heptan-2-amine (10 mmol, 1.50 mL). The silica gel chromatography was run by Biotage with 10% ethyl acetate/hexane. Spectroscopic data are in agreement with those reported in the literature[8].

^{1}H NMR (600 MHz, $CDCl_3$) δ 7.84 – 7.79 (m, 2H), 7.72 – 7.67 (m, 2H), 4.37 – 4.30 (m, 1H), 2.09 – 2.01 (m, 1H), 1.72 (ddt, J = 13.6, 11.3, 5.6 Hz, 1H), 1.46 (d, J = 7.0 Hz, 3H), 1.31 – 1.18 (m, 6H), 0.87 – 0.81 (m, 3H).

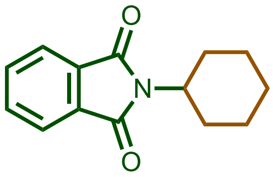

13

2-Cyclohexylisoindoline-1,3-dione (13)

The product was obtained in 83% yield (1.90 g, white solid) by the general procedure A with cyclohexylamine (10 mmol, 1.15 mL). The silica gel chromatography was run by Biotage with 10% ethyl acetate/hexane. Spectroscopic data are in agreement with those reported in the literature[10].

^{1}H NMR (400 MHz, $CDCl_3$) δ 7.81 (tt, J = 5.1, 2.4 Hz, 2H), 7.72 – 7.66 (m, 2H), 4.11 (tt, J = 12.3, 3.9 Hz, 1H), 2.20 (qd, J = 12.6, 3.2 Hz, 2H), 1.86 (dt, J = 12.6, 3.2 Hz, 2H), 1.77 – 1.65 (m, 3H), 1.44 – 1.20 (m, 3H).

Aryldiazoacetate Derivatives

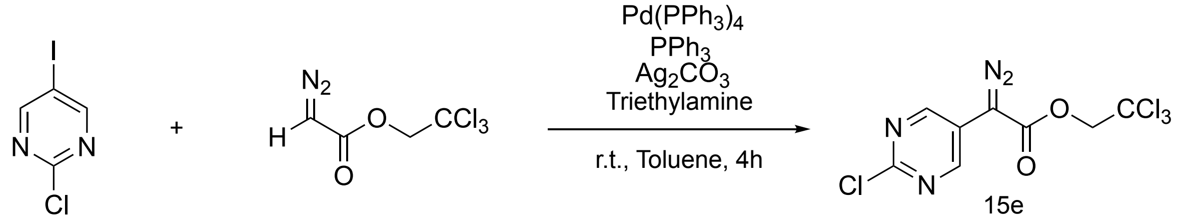

2,2,2-Trichloroethyl 2-(2-chloropyrimidin-5-yl)-2-diazoacetate (15e)

Following the general published procedure[4], a round-bottomed flask equipped with a magnetic stir-bar was evacuated and purged with nitrogen and then tetrakis(triphenylphosphine)palladium, (0.312 mmol, 361 mg, 0.05 equiv) triphenylphosphine (0.624 mmol, 164 mg, 0.1 equiv), 2-chloro-5-iodopyridine (6.24 mmol, 1.50 mg, 1.0 equiv) and silver carbonate (3.12 mmol, 860 mg, 0.5 equiv) were suspended in toluene (30 mL) under nitrogen. Triethylamine (8.74 mmol, 1.22 mL, 1.4 equiv) and 2,2,2-trichloroethyl 2-diazoacetate (6.86 mmol, 1.49 g, 1.1 equiv) was the added and the resulting solution was stirred at room temperature for 4 h and checked by TLC for completion. After the reaction is done, the mixture is passed through a silica plug with 15% diethyl ether/hexane as solvent and the yellow-colored band was collected. The crude material was then purified by silica gel chromatography by Biotage instrument with 10% - 15% - 20% diethyl ether/hexane solvent gradient. The desired product was obtained in 36% yield (741 mg, yellow solid). The spectroscopic data are in agreement with those reported in the literature[4].

^{1}H NMR (500 MHz, $CDCl_3$) δ 8.79 (s, 2H), 4.94 (s, 2H).

3. Rhodium Catalyzed C–H Functionalization of N-Phthalimido Derivatives and Characterization of Products

General Procedure

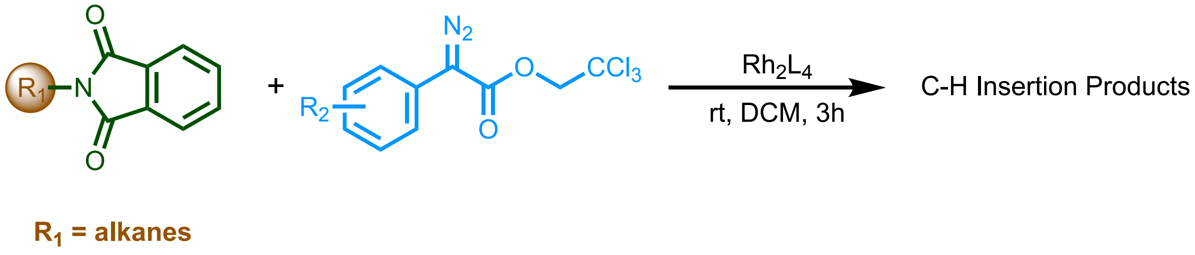

After being flame-dried under vacuum, two separate clean 8.0 mL scintillation vials (vial-A and vial-B) equipped with a small egg-shaped magnetic stir-bar was evacuated and purged with argon/nitrogen (2-3 times). After cooling down to room temperature, the corresponding dirhodium catalyst (1 mol %) followed by substrate (0.5 mmol, 2.5 equiv) were then added in vial-A. The vial-A was once again evacuated and purged with argon (3-5 times) and dry dichloromethane (2 mL) was added. The vial and its content were then set to stir at room temperature under an argon or nitrogen atmosphere. The diazo compound (0.2 mmol, 1 equiv) was added to the other vial (vial-B), which had been evacuated and purged with argon/nitrogen. The vial-B was evacuated and purged with argon (2-3 times) and of dry dichloromethane (2.0 mL) was then added to obtain a 0.1 M solution of the diazo compound. The 0.1 M solution was transferred into a plastic syringe (5 mL, 12.46 mm diameter). Using a well-calibrated syringe pump, the solution of the diazo compound was added slowly (0.667 mL/h) to the stirring reaction mixture in vial-A. After complete addition (3 hours later), the residual diazo compound in the 5.0 mL plastic syringe was rinsed with 0.5 mL dry dichloromethane and transferred dropwise into the stirring reaction mixture of vial-A. The mixture was stirred for an additional 15 min and then concentrated under reduced pressure. The crude product was then purified by silica gel chromatography to obtain the purified C–H functionalization product.

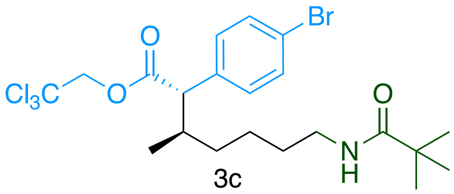

3c

2,2,2-Trichloroethyl (2*S*,3*R*)-2-(4-bromophenyl)-3-methyl-7-pivalamidoheptanoate (3c)

The general procedure was employed for the C–H functionalization of N-hexylpivalamide (92.7 mg, 0.50 mmol) with 2,2,2-trichloroethyl 2-(4-bromophenyl)-2-diazoacetate (74.5 mg, 0.20 mmol) using Rh_2(*S*-2-Cl-5-BrTPCP)$_4$ (3.8 mg, 1 mol %) as catalyst. The crude NMR indicated the presence of the desired product in a 20:1 d.r. plus apparent N-H insertion product and carbene dimers. Flash chromatography on silica gel (gradient 0 to 6 to 8 % ethyl acetate/hexane) afforded **3c** as colorless oil as a major diastereomer (24.4 mg, 24 % yield).

$[\alpha]^{20}_{D}$: +12.0°(c = 0.39, CH_2Cl_2, 93: 7 e.r.); **^{1}H NMR (400 MHz, $CDCl_3$)** major diastereomer δ 7.45 (d, J = 8.4 Hz, 2H), 7.23 (d, J = 8.4 Hz, 2H), 5.54 (br s, 1H), 4.76 (d, J = 12.0 Hz, 1H), 4.62 (d, J = 11.9 Hz, 1H), 3.36 (d, J = 10.7 Hz, 1H), 3.13 (q, J = 6.7 Hz, 2H), 2.29 – 2.17 (m, 1H), 1.44 – 1.25 (m, 4H), 1.16 (s, 10H, tert-butyl group overlapping with one proton from the alkyl chain), 1.04 (d, J = 6.5 Hz, 3H); **^{13}C NMR (151 MHz, $CDCl_3$)** δ 178.4, 171.8, 136.2, 131.9, 130.5, 121.8, 94.9, 74.3, 58.2, 39.5, 38.8, 36.2, 33.1, 29.9, 27.8, 23.8, 17.9; **IR:** (film) 3349, 2930, 2858, 1750, 1637, 1532, 1488, 1463, 1408, 1368, 1276, 1210, 1133, 1074, 1011 cm^{-1}; **HRMS (+p APCI)** calcd for $C_{21}H_{30}O_3N^{79}Br^{35}Cl_3$ $(M+H)^+$: 528.0469; found: 528.0477. The enantiopurity was determined to be 93: 7 e.r. by chiral HPLC analysis (ChiralPak AD-H, 25 cm x 4.6 mm, 1% isopropanol in hexane, 1.50 mL/min, λ = 210 nm, retention times of 47.83 (major) and 52.85 (minor) min). The absolute stereochemistry is tentatively assigned by analogy to the published C2 functionalization with Rh_2(*S*-2-Cl-5-BrTPCP)$_4$[14,15]

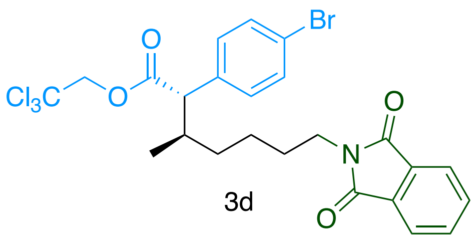

3d

2,2,2-Trichloroethyl (2*S*,3*R*)-2-(4-bromophenyl)-7-(1,3-dioxoisoindolin-2-yl)-3-methylheptanoate (3d)

The general procedure C was employed for the C–H functionalization of 2-hexylisoindoline-1,3-dione (58.0 mg, 0.25 mmol) with 2,2,2-trichloroethyl 2-(4-bromophenyl)-2-diazoacetate (37.3 mg, 0.10 mmol) using Rh_2(*S*-2-Cl-5-BrTPCP)$_4$ (1.9 mg, 1 mol%) as catalyst, which was stirred in 2.0 mL dichloromethane at 23 °C for 3 hours. The crude NMR indicated the presence of the desired product in a 21:1 d.r. Flash chromatography (gradient 0 to 10 to 15% diethyl ether/hexane) afforded the colorless oil as a major diastereomer (45.5 mg, 79 % yield).

$[\alpha]^{20}_D$: +10.5°(c = 0.56, CH_2Cl_2, 95: 5 e.r.); **^{1}H NMR (400 MHz, $CDCl_3$)** major diastereomer δ 7.81 (dd, J = 5.5, 3.0 Hz, 2H), 7.69 (dd, J = 5.5, 3.0 Hz, 2H), 7.41 (d, J = 8.5 Hz, 2H), 7.21 (d, J = 8.5 Hz, 2H), 4.74 (d, J = 12.0 Hz, 1H), 4.61 (d, J = 12.0 Hz, 1H), 3.57 (t, J = 7.2 Hz, 2H), 3.35 (d, J = 10.6 Hz, 1H), 2.29 – 2.16 (m, 1H), 1.62 – 1.51 (m, 1H), 1.51 – 1.42 (m, 1H), 1.44 – 1.28 (m, 1H), 1.23 – 1.13 (m, 2H), 1.03 (d, J = 6.5 Hz, 3H), 1.00 – 0.90 (m, 1H); **^{13}C NMR (101 MHz, $CDCl_3$)** δ 171.8, 168.5, 136.1, 134.0, 132.2, 131.9, 130.5, 123.3, 121.8, 94.8, 74.2, 58.1, 37.9, 36.1, 32.9, 28.7, 23.8, 17.9; **IR:** (film) 2936, 2860, 1749, 1707, 1616, 1488, 1467, 1437, 1396, 1369, 1273, 1188, 1127, 1073, 1011 cm^{-1}; **HRMS (+p APCI)** calcd for $C_{24}H_{24}O_4N^{79}Br^{35}Cl_3$ $(M+H)^+$ 573.9949 found 573.9951. The enantiopurity was determined to be 95: 5 e.r. by chiral HPLC analysis (ChiralPak AS-H, 25 cm x 4.6 mm, 1% isopropanol in hexane, 1.00 mL/min, λ = 210 nm, retention time = 26.56 (major) and 24.15 (minor) min). The absolute stereochemistry is tentatively assigned by analogy to the published C2 functionalization with Rh_2(*S*-2-Cl-5-BrTPCP)$_4$[14,15]

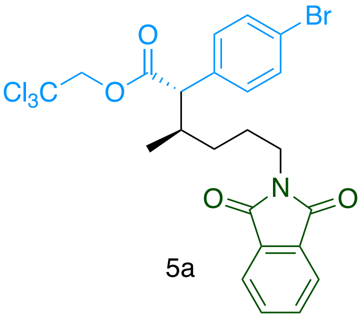

5a

2,2,2-Trichloroethyl (2*S*,3*R*)-2-(4-bromophenyl)-6-(1,3-dioxoisoindolin-2-yl)-3-methylhexanoate (5a)

The general procedure C was employed for the C–H functionalization of 2-pentylisoindoline-1,3-dione (109 mg, 0.50 mmol) with 2,2,2-trichloroethyl 2-(4-bromophenyl)-2-diazoacetate (74.5 mg, 0.20 mmol) using Rh_2(*S*-2-Cl-5-BrTPCP)$_4$ (3.8 mg, 1 mol%) as catalyst, which was stirred in 2.0 mL dichloromethane at 23 °C for 3 hours. The crude NMR indicated the presence of the desired product in a 33:1 d.r. Flash chromatography (gradient 0 to 6 to 12% diethyl ether/hexane) afforded the colorless oil as a major diastereomer (75.3 mg, 68 % yield).

$[\alpha]^{20}_D$: +12.5°(c = 1.83, CH_2Cl_2, 94: 6 e.r.); **^{1}H NMR (500 MHz, $CDCl_3$)** major diastereomer δ 7.80 (dd, J = 5.3, 3.1 Hz, 2H), 7.71 (dd, J = 5.5, 3.0 Hz, 2H), 7.32 (d, J = 8.4 Hz, 2H), 7.18 (d, J = 8.4 Hz, 2H), 4.74 (d, J = 12.0 Hz, 1H), 4.60 (d, J = 12.0 Hz, 1H), 3.58 – 3.47 (m, 2H), 3.36 (d, J = 10.7 Hz, 1H), 2.33 – 2.23 (m, 1H), 1.76 – 1.67 (m, 1H), 1.59 – 1.48 (m, 1H), 1.23 – 1.17 (m, 1H), 1.05 (d, J = 6.6 Hz, 3H), 1.00 – 0.91 (m, 1H); **^{13}C NMR (101 MHz, $CDCl_3$)** δ 171.7, 168.4, 135.9, 134.1, 132.0, 131.9, 130.4, 123.3, 121.8, 94.8, 74.2, 57.7, 37.9, 35.8, 30.2, 25.0, 17.8; **IR:** (film) 2935, 1749, 1707, 1615, 1488, 1467, 1437, 1395, 1372, 1265, 1188, 1128, 1073, 1059, 1011 cm^{-1}; **HRMS (+p APCI)** calcd for $C_{23}H_{22}O_4N^{79}Br^{35}Cl_3$ $(M+H)^+$ 559.9792 found 559.9796. The enantiopurity was determined to be 94: 6 e.r. by chiral SFC analysis (Chiralcel OJ-3, 1% (50% methanol in isopropanol with 0.2% Formic Acid), 2.5 mL/min, λ = 230 nm, retention time = 9.31 (major) and 10.03 (minor) min). The absolute stereochemistry is tentatively assigned by analogy to the published C2 functionalization with Rh_2(*S*-2-Cl-5-BrTPCP)$_4$[14,15]

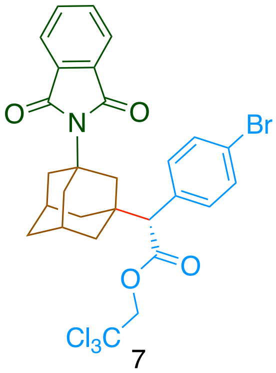

7

2,2,2-Trichloroethyl (*R*)-2-(4-bromophenyl)-2-((1*r*,3*S*,5*R*,7*S*)-3-(1,3-dioxoisoindolin-2-yl)adamantan-1-yl)acetate (7)

The general procedure C was employed for the C–H functionalization of 2-((3s,5s,7s)-adamantan-1-yl)isoindoline-1,3-dione (141 mg, 0.50 mmol) with 2,2,2-trichloroethyl 2-(4-bromophenyl)-2-diazoacetate (74.5 mg, 0.20 mmol) using Rh_2(*S*-TPPTTL)$_4$ (4.9 mg, 1 mol%) as catalyst, which was stirred in 2.0 mL dichloromethane at 23 °C for 3 hours. Flash chromatography (gradient 0 to 10% ethyl acetate/hexane) afforded the colorless oil as a major diastereomer (69.5 mg, 56 % yield).

$[\alpha]^{20}_D$: -4.25°(c = 0.68, CH_2Cl_2, 95: 5 e.r.); **^{1}H NMR (400 MHz, $CDCl_3$)** δ 7.77 – 7.72 (m, 2H), 7.70 – 7.64 (m, 2H), 7.46 (d, J = 8.6 Hz, 2H), 7.30 (d, J = 8.5 Hz, 2H), 4.82 (d, J = 12.0 Hz, 1H), 4.63 (d, J = 12.0 Hz, 1H), 3.51 (s, 1H), 2.48 – 2.33 (m, 6H), 2.27 – 2.20 (m, 2H), 1.83 – 1.67 (m, 3H), 1.65 – 1.50 (m, 3H, overlapping with signal from water); **^{13}C NMR (101 MHz, $CDCl_3$)** δ 170.5, 169.7, 133.9, 132.6, 132.0 (two carbons), 131.3, 122.7, 122.0, 94.8, 74.3, 61.8, 60.8, 42.4, 39.41, 39.37, 38.68, 38.70, 38.3, 35.3, 29.70, 29.68; **IR:** (film) 2915, 2851, 1747, 1704, 1612, 1489, 1467, 1453, 1410, 1367, 1343, 1312, 1212, 1160, 1124, 1075, 1045, 1029, 1011 cm^{-1}; **HRMS (+p APCI)** calcd for $C_{28}H_{26}O_4N^{79}Br^{35}Cl_3$ $(M+H)^+$ 624.0105 found 624.0111. The enantiopurity was determined to be 95: 5 e.r. by chiral HPLC analysis (Regis (R,R) Whelk-O 1, 2% isopropanol in hexane, 1.00 mL/min, λ = 230 nm, retention time = 27.73 (minor) and 30.19 (major) min). The absolute stereochemistry is tentatively assigned by analogy to the published asymmetric induction with Rh_2(*S*-TPPTTL)$_4$[1]

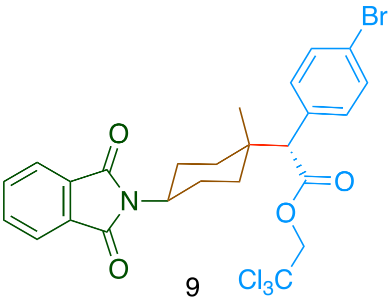

9

2,2,2-Trichloroethyl (*R*)-2-(4-bromophenyl)-2-((1*r*,4*R*)-4-(1,3-dioxoisoindolin-2-yl)-1-methylcyclohexyl)acetate (9)

The general procedure C was employed for the C–H functionalization of (cis/trans) 2-(4-methylcyclohexyl)isoindoline-1,3-dione (122 mg, 0.50 mmol) with 2,2,2-trichloroethyl 2-(4-bromophenyl)-2-diazoacetate (74.5 mg, 0.20 mmol) using Rh_2(*S*-TPPTTL)$_4$ (4.9 mg, 1 mol%) as catalyst, which was stirred in 2.0 mL dichloromethane at 23 °C for 3 hours. Flash chromatography (gradient 0 to 15% diethyl ether/hexane) afforded the white solid as a major diastereomer (69.5 mg, 56 % yield).

m.p. 132-134 °C; **$[\alpha]^{20}_D$**: -10.7°(c = 0.31, CH_2Cl_2, 3: 97 e.r.); **^{1}H NMR (400 MHz, $CDCl_3$)** δ 7.79 (dd, J = 5.5, 3.0 Hz, 2H), 7.69 (dd, J = 5.5, 3.1 Hz, 2H), 7.46 (d, J = 8.5 Hz, 2H), 7.31 (d, J = 8.5 Hz, 2H), 4.83 (d, J = 12.0 Hz, 1H), 4.61 (d, J = 12.0 Hz, 1H), 4.01 (tt, J = 12.4, 4.2 Hz, 1H), 3.55 (s, 1H), 2.53 – 2.34 (m, 2H, two axial diastereotopic α to nitrogen protons), 1.70 – 1.51 (m, 5H), 1.49 – 1.37 (m, 1H), 1.22 (s, 3H); **^{13}C NMR (101 MHz, $CDCl_3$)** δ 170.9, 168.5, 134.0, 133.4, 132.1, 132.0, 131.3, 123.2, 122.1, 94.8, 74.3, 62.4, 50.1, 36.8, 34.7, 34.5, 24.8, 24.7, 19.1; **IR:** (film) 2932, 1772, 1747, 1707, 1613, 1489, 1467, 1386, 1374, 1334, 1264, 1172, 1117, 1083, 1027, 1012 cm^{-1}; **HRMS (+p APCI)** calcd for $C_{25}H_{24}O_4N^{79}Br^{35}Cl_3$ $(M+H)^+$ 585.9949 found 585.9956. The enantiopurity was determined to be 3: 97 e.r. by chiral HPLC analysis (Regis (R,R) Whelk-O 1, 2% isopropanol in hexane, 1.00 mL/min, λ = 230 nm, retention time = 27.73 (minor) and 30.19 (major) min). The absolute stereochemistry is assigned by X-ray crystallography and is consistent with the published asymmetric induction with Rh_2(*S*-TPPTTL)$_4$[1]

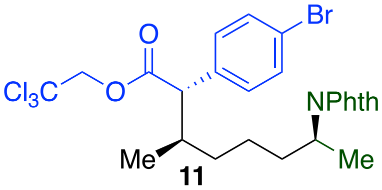

11

2,2,2-Trichloroethyl (2*S*,3*R*,7*S*)-2-(4-bromophenyl)-7-(1,3-dioxoisoindolin-2-yl)-3-methyloctanoate (11)

The general procedure C was employed for the C–H functionalization of (*S*)-2-(heptan-2-yl)isoindoline-1,3-dione (123 mg, 0.50 mmol) with 2,2,2-trichloroethyl 2-(4-bromophenyl)-2-diazoacetate (74.5 mg, 0.20 mmol) using **Rh_2(*S*-2-Cl-5-BrTPCP)$_4$** (3.8 mg, 1 mol%) as catalyst, which was stirred in 2.0 mL dichloromethane at 23 °C for 3 hours. Flash chromatography (gradient 0 to 8 to 10% ethyl acetate/hexane) afforded the colorless oil as a major diastereomer (Cerium Ammonium Molybdate stain was used for chromatography, 68.6 mg, 58 % combined yield).

$[\alpha]^{20}_D$: +2.9°(c = 0.66, CH_2Cl_2, 13: 83 d.r.); **^{1}H NMR (400 MHz, $CDCl_3$)** major diastereomer δ 7.80 (dd, J = 5.4, 3.0 Hz, 2H), 7.70 (dd, J = 5.5, 3.1 Hz, 2H), 7.41 (d, J = 8.5 Hz, 2H), 7.19 (d, J = 8.5 Hz, 2H), 4.74 (d, J = 12.0 Hz, 1H), 4.59 (d, J = 12.0 Hz, 1H), 4.30 – 4.18 (m, 1H), 3.33 (d, J = 10.6 Hz, 1H), 2.26 – 2.10 (m, 1H), 2.00 (dtd, J = 14.2, 9.9, 4.7 Hz, 1H), 1.56 – 1.44 (m, 1H), 1.40 (d, J = 6.9 Hz, 3H), 1.34 – 1.22 (m, 1H), 1.18 – 1.02 (m, 2H), 1.01 – 0.83 (m, 4H); **^{13}C NMR (101 MHz, $CDCl_3$)** δ 171.8, 168.6, 136.1, 134.0, 132.0, 131.9, 130.5, 123.2, 121.8, 94.8, 74.2, 58.2, 47.3, 36.1, 33.8, 33.0, 23.7, 18.9, 17.9; **IR:** (film) 2934, 1749, 1702, 1613, 1590, 1488, 1467, 1394, 1368, 1332, 1273, 1127, 1072, 1011 cm^{-1}; **HRMS (-p APCI)** calcd for $C_{25}H_{24}O_4N^{79}Br^{35}Cl_3$ $(M-H)^-$ 585.9960 found 585.9948 (Δ = -1.94 ppm). The diastereoselectivity was determined to be 13: 83 d.r. by analysis of crude ^{1}H NMR. The absolute configuration of the two new stereogenic centers is tentatively assigned by analogy to the published C2 functionalization with Rh_2(*S*-2-Cl-5-BrTPCP)$_4$[14,15]. The relative configuration is assignment based on the distinctive shielding of the methyl group[14]

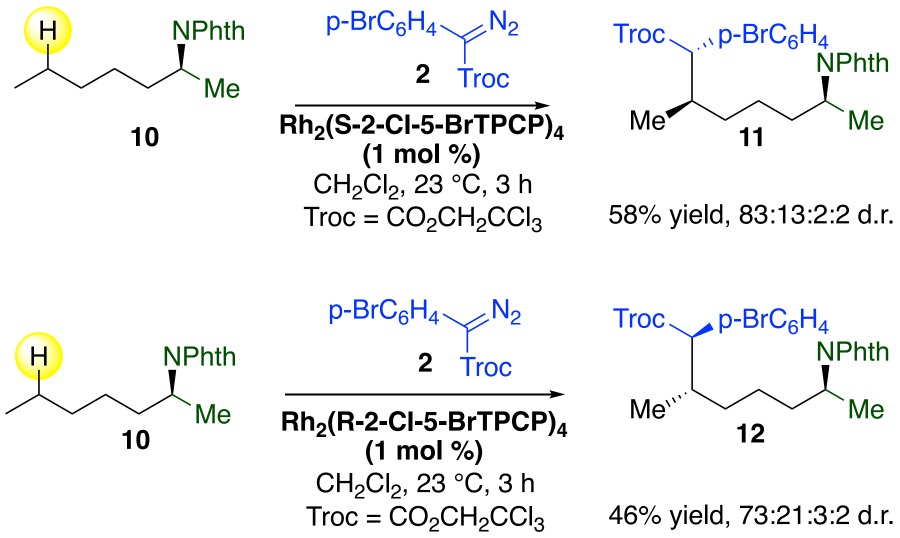

Note that the accurate diastereomer ratios were determined by analysis of crude ^{1}H NMR for the terminal methyl signals in **section 6** at **S48** and **S49**. (Benzylic signals only show two major diastereomers signals)

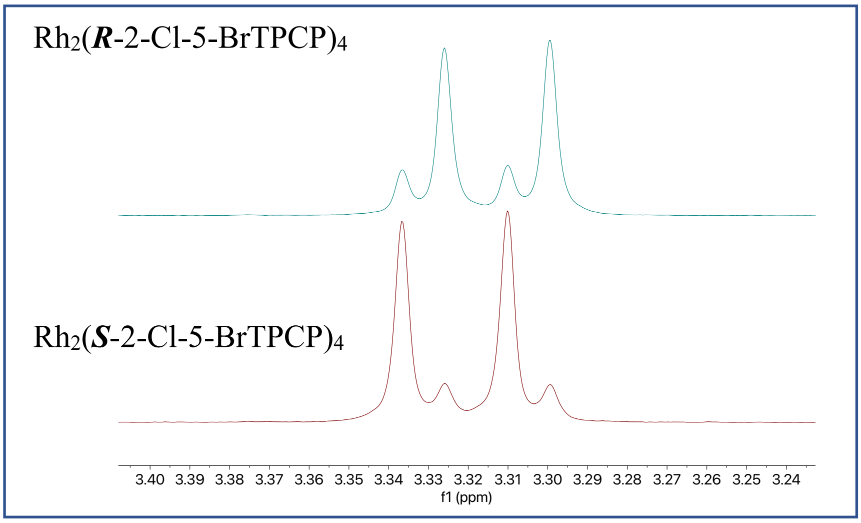

Zoomed in for benzylic signals

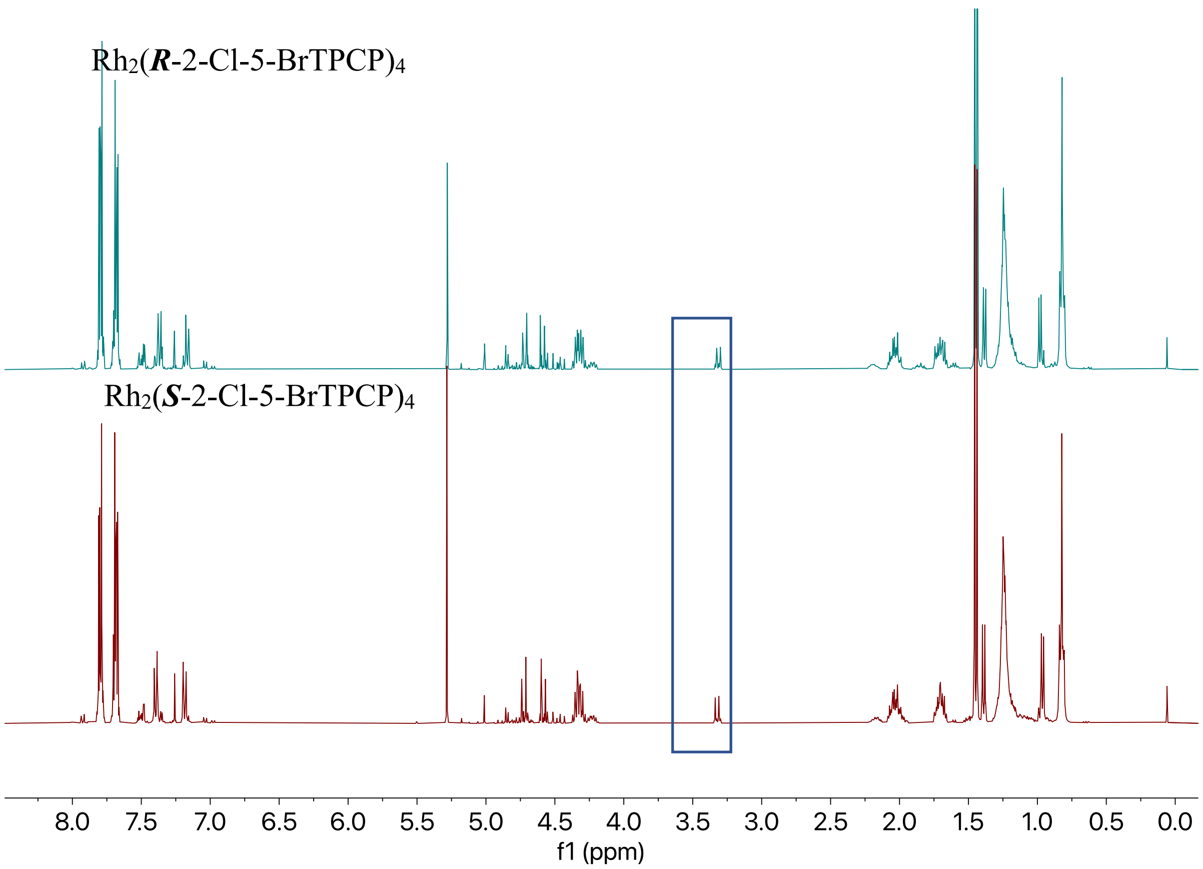

Rh2(R-2-Cl-5-BrTPCP)4
Rh2(S-2-Cl-5-BrTPCP)4
8.0 7.5 7.0 6.5 6.0 5.5 5.0 4.5 4.0 3.5 3.0 2.5 2.0 1.5 1.0 0.5 0.0
f1 (ppm)

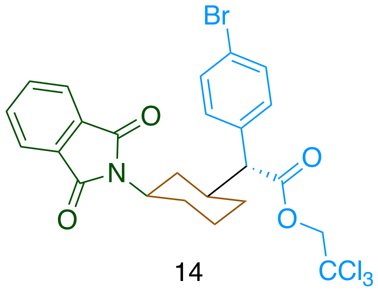

14

2,2,2-Trichloroethyl (*R*)-2-(4-bromophenyl)-2-((1*S*,3*R*)-3-(1,3-dioxoisoindolin-2-yl)cyclohexyl)acetate (14)

The general procedure was employed at 39 °C (to achieve higher yield) for the C–H functionalization of 2-cyclohexylisoindoline-1,3-dione (115 mg, 0.50 mmol) with 2,2,2-trichloroethyl 2-(4-bromophenyl)-2-diazoacetate (74.5 mg, 0.20 mmol) using Rh_2(*S*-TPPTTL)$_4$ (4.9 mg, 1 mol%) as catalyst, which was stirred in 2.0 mL dichloromethane at 39 °C in a heating mantle for 3 hours. The crude NMR indicated the presence of the desired product in a >20:1 d.r and 17:1 r.r. Flash chromatography (gradient 0 to 5% ethyl acetate/hexane) afforded the white solid as a major diastereomer (73 mg, 63 % yield).

Note that **Rh_2(*S*-BNP)$_4$ [*S*-BNP: *S*-binaphtholphosphate]**, which was tested with low regioselectivity, was used to determine the benzylic proton signal for C5 (3.43 ppm, d, J = 10.8 Hz) and C4 (3.35 ppm, d, J = 10.8 Hz) C–H functionalizaiton products.

$[\alpha]^{20}_D$: -38.5°(c = 0.16, CH_2Cl_2, 97: 3 e.r.); **^{1}H NMR (400 MHz, $CDCl_3$)** major diastereomer δ 7.76 (dd, J = 5.5, 3.0 Hz, 2H), 7.66 (dd, J = 5.5, 3.1 Hz, 2H), 7.41 (d, J = 8.5 Hz, 2H), 7.22 (d, J = 8.5 Hz, 2H), 4.77 (d, J = 11.9 Hz, 1H), 4.65 (d, J = 11.9 Hz, 1H), 4.08 (tt, J = 12.3, 3.9 Hz, 1H), 3.42 (d, J = 10.8 Hz, 1H), 2.30 – 2.19 (m, 1H), 2.19 – 2.08 (m, 1H), 1.98 – 1.89 (m, 2H), 1.82 (q, J = 12.3 Hz, 1H), 1.73 (d, J = 12.6 Hz, 1H), 1.47 (qt, J = 14.3, 3.6 Hz, 1H), 1.33 (d, J = 12.4 Hz, 1H), 1.24 – 1.14 (m, 1H); **^{13}C NMR (101 MHz, $CDCl_3$)** δ 171.4, 168.4, 135.3, 134.0, 132.04, 132.01, 130.5, 123.2, 122.0, 94.8, 74.3, 58.0, 50.1, 40.5, 33.2, 30.7, 29.4, 25.0; **IR:** (film) 2932, 2857, 1748, 1704, 1613, 1488, 1467, 1450, 1395, 1373, 1332, 1261, 1235, 1208, 1189, 1150, 1126, 1069, 1039, 1011cm^{-1}; **HRMS (+p APCI)** calcd for $C_{24}H_{22}O_4N^{79}Br^{35}Cl_3$ $(M+H)^+$ 571.9798 found 571.9796. The enantiopurity was determined to be 97: 3 e.r. by SFC analysis. (ChiralCel OJ-3, 5% (50% methanol in isopropanol with 0.2% Formic Acid), 2.5 mL/min, λ = 230 nm, retention time = 4.33 (minor) and 7.74 (major) min). The absolute stereochemistry is tentatively assigned by analogy to the published C2 functionalization with Rh_2(*S*-2-Cl-5-BrTPCP)$_4$[14,15]

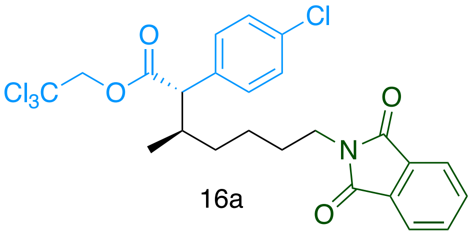

2,2,2-Trichloroethyl (2*S*,3*R*)-2-(4-chlorophenyl)-7-(1,3-dioxoisoindolin-2-yl)-3-methylheptanoate (16a)

The general procedure was employed for the C–H functionalization of 2-hexylisoindoline-1,3-dione (116 mg, 0.50 mmol) with 2,2,2-trichloroethyl 2-(4-chlorophenyl)-2-diazoacetate (65.6 mg, 0.20 mmol) using Rh_2(*S*-2-Cl-5-BrTPCP)$_4$ (3.8 mg, 1 mol%) as catalyst, which was stirred in 2.0 mL dichloromethane at 23 °C for 3 hours. The crude NMR indicated the presence of the desired product in a 40:1 d.r. Flash chromatography (gradient 0 to 10 to 15% diethyl ether/hexane) afforded the colorless oil as a major diastereomer (72.3 mg, 69 % yield).

$[\alpha]^{20}_D$: +12.2°(c = 0.32, CH_2Cl_2, 92: 8 e.r.); **^{1}H NMR (400 MHz, $CDCl_3$)** major diastereomer δ 7.83 (dd, J = 5.5, 3.0 Hz, 2H), 7.71 (dd, J = 5.5, 3.0 Hz, 2H), 7.32 – 7.24 (m, 4H), 4.76 (d, J = 12.0 Hz, 1H), 4.63 (d, J = 12.0 Hz, 1H), 3.59 (t, J = 7.2 Hz, 2H), 3.38 (d, J = 10.6 Hz, 1H), 2.30 – 2.18 (m, 1H), 1.64 – 1.53 (m, 1H), 1.53 – 1.44 (m, 1H), 1.43 – 1.31 (m, 1H), 1.24 – 1.15 (m, 2H), 1.05 (d, J = 6.5 Hz, 3H), 1.01 – 0.91 (m, 1H); **^{13}C NMR (101 MHz, $CDCl_3$)** δ 171.9, 168.5, 135.6, 134.0, 133.6, 132.2, 130.1, 128.9, 123.3, 94.8, 74.2, 58.1, 37.9, 36.1, 32.9, 28.7, 23.8, 17.9; **IR:** (film) 2937, 2861, 1748, 1706, 1616, 1491, 1467, 1437, 1395, 1368, 1273, 1188, 1126, 1090, 1062, 1015 cm^{-1}; **HRMS (-p APCI)** calcd for $C_{24}H_{22}O_4N^{35}Cl_4$ (M-H)$^-$ 528.0308 found 528.0293 (Δ = -2.99 ppm). The enantiopurity was determined to be 92: 8 e.r. by SFC analysis. (Regis (S,S)Whelk-O 1, 5% (50% methanol in isopropanol with 0.2% Formic Acid), 2.5 mL/min, λ = 230 nm, retention time = 11.14 (major) and 12.16 (minor) min). The absolute stereochemistry is tentatively assigned by analogy to the published C2 functionalization with Rh_2(*S*-2-Cl-5-BrTPCP)$_4$[14,15]

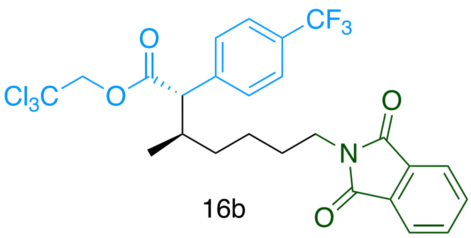

16b

2,2,2-Trichloroethyl (2*S*,3*R*)-7-(1,3-dioxoisoindolin-2-yl)-3-methyl-2-(4-(trifluoromethyl)phenyl)heptanoate (16b)

The general procedure was employed for the C–H functionalization of 2-hexylisoindoline-1,3-dione (116 mg, 0.50 mmol) with 2,2,2-trichloroethyl 2-diazo-2-(4-(trifluoromethyl)phenyl)acetate (72.3 mg, 0.20 mmol) using Rh_2(*S*-2-Cl-5-BrTPCP)$_4$ (3.8 mg, 1 mol%) as catalyst, which was stirred in 2.0 mL dichloromethane at 23 °C for 3 hours. The crude NMR indicated the presence of the desired product in a 20:1 d.r. Flash chromatography (gradient 0 to 10 to 15% diethyl ether/hexane) afforded the colorless oil as a major diastereomer (88.4 mg, 79 % yield).

$[\alpha]^{20}_D$: +11.3°(c = 0.41, CH_2Cl_2, 97: 3 e.r.); **^{1}H NMR (400 MHz, $CDCl_3$)** major diastereomer δ 7.82 (dd, J = 5.5, 3.0 Hz, 2H), 7.70 (dd, J = 5.5, 3.0 Hz, 2H), 7.56 (d, J = 8.1 Hz, 2H), 7.47 (d, J = 8.2 Hz, 2H), 4.77 (d, J = 12.0 Hz, 1H), 4.62 (d, J = 12.0 Hz, 1H), 3.58 (t, J = 7.2 Hz, 2H), 3.48 (d, J = 10.6 Hz, 1H), 2.35 – 2.23 (m, 1H), 1.61 – 1.52 (m, 1H), 1.52 – 1.43 (m, 1H), 1.43 – 1.31 (m, 1H), 1.23 – 1.13 (m, 2H), 1.06 (d, J = 6.5 Hz, 3H), 1.03 – 0.92 (m, 1H); **^{13}C NMR (101 MHz, $CDCl_3$)** δ 171.5, 168.5, 141.1, 134.1, 132.2, 130.1 (q, J = 32.6 Hz), 129.2, 125.7 (q, J = 3.7 Hz), 124.1 (q, J = 272.1 Hz, -CF_3), 123.3, 94.8, 74.3, 58.5, 37.9, 36.2, 33.0, 28.7, 23.8, 17.9; **^{19}F NMR (376 MHz, $CDCl_3$, ^{1}H decoupled)** δ - 62.5; **IR:** (film) 2938, 1750, 1708, 1618, 1467, 1437, 1421, 1396, 1369, 1323, 1164, 1122, 1067, 1018 cm^{-1}; **HRMS (-p APCI)** calcd for $C_{25}H_{22}O_4N^{35}Cl_3F_3$ $(M\text{-}H)^-$ 562.0572 found 562.0563 (Δ = -1.66 ppm). The enantiopurity was determined to be 97: 3 e.r. by chiral SFC analysis (Trefoil AMY1, 10% (50% methanol in isopropanol with 0.2% Formic Acid), 2.50 mL/min, λ = 210 nm, retention time = 1.71 (minor) and 2.57 (major) min). The absolute stereochemistry is tentatively assigned by analogy to the published C2 functionalization with Rh_2(*S*-2-Cl-5-BrTPCP)$_4$[14,15]

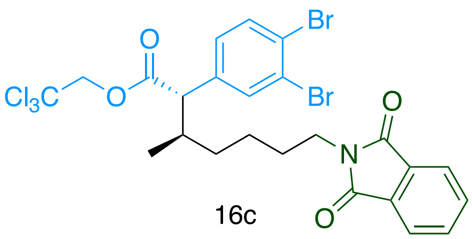

16c

2,2,2-Trichloroethyl (2*S*,3*R*)-2-(3,5-dibromophenyl)-7-(1,3-dioxoisoindolin-2-yl)-3-methylheptanoate (16c)

The general procedure was employed for the C–H functionalization of 2-hexylisoindoline-1,3-dione (116 mg, 0.50 mmol) with 2,2,2-trichloroethyl 2-diazo-2-(3,4-dibromophenyl)acetate (90.3 mg, 0.20 mmol) using Rh_2(*S*-2-Cl-5-BrTPCP)$_4$ (3.8 mg, 1 mol%) as catalyst, which was stirred in 2.0 mL dichloromethane at 23 °C for 3 hours. The crude NMR indicated the presence of the desired product in a 20:1 d.r. Flash chromatography (gradient 0 to 10 to 15% diethyl ether/hexane) afforded the colorless oil as a major diastereomer (106.9 mg, 81 % yield).

$[\alpha]^{20}_{D}$: +7.8°(c = 0.31, CH_2Cl_2, 95: 5 e.r.); **^{1}H NMR (400 MHz, $CDCl_3$)** major diastereomer δ 7.83 (dd, J = 5.5, 3.0 Hz, 2H), 7.70 (dd, J = 5.5, 3.0 Hz, 2H), 7.63 (d, J = 2.1 Hz, 1H), 7.54 (d, J = 8.3 Hz, 1H), 7.16 (dd, J = 8.3, 2.1 Hz, 1H), 4.78 (d, J = 12.0 Hz, 1H), 4.61 (d, J = 12.0 Hz, 1H), 3.60 (t, J = 7.2 Hz, 2H), 3.34 (d, J = 10.6 Hz, 1H), 2.27 – 2.14 (m, 1H), 1.61 – 1.54 (m, 1H), 1.54 – 1.45 (m, 1H), 1.43 – 1.31 (m, 1H), 1.24 – 1.15 (m, 2H), 1.03 (d, J = 6.5 Hz, 3H), 1.00 – 0.90 (m, 1H); **^{13}C NMR (101 MHz, $CDCl_3$)** δ 171.2, 168.5, 138.0, 134.0, 133.93, 133.87, 132.2, 129.0, 125.2, 124.2, 123.3, 94.7, 74.3, 57.8, 37.9, 36.2, 33.0, 28.7, 23.8, 17.8; **IR:** (film) 2936, 2860, 1749, 1706, 1615, 1556, 1464, 1395, 1368, 1270, 1188, 1129, 1061, 1014 cm^{-1}; **HRMS (-p APCI)** calcd for $C_{24}H_{21}O_4N^{79}Br_2{}^{35}Cl_3$ $(M-H)^-$ 649.8908 found 649.8903 (Δ = -0.91 ppm). The enantiopurity was determined to be 95: 5 e.r. by chiral HPLC analysis (ChiralPak ADH, 1% isopropanol in hexane, 1.0 mL/min, λ = 230 nm, retention time = 37.4 (major) and 43.0 (minor) min). The absolute stereochemistry is tentatively assigned by analogy to the published C2 functionalization with Rh_2(*S*-2-Cl-5-BrTPCP)$_4$[14,15]

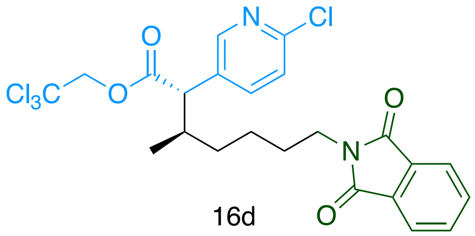

16d

2,2,2-Trichloroethyl (2*S*,3*R*)-2-(6-chloropyridin-3-yl)-7-(1,3-dioxoisoindolin-2-yl)-3-methylheptanoate (16d)

The general procedure was employed for the C–H functionalization of 2-hexylisoindoline-1,3-dione (116 mg, 0.50 mmol) with 2,2,2-trichloroethyl 2-(6-chloropyridin-3-yl)-2-diazoacetate (65.8 mg, 0.20 mmol) using Rh_2(*S*-2-Cl-5-BrTPCP)$_4$ (3.8 mg, 1 mol%) as catalyst, which was stirred in 2.0 mL dichloromethane at 23 °C for 3 hours. The crude NMR indicated the presence of the desired product in a 28:1 d.r. Flash chromatography (gradient 0 to 15 to 25% diethyl ether/hexane) afforded the colorless oil as a major diastereomer (45.8 mg, 44 % yield).

$[\alpha]^{20}_D$: -0.5°(c = 0.23, CH_2Cl_2, 87: 13 e.r.); **^{1}H NMR (400 MHz, $CDCl_3$)** major diastereomer δ 8.31 (d, J = 2.3 Hz, 1H), 7.82 (dd, J = 5.4, 3.1 Hz, 2H), 7.73 (dd, J = 8.3, 2.5 Hz, 1H), 7.70 (dd, J = 5.5, 3.0 Hz, 2H), 7.30 (d, J = 8.3 Hz, 1H), 4.76 (d, J = 12.0 Hz, 1H), 4.65 (d, J = 12.0 Hz, 1H), 3.58 (t, J = 7.1 Hz, 2H), 3.44 (d, J = 10.2 Hz, 1H), 2.29 – 2.16 (m, 1H), 1.64 – 1.45 (m, 2H), 1.44 – 1.31 (m, 1H), 1.23 – 1.13 (m, 2H), 1.05 (d, J = 6.5 Hz, 3H), 1.01 – 0.92 (m, 1H); **^{13}C NMR (101 MHz, $CDCl_3$)** δ 171.1, 168.5, 150.1, 138.8, 134.1, 132.2, 124.5, 123.4, 94.6, 74.4, 55.2, 37.8, 36.3, 33.0, 28.6, 23.8, 17.8; **IR:** (film) 2935, 1749, 1706, 1584, 1565, 1460, 1437, 1396, 1369, 1189, 1138, 1104, 1062, 1023 cm^{-1}; **HRMS (+p APCI)** calcd for $C_{23}H_{23}O_4N_2{}^{35}Cl_4$ $(M+H)^+$ 531.0406 found 531.0415. The enantiopurity was determined to be 87: 13 e.r. by chiral SFC analysis (Chiralcel OJ-3, 4% (50% methanol in isopropanol with 0.2% Formic Acid), 2.50 mL/min, λ 280 nm, retention time = 5.81 (minor) and 6.25 (major) min). The absolute stereochemistry is tentatively assigned by analogy to the published C2 functionalization with Rh_2(*S*-2-Cl-5-BrTPCP)$_4$[14,15]

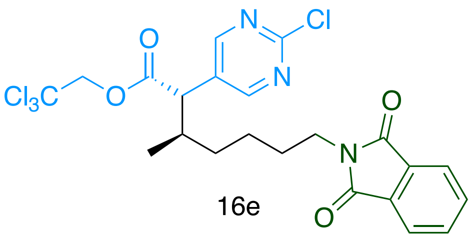

16e

2,2,2-Trichloroethyl (2*S*,3*R*)-2-(2-chloropyrimidin-5-yl)-7-(1,3-dioxoisoindolin-2-yl)-3-methylheptanoate (16e)

The general procedure was employed for the C–H functionalization of 2-hexylisoindoline-1,3-dione (116 mg, 0.50 mmol) with 2,2,2-trichloroethyl 2-(2-chloropyrimidin-5-yl)-2-diazoacetate (66.0 mg, 0.20 mmol) using Rh_2(*S*-2-Cl-5-BrTPCP)$_4$ (3.8 mg, 1 mol%) as catalyst, which was stirred in 2.0 mL dichloromethane at 23 °C for 3 hours. The crude NMR indicated the presence of the desired product in a 15:1 d.r. Flash chromatography (gradient 0 to 15 to 25% diethyl ether/hexane) afforded the colorless oil as a major diastereomer (47.2 mg, 44 % yield).

$[\alpha]^{20}_D$: -3.3°(c = 0.22, CH_2Cl_2, 88: 12 e.r.); **^{1}H NMR (400 MHz, $CDCl_3$)** major diastereomer δ 8.64 (s, 2H), 7.82 (dd, J = 5.5, 3.0 Hz, 2H), 7.70 (dd, J = 5.5, 3.0 Hz, 2H), 4.78 (d, J = 11.9 Hz, 1H), 4.68 (d, J = 11.9 Hz, 1H), 3.60 (t, J = 7.0 Hz, 2H), 3.48 (d, J = 9.6 Hz, 1H), 2.30 – 2.18 (m, 1H), 1.66 – 1.47 (m, 2H), 1.46 – 1.33 (m, 1H), 1.32 – 1.15 (m, 2H, overlapping with "grease" at 1.2 ppm), 1.10 – 0.97 (m, 4H); **^{13}C NMR (101 MHz, $CDCl_3$)** δ 170.2, 168.5, 159.8, 134.1, 132.1, 129.4, 123.4, 94.4, 74.6, 53.2, 37.6, 36.6, 33.0, 28.5, 23.8, 17.7; **IR:** (film) 2927, 2857, 1750, 1706, 1615, 1577, 1546, 1467, 1437, 1396, 1369, 1266, 1188, 1154, 1061 cm^{-1}; **HRMS (+p APCI)** calcd for $C_{22}H_{22}O_4N_3{}^{35}Cl_4$ $(M+H)^+$ 532.0359 found 532.0370. The enantiopurity was determined to be 88:12 e.r. by chiral SFC analysis (Chiralcel OZ-3, 5% (50% methanol in isopropanol with 0.2% Formic Acid), 2.50 mL/min, λ = 230 nm, retention time = 25.7 (minor) and 28.8 (major) min). The absolute stereochemistry is tentatively assigned by analogy to the published C2 functionalization with Rh_2(*S*-2-Cl-5-BrTPCP)$_4$[14,15]

4. Reference

1. Fu, J.; Ren, Z.; Bacsa, J.; Musaev, D. G. Davies, H. M. L. *Nature*. **2018**, *564*, 395.
2. Fu, L.; Hoang, K.; Tortoreto, C.; Liu, W.; Davies, H. M. L. *Org. Lett.* **2018**, *20*, 2399.
3. Khedkar, M. V.; Khan, S. R.; Sawant, D. N.; Bagal, D. B.; Bhanage, B. M. *Adv. Synth. Catal.* **2011**, *353*, 3415.
4. Fu, L.; Mighion, J. D.; Voight, E. A.; Davies, H. M. L. *Chem. Eur. J.* **2017**, *23*, 3272.
5. Fraga-Dubreuil, J.; Çomak, G.; Taylora, A. W.; Poliakoff, M. *Green Chem.,* **2007**, *9*, 1067.
6. Zhang, J.; Senthilkumar, M.; Ghosh, S. C.; Hong, S. H. *Angew. Chem. Int. Ed.* **2010**, *49*, 6391.
7. Wang, M. L.; Chen, W. H. *React. Kinet. Catal. Lett.*, **2006**, *89*, 377.
8. Parker, K. A.; O'Fee, R. P. *J. Org. Chem.* **1983**, *48*, 9, 1547.
9. Sultane, P. R.; Ahumada, G.; Janssen-Müller, D.; Bielawski, C. W. *Angew. Chem. Int. Ed.* **2019**, *58*, 16320.
10. Lia, Xin.; Shi, Z.-J. *Org. Chem. Front.,* **2016**, *3*, 1326.
11. Dhake, K. P.; Qureshi, Z. S.; Singhal, R. S.; Bhanage, B. M. *Tetrahedron. Lett.* **2009**, *50*, 2811.
12. Ishihara, K.; Yano, T. *Org. Lett.* **2004**, *6*, 12, 1983.
13. Dennes, T. J.; Schwartz, J. *Soft Matter*. **2008**, *4*, 86.
14. Ren, P.; Davies, H. M. L. *Tetrahedron. Lett.* **2001**, *42*, 3149.
15. Liu, W.; Ren, Z.; Bosse, A. T.; Liao, K.; Goldstein, E. L.; Basca, J.; Musaev D. G.; Stoltz, B. M.; Davies, H. M. L. *J. Am. Chem. Soc.* **2018**, *140*, 12247.

5. NMR Spectra

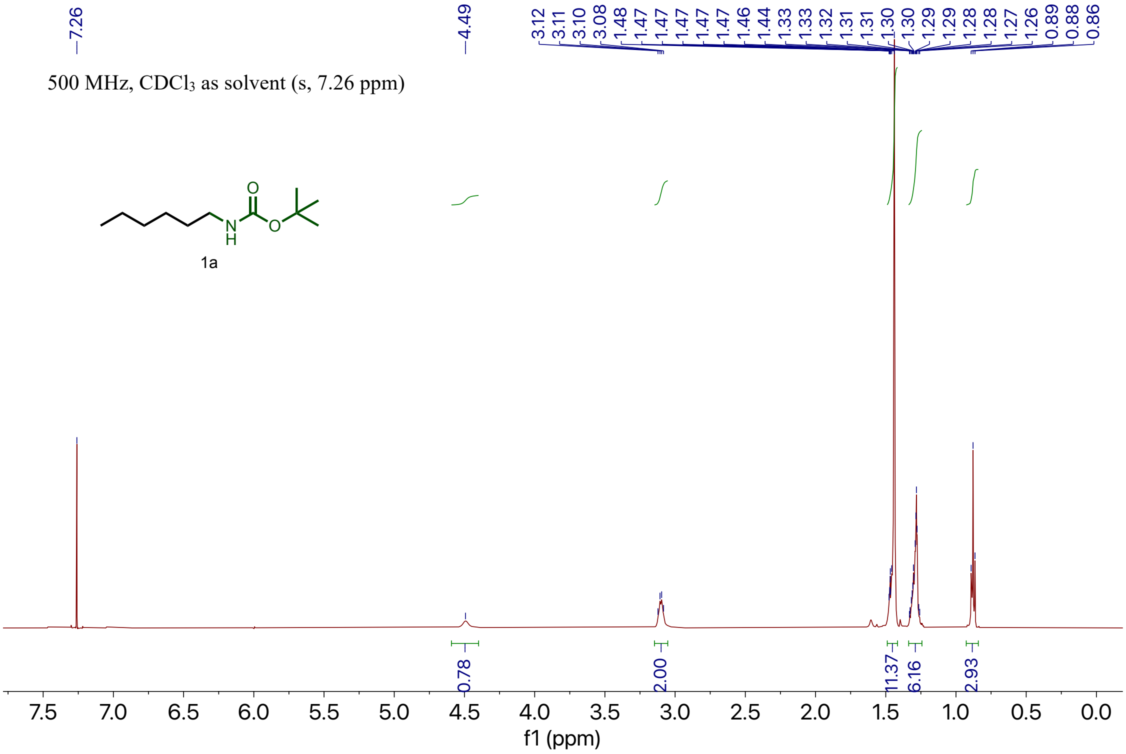

500 MHz, $CDCl_3$ as solvent (s, 7.26 ppm)

1a

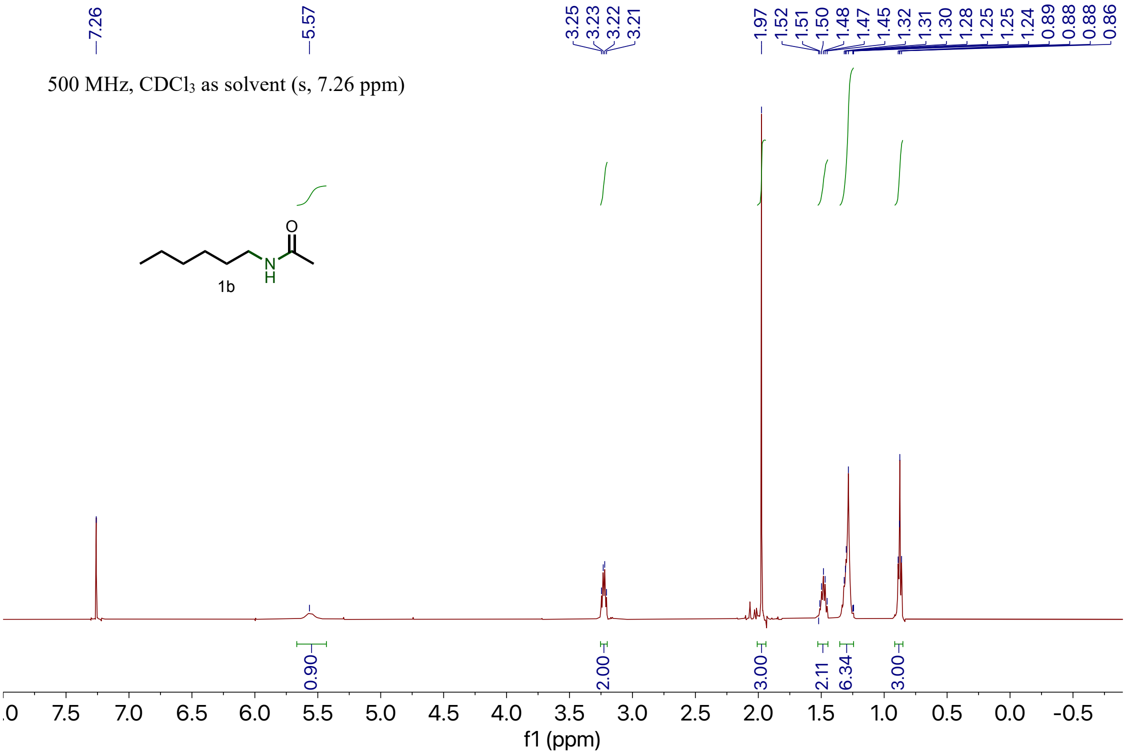

500 MHz, $CDCl_3$ as solvent (s, 7.26 ppm)

1b

500 MHz, $CDCl_3$ as solvent (s, 7.26 ppm)

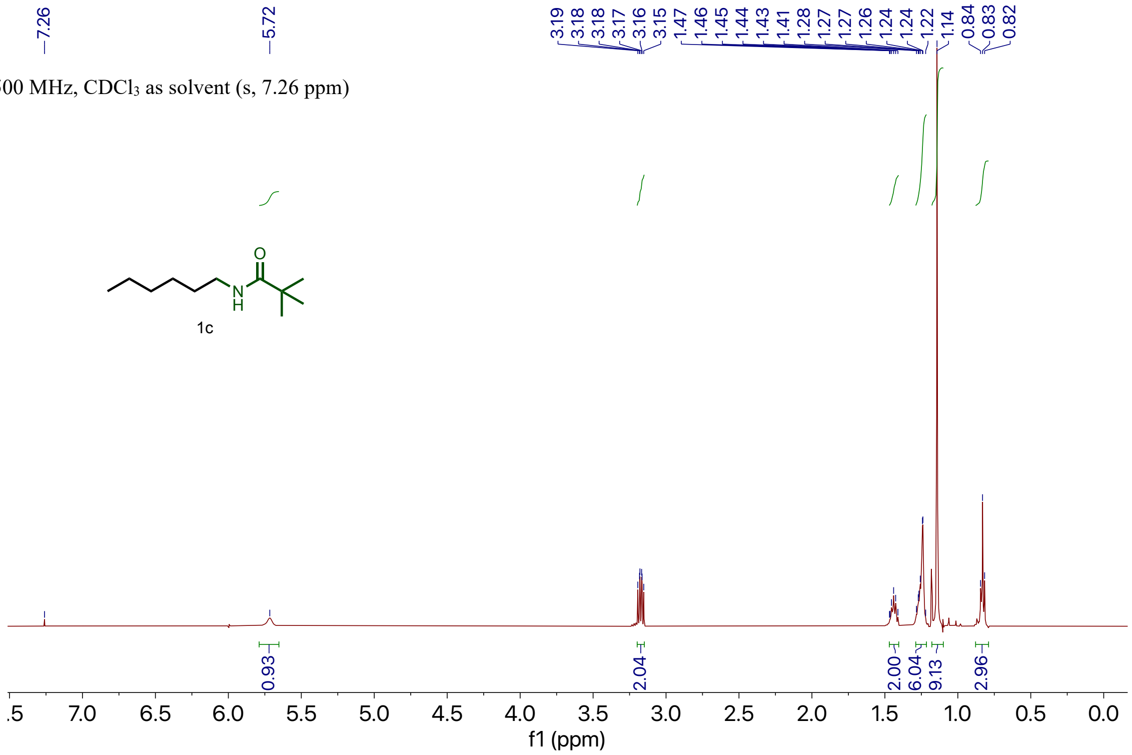

500 MHz, $CDCl_3$ as solvent (s, 7.26 ppm)

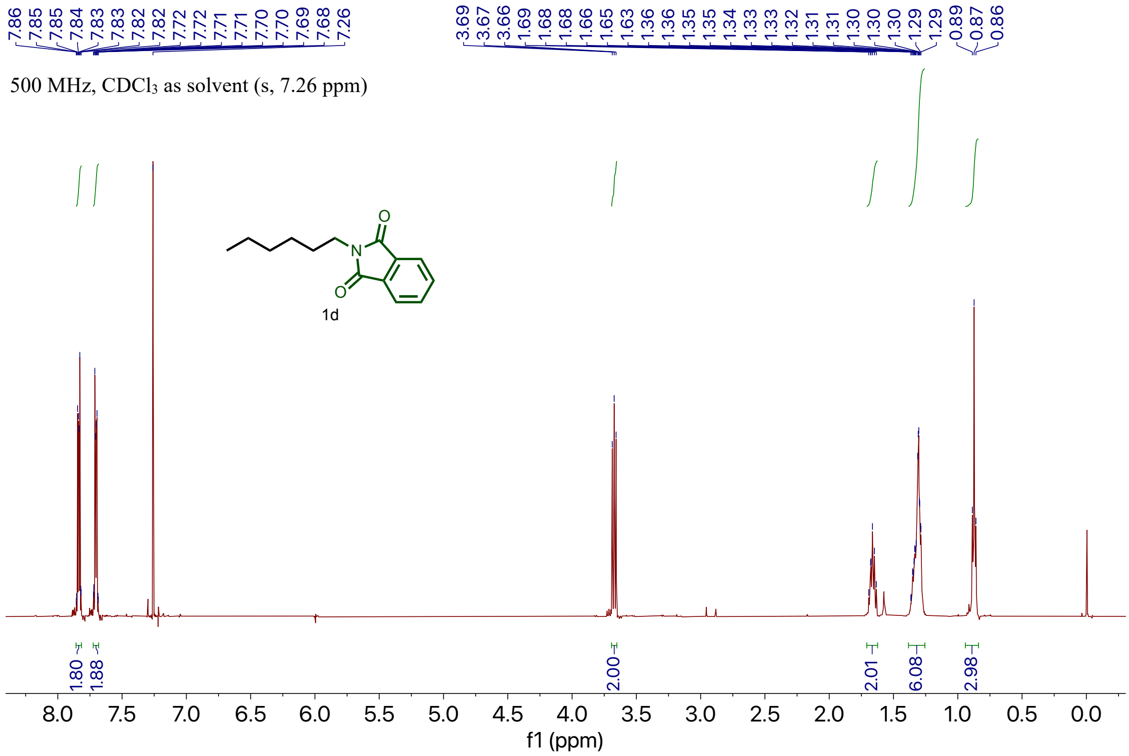

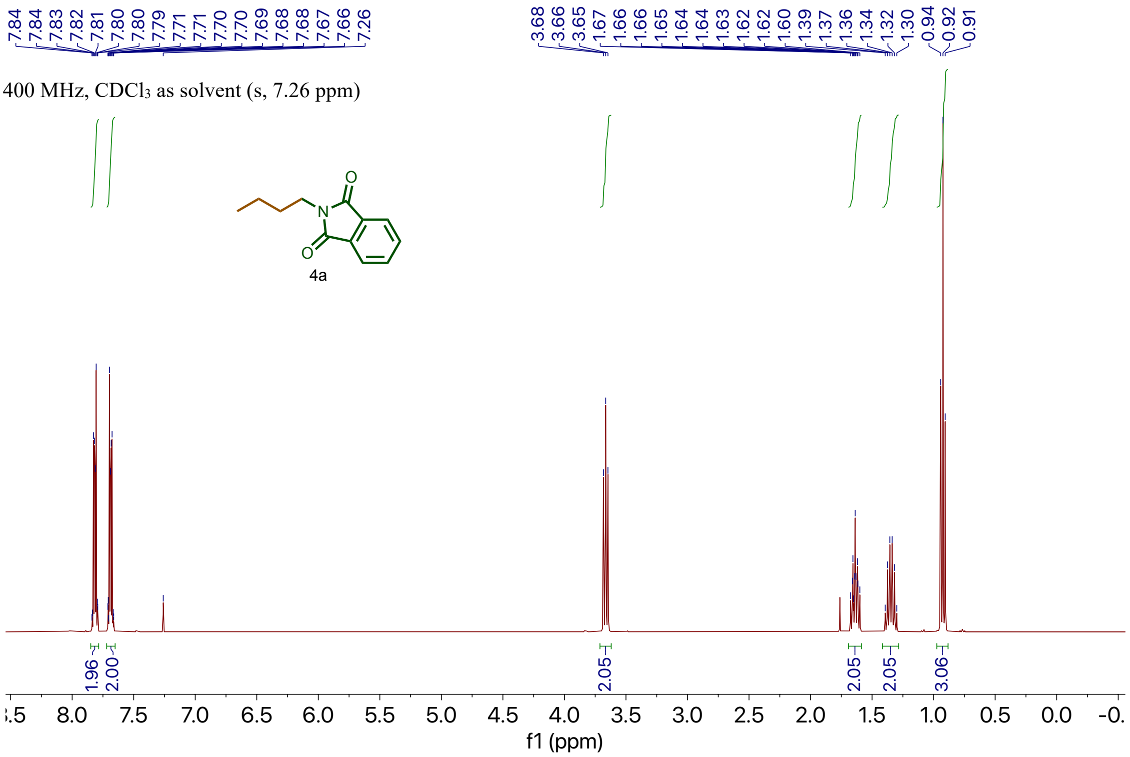
400 MHz, CDCl3 as solvent (s, 7.26 ppm)
4a
f1 (ppm)

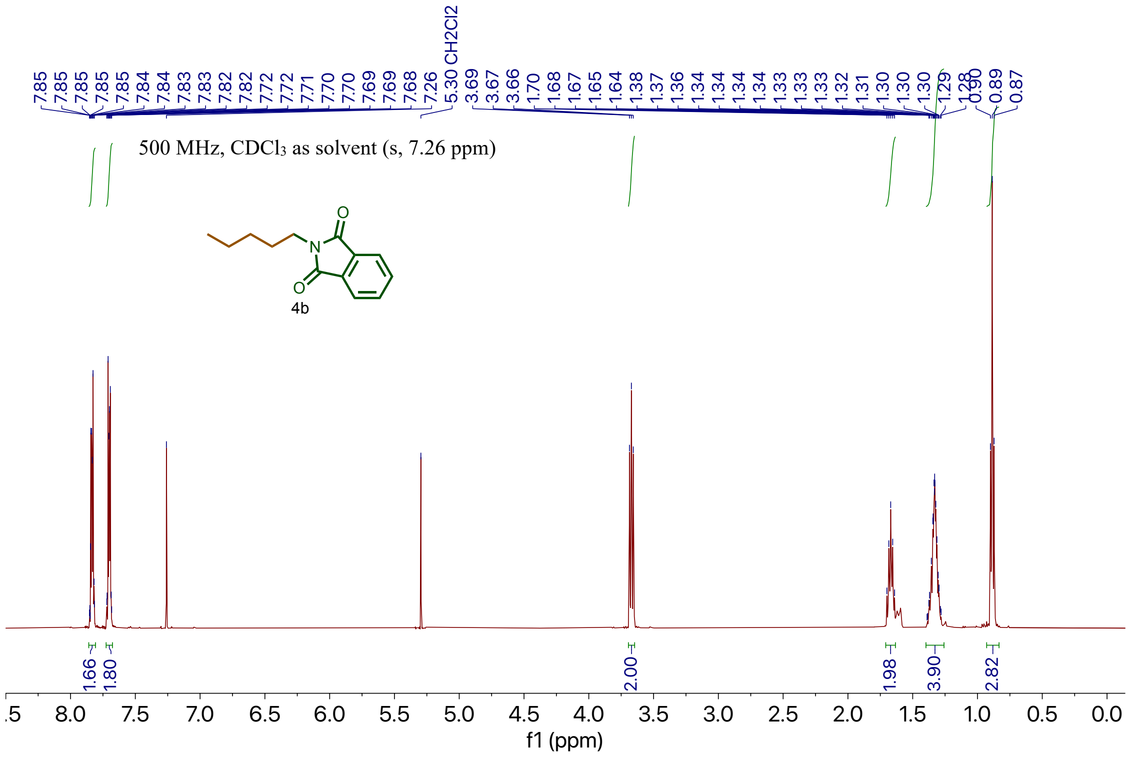
500 MHz, CDCl3 as solvent (s, 7.26 ppm)
4b
CH2Cl2
f1 (ppm)

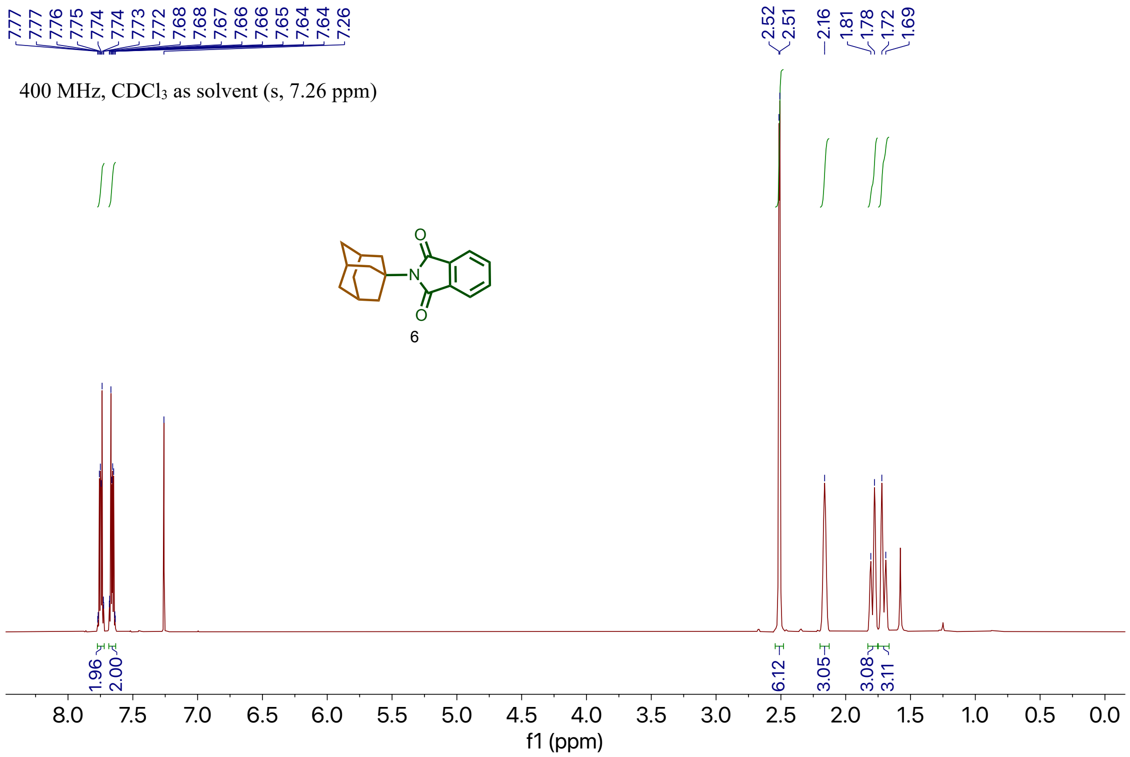
400 MHz, CDCl3 as solvent (s, 7.26 ppm)
6
f1 (ppm)

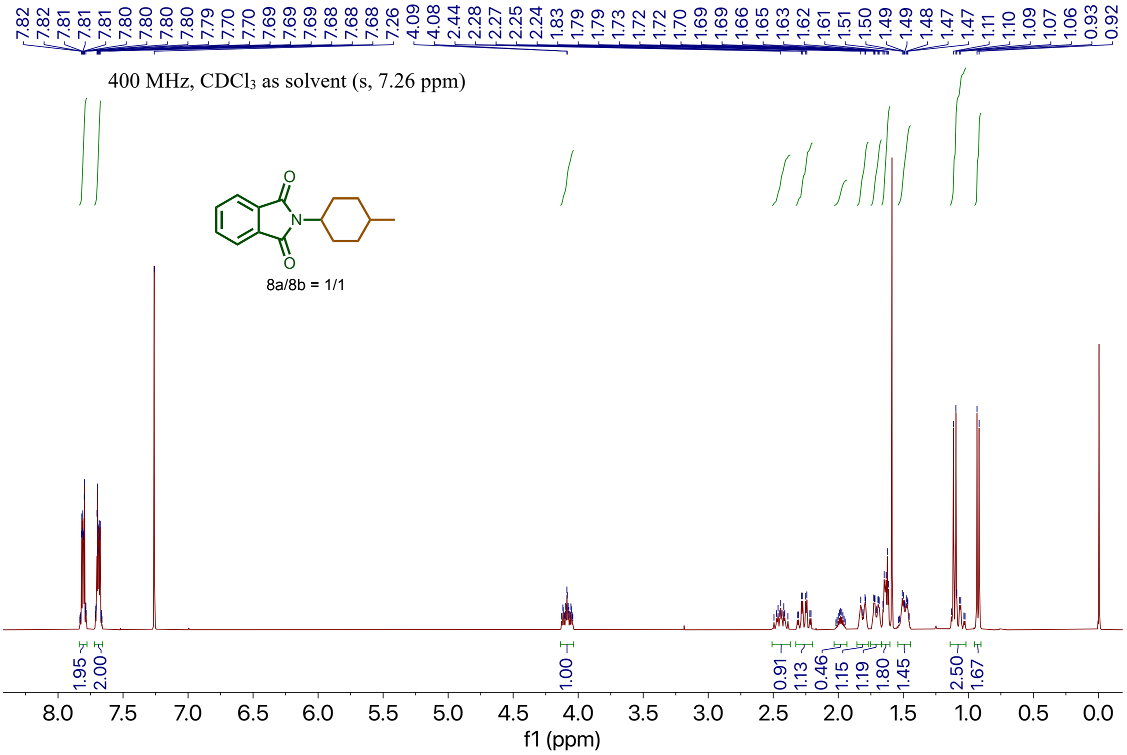
400 MHz, CDCl3 as solvent (s, 7.26 ppm)
8a/8b = 1/1
f1 (ppm)

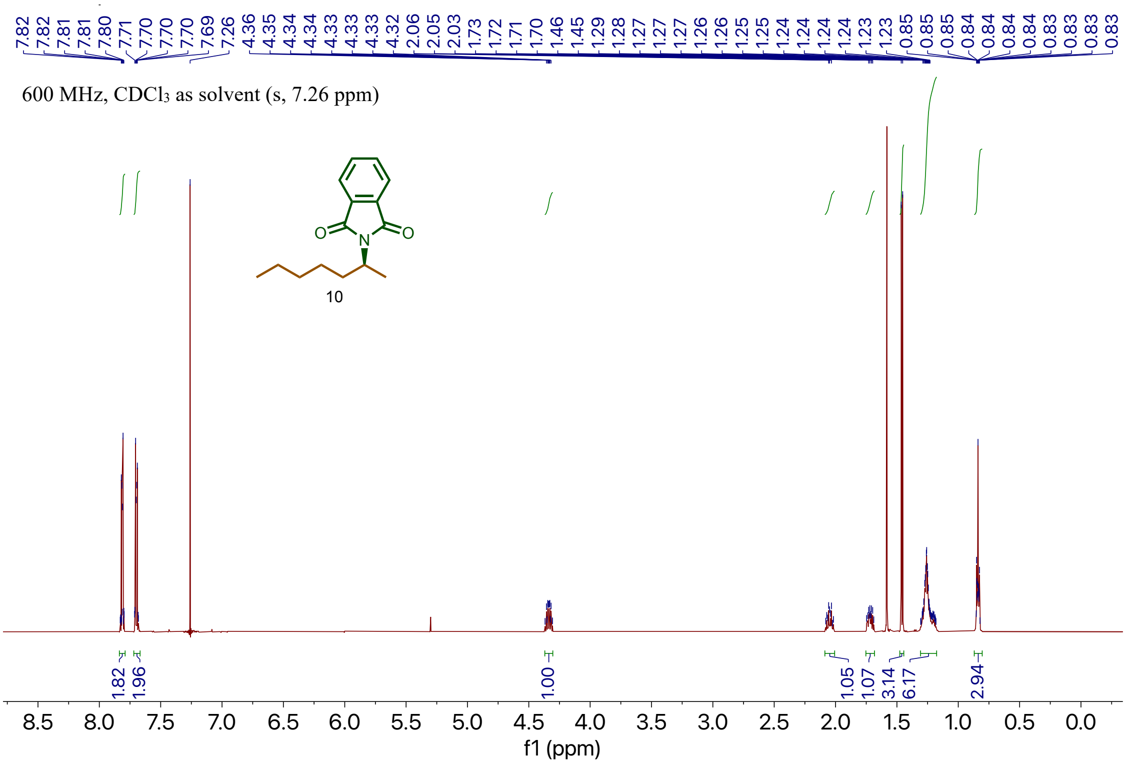

600 MHz, CDCl3 as solvent (s, 7.26 ppm)
10
f1 (ppm)

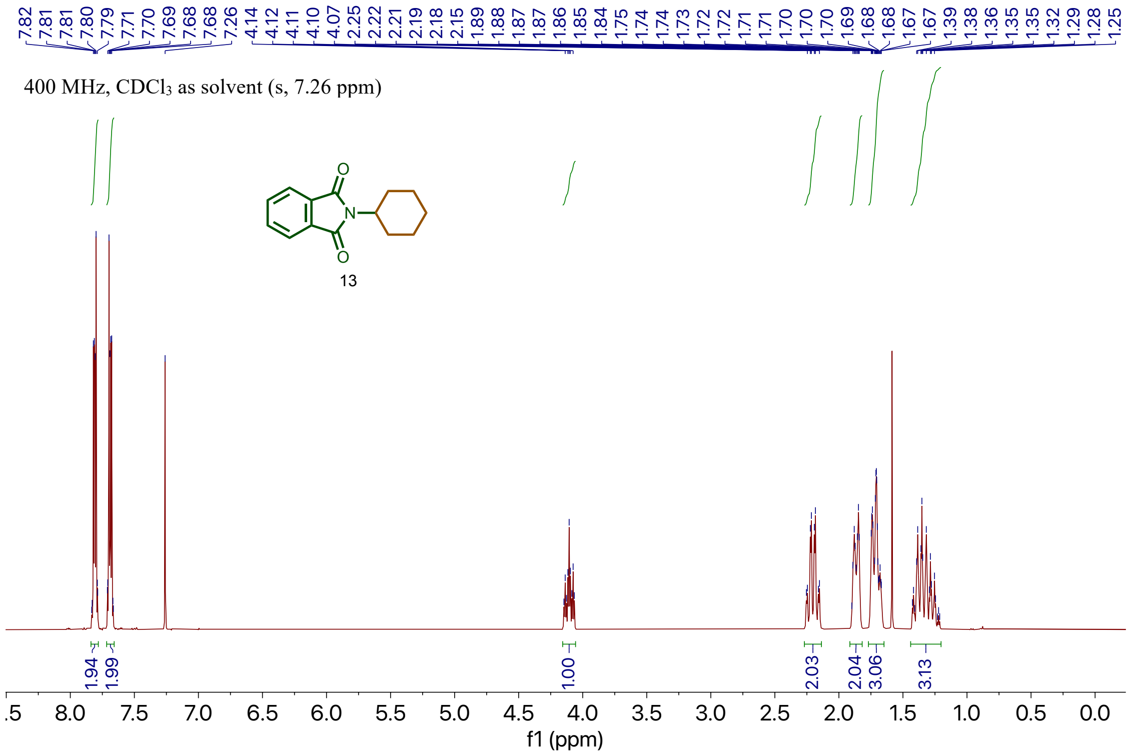

400 MHz, CDCl3 as solvent (s, 7.26 ppm)
13
f1 (ppm)

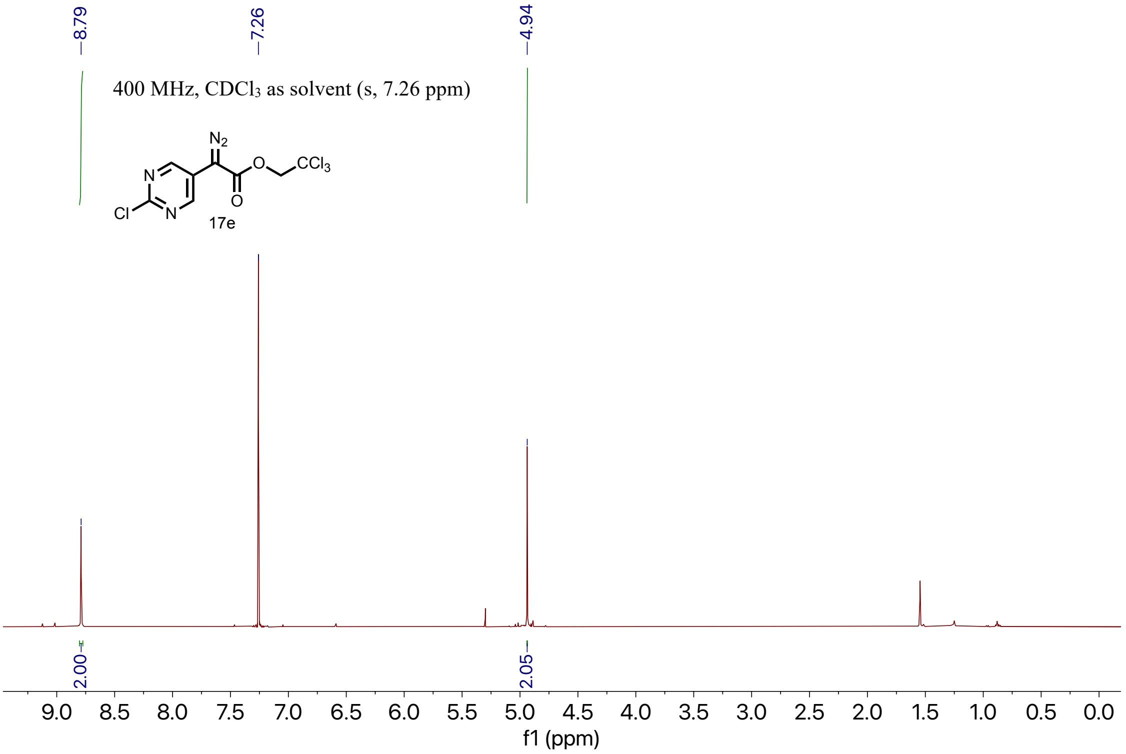
8.79
7.26
4.94
400 MHz, CDCl3 as solvent (s, 7.26 ppm)
N2
CCl3
O
N
Cl
17e
2.00
2.05
f1 (ppm)

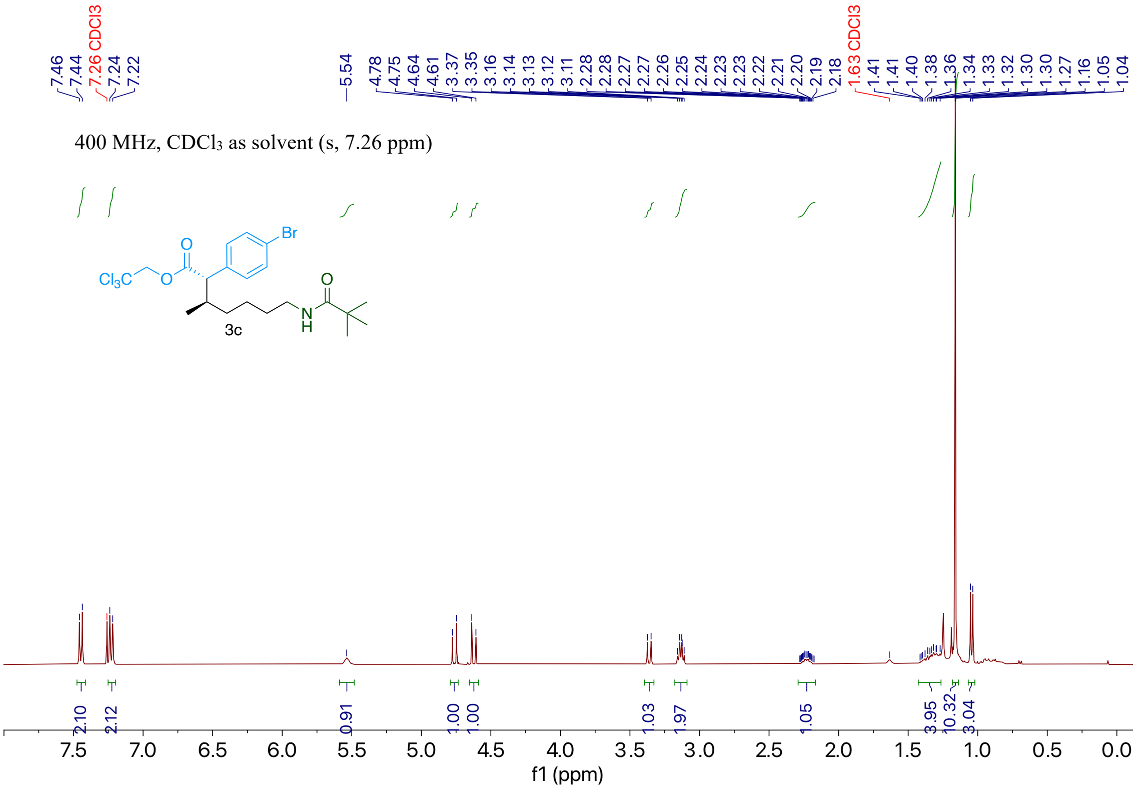
7.46
7.44
7.26 CDCl3
7.24
7.22
5.54
4.78
4.75
4.64
4.61
3.37
3.35
3.16
3.14
3.13
3.12
3.11
2.28
2.28
2.27
2.26
2.25
2.24
2.23
2.23
2.22
2.21
2.20
2.19
2.18
1.63 CDCl3
1.41
1.41
1.40
1.38
1.36
1.34
1.33
1.32
1.30
1.30
1.27
1.16
1.05
1.04
400 MHz, CDCl3 as solvent (s, 7.26 ppm)
Br
Cl3C
O
N
H
3c
2.10
2.12
0.91
1.00
1.00
1.03
1.97
1.05
3.95
10.32
3.04
f1 (ppm)

151 MHz, $CDCl_3$ as solvent (t, 77.16 ppm)

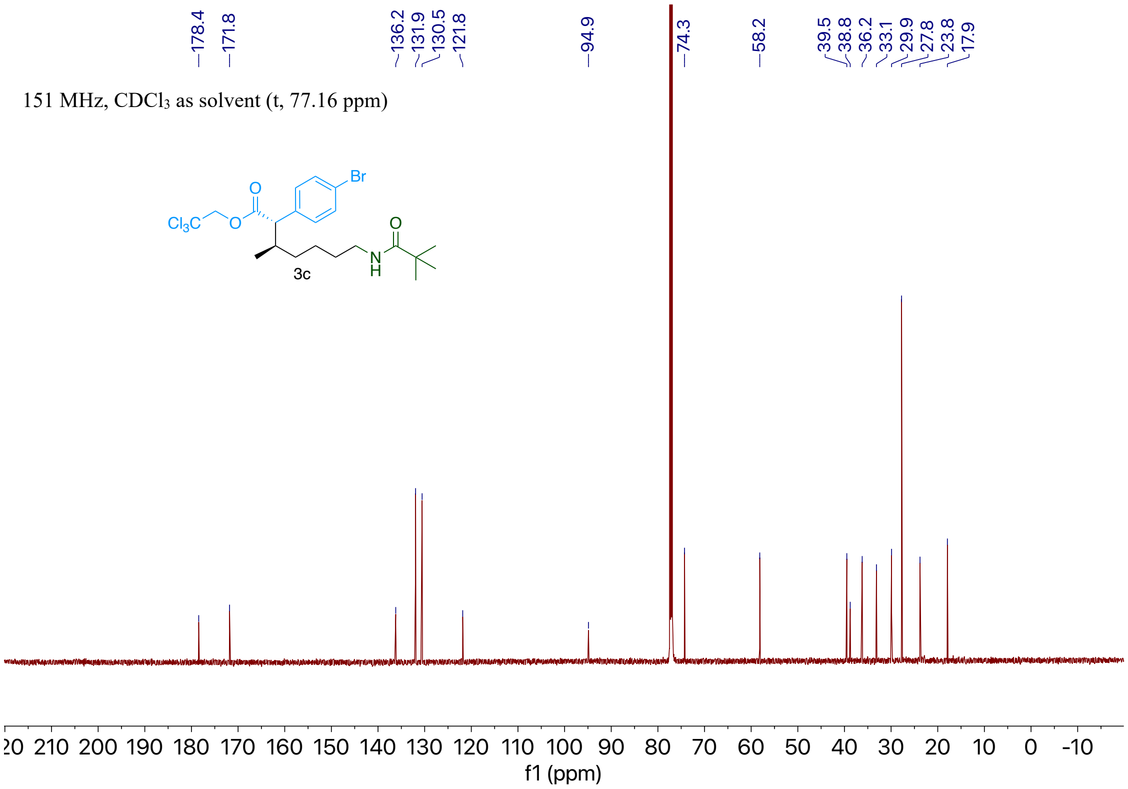

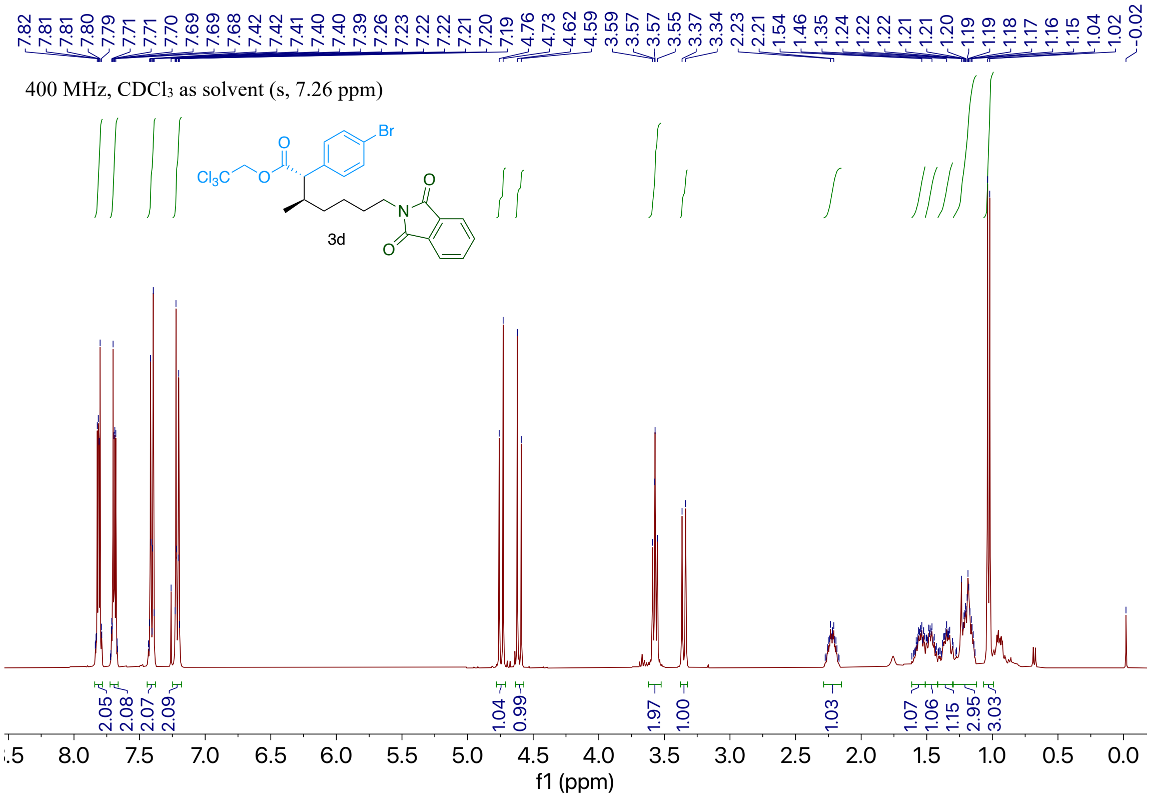

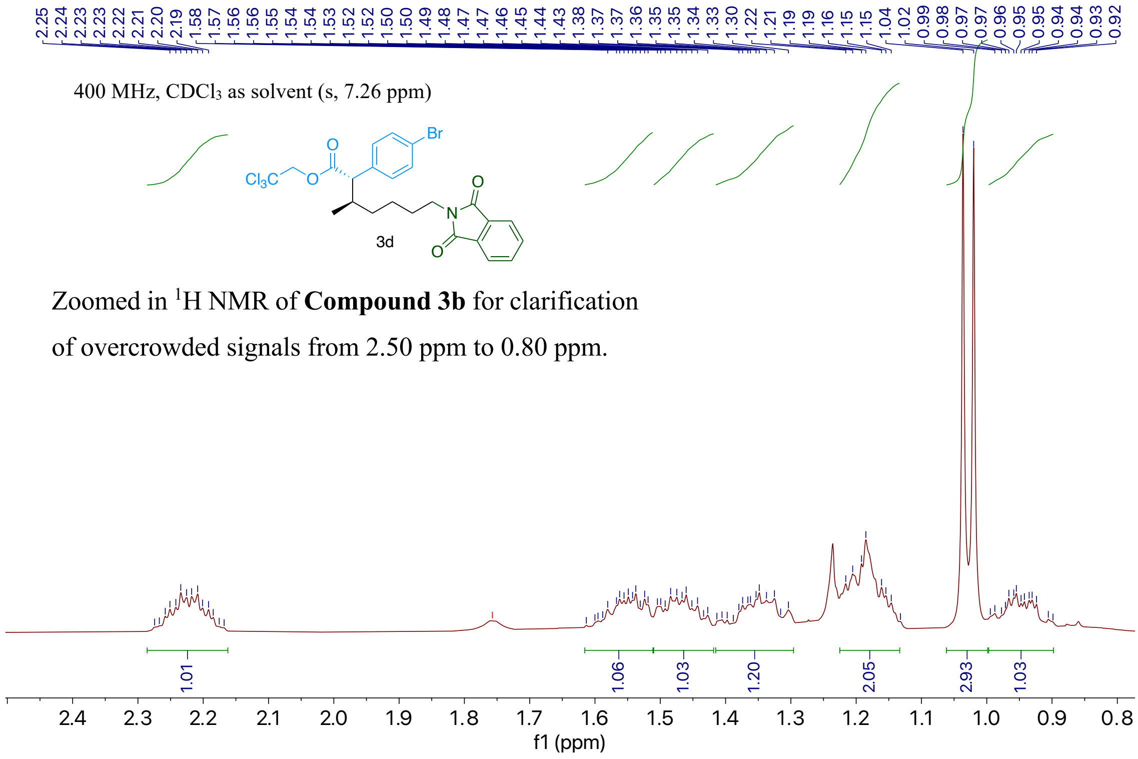

Zoomed in ^{1}H NMR of **Compound 3b** for clarification of overcrowded signals from 2.50 ppm to 0.80 ppm.

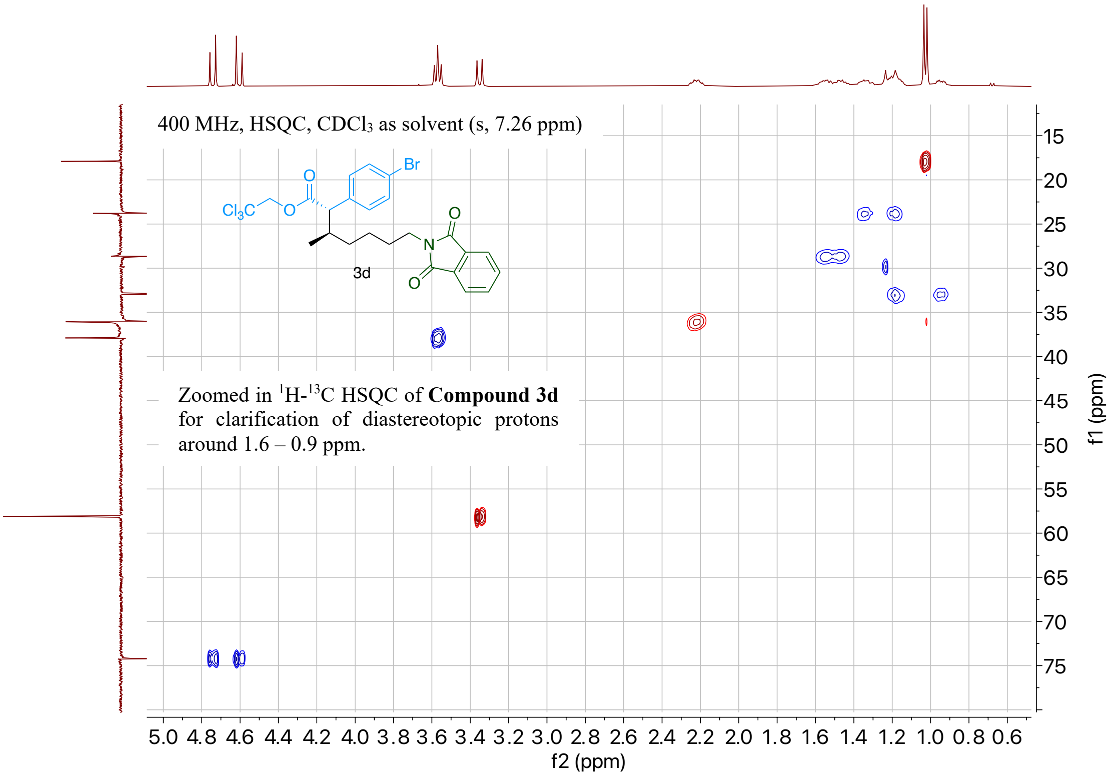
400 MHz, HSQC, CDCl3 as solvent (s, 7.26 ppm)
Br
O
Cl3C
O
N
O
O
3d
Zoomed in 1H-13C HSQC of Compound 3d for clarification of diastereotopic protons around 1.6 – 0.9 ppm.
5.0 4.8 4.6 4.4 4.2 4.0 3.8 3.6 3.4 3.2 3.0 2.8 2.6 2.4 2.2 2.0 1.8 1.6 1.4 1.2 1.0 0.8 0.6
f2 (ppm)
15 20 25 30 35 40 45 50 55 60 65 70 75
f1 (ppm)

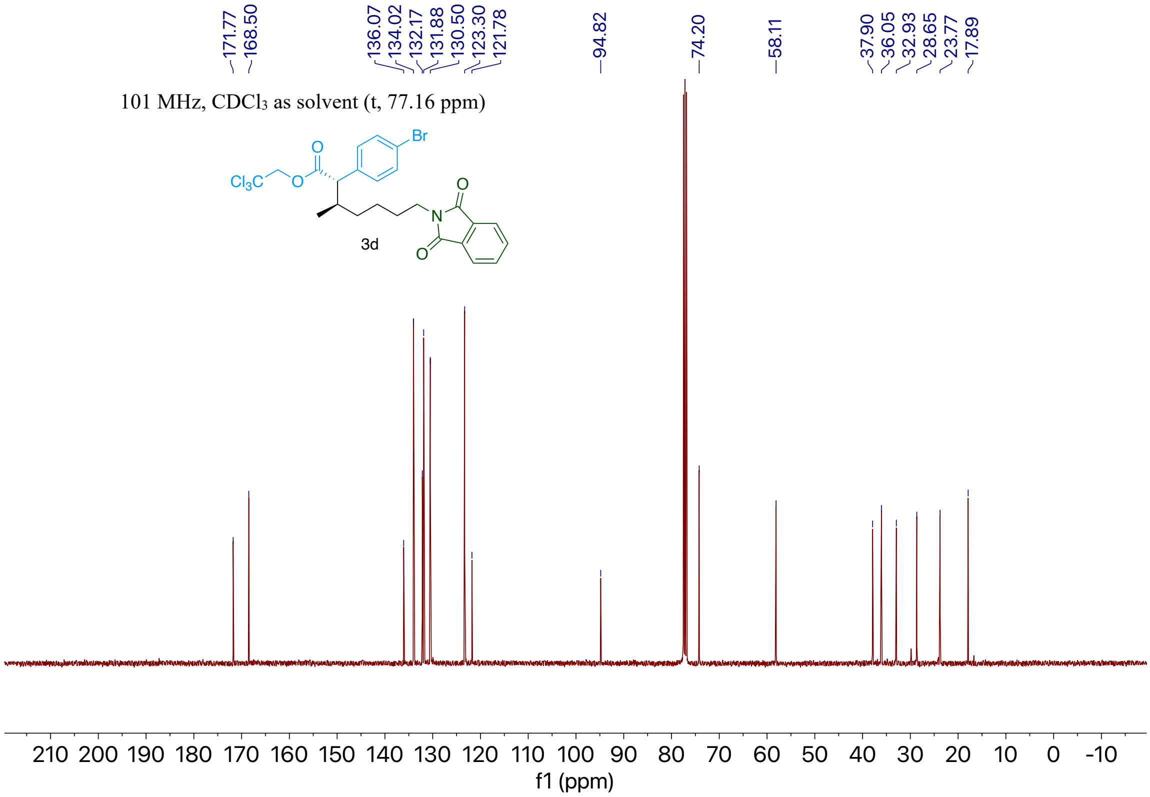
171.77
168.50
136.07
134.02
132.17
131.88
130.50
123.30
121.78
94.82
74.20
58.11
37.90
36.05
32.93
28.65
23.77
17.89
101 MHz, CDCl3 as solvent (t, 77.16 ppm)
Br
O
Cl3C
O
N
O
O
3d
210 200 190 180 170 160 150 140 130 120 110 100 90 80 70 60 50 40 30 20 10 0 -10
f1 (ppm)

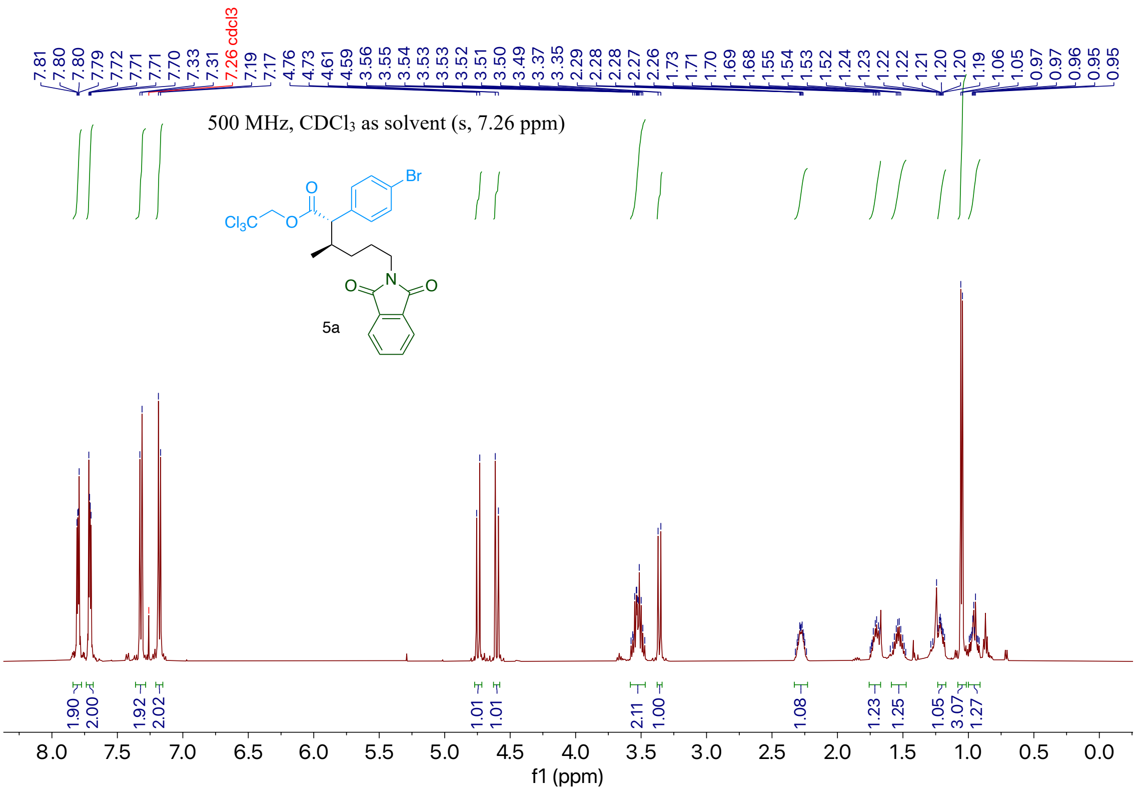
7.81 7.80 7.80 7.79 7.72 7.71 7.71 7.70 7.33 7.31 7.26 cdcl3 7.19 7.17 4.76 4.73 4.61 4.59 3.56 3.55 3.54 3.53 3.53 3.52 3.51 3.50 3.49 3.37 3.35 2.29 2.28 2.28 2.27 2.26 1.73 1.71 1.70 1.69 1.68 1.55 1.54 1.53 1.52 1.24 1.23 1.22 1.22 1.21 1.20 1.20 1.19 1.06 1.05 0.97 0.97 0.96 0.95 0.95
500 MHz, CDCl3 as solvent (s, 7.26 ppm)
Br
O
Cl3C
O
N
O
O
5a
1.90 2.00 1.92 2.02 1.01 1.01 2.11 1.00 1.08 1.23 1.25 1.05 3.07 1.27
8.0 7.5 7.0 6.5 6.0 5.5 5.0 4.5 4.0 3.5 3.0 2.5 2.0 1.5 1.0 0.5 0.0
f1 (ppm)

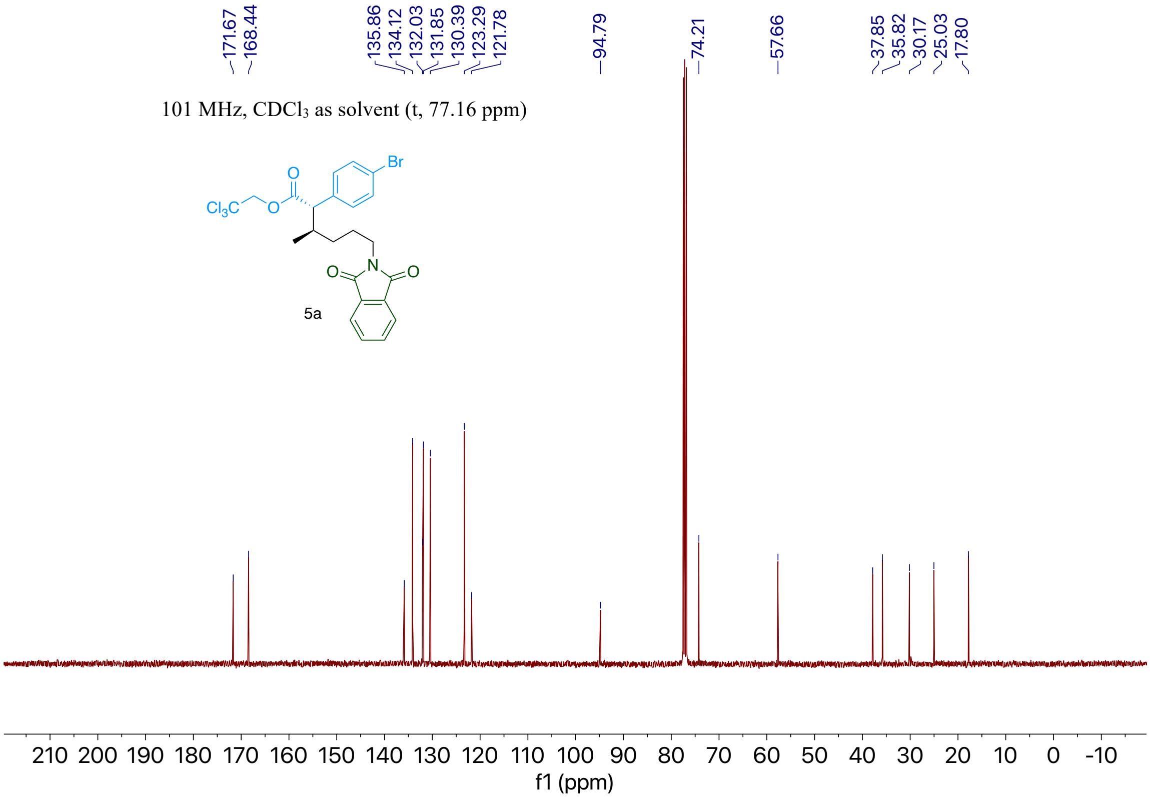
171.67 168.44 135.86 134.12 132.03 131.85 130.39 123.29 121.78 94.79 74.21 57.66 37.85 35.82 30.17 25.03 17.80
101 MHz, CDCl3 as solvent (t, 77.16 ppm)
Br
O
Cl3C
O
N
O
O
5a
210 200 190 180 170 160 150 140 130 120 110 100 90 80 70 60 50 40 30 20 10 0 -10
f1 (ppm)

400 MHz, CDCl3 as solvent (s, 7.26 ppm)

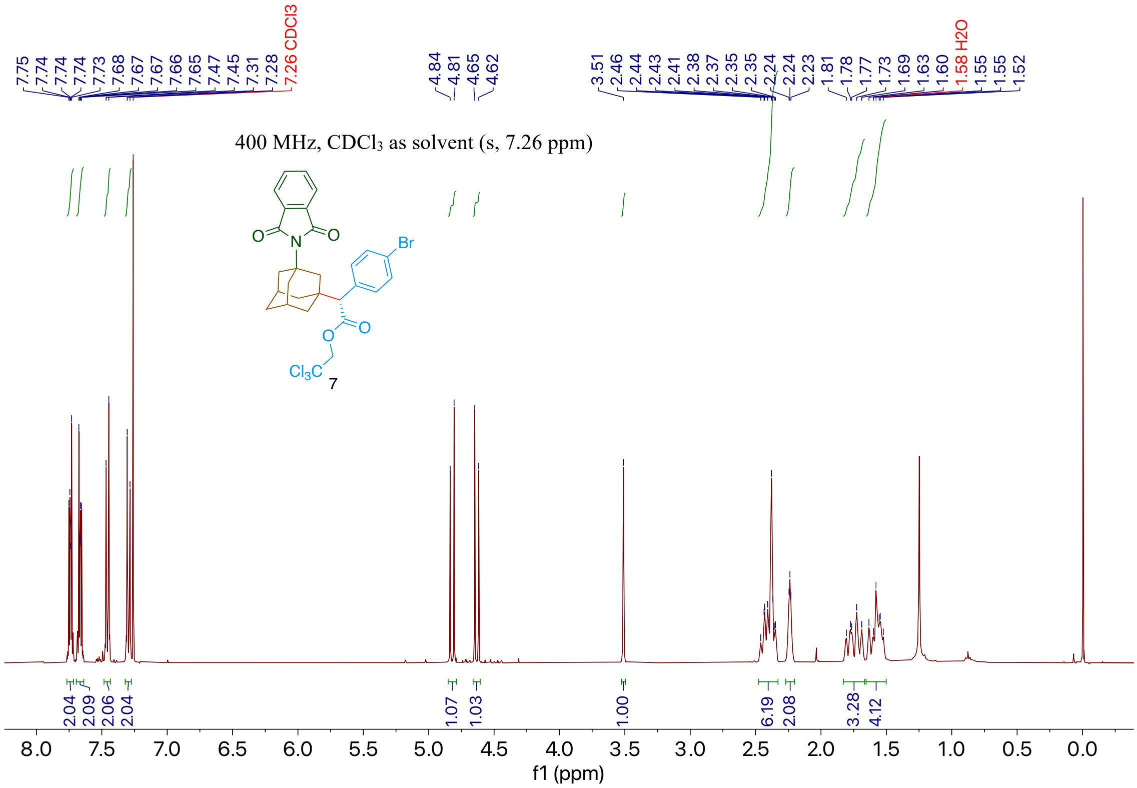

101 MHz, CDCl3 as solvent (t, 77.16 ppm)

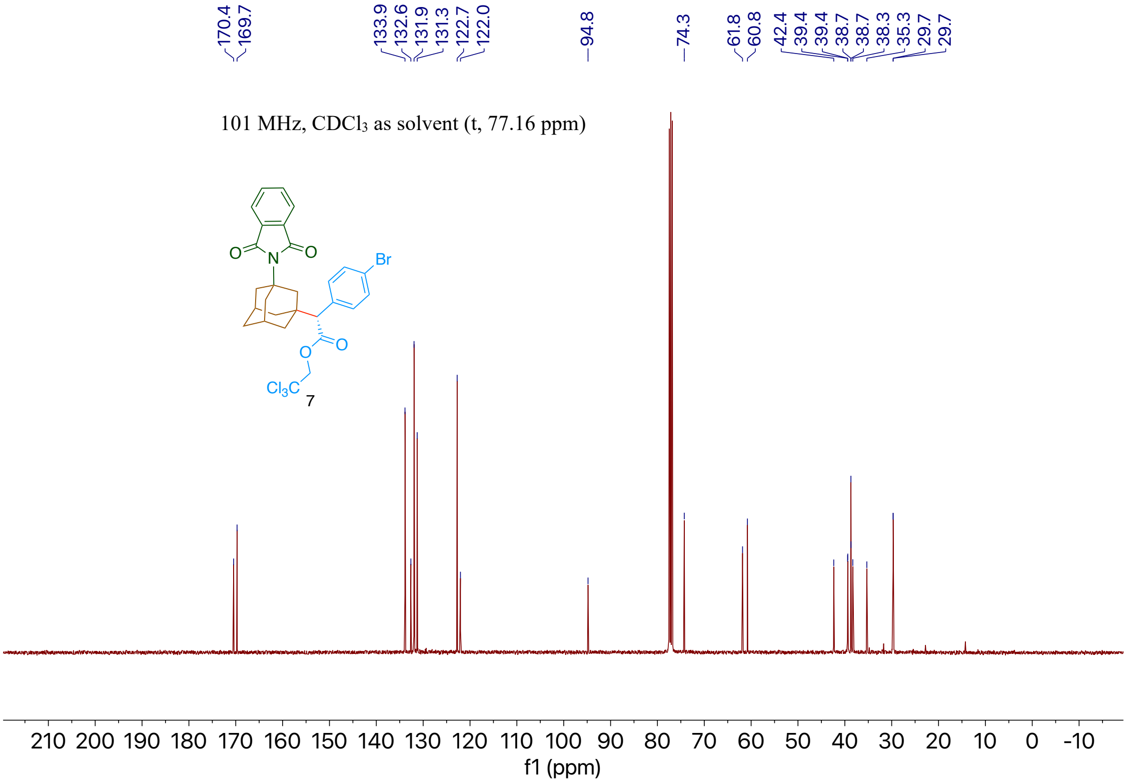

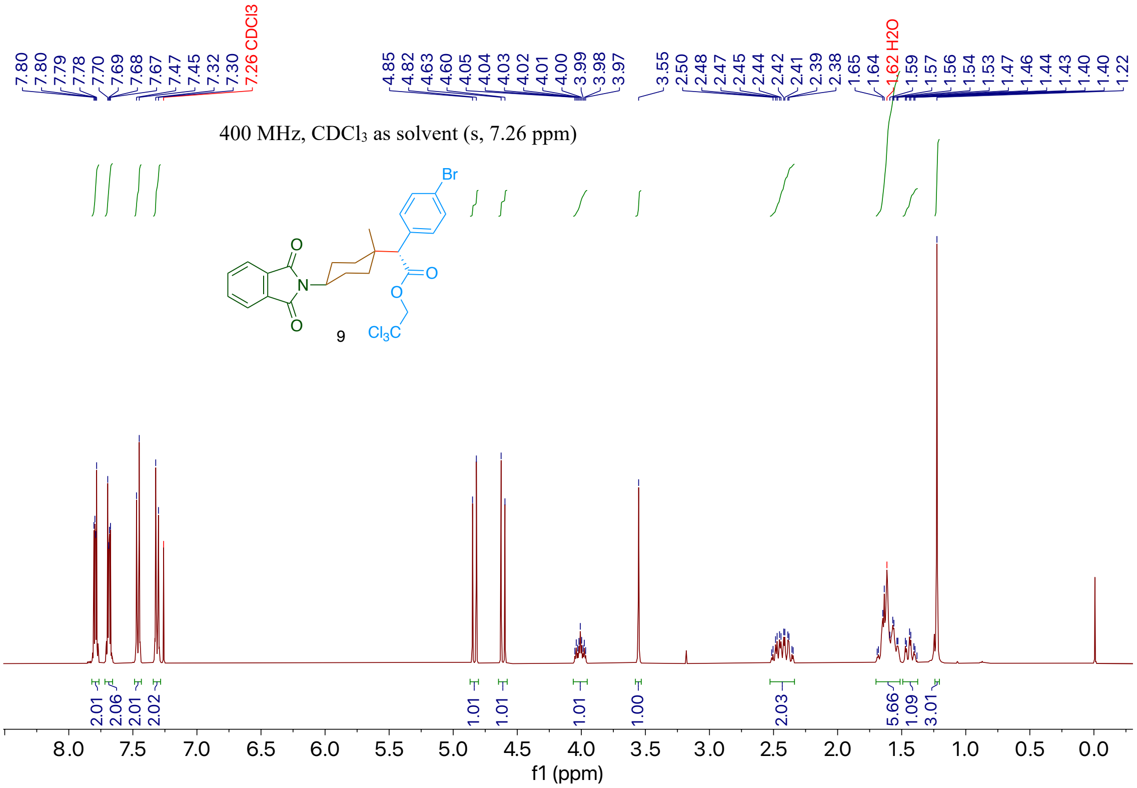
7.80
7.80
7.79
7.78
7.70
7.69
7.68
7.67
7.47
7.45
7.32
7.30
7.26 CDCl3
4.85
4.82
4.63
4.60
4.05
4.04
4.03
4.02
4.01
4.00
3.99
3.98
3.97
3.55
2.50
2.48
2.47
2.45
2.44
2.42
2.41
2.39
2.38
1.65
1.64
1.62 H2O
1.59
1.57
1.56
1.54
1.53
1.47
1.46
1.44
1.43
1.40
1.40
1.22
400 MHz, CDCl3 as solvent (s, 7.26 ppm)
Br
O
N
O
O
O
9
Cl3C
2.01
2.06
2.01
2.02
1.01
1.01
1.01
1.00
2.03
5.66
1.09
3.01
8.0 7.5 7.0 6.5 6.0 5.5 5.0 4.5 4.0 3.5 3.0 2.5 2.0 1.5 1.0 0.5 0.0
f1 (ppm)

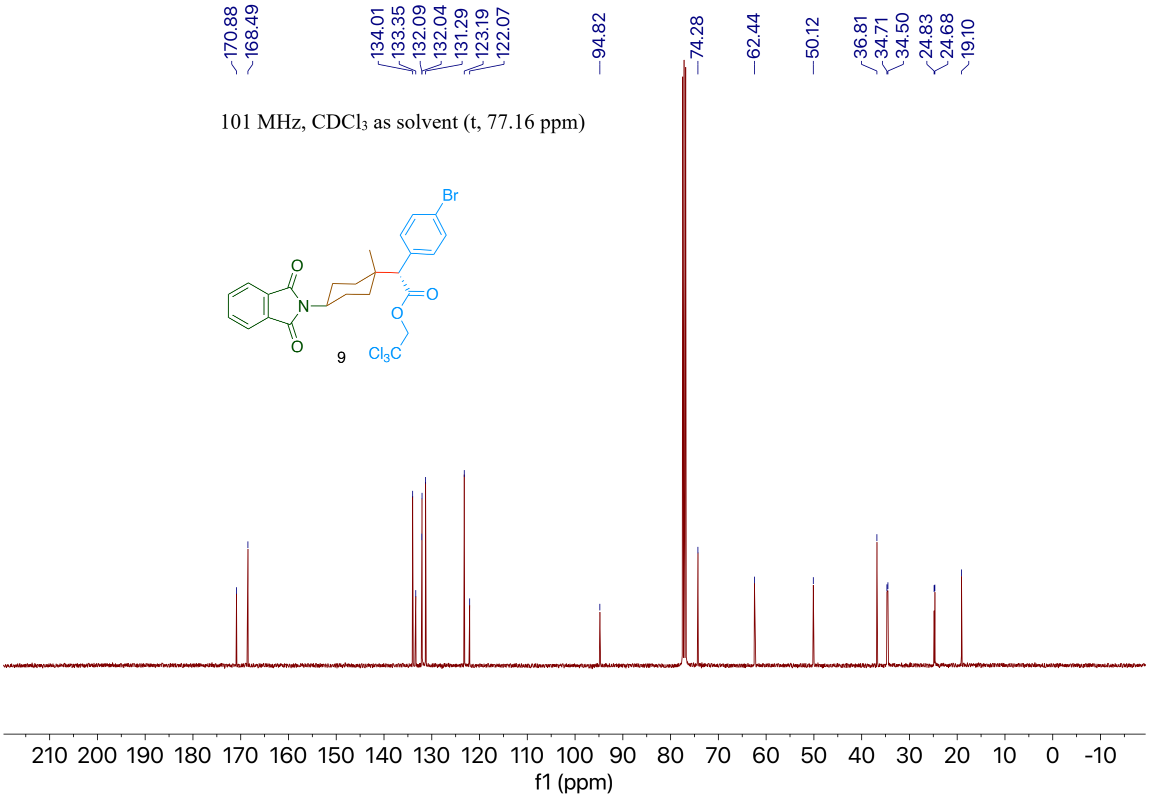
170.88
168.49
134.01
133.35
132.09
132.04
131.29
123.19
122.07
94.82
74.28
62.44
50.12
36.81
34.71
34.50
24.83
24.68
19.10
101 MHz, CDCl3 as solvent (t, 77.16 ppm)
Br
O
N
O
O
O
9
Cl3C
210 200 190 180 170 160 150 140 130 120 110 100 90 80 70 60 50 40 30 20 10 0 -10
f1 (ppm)

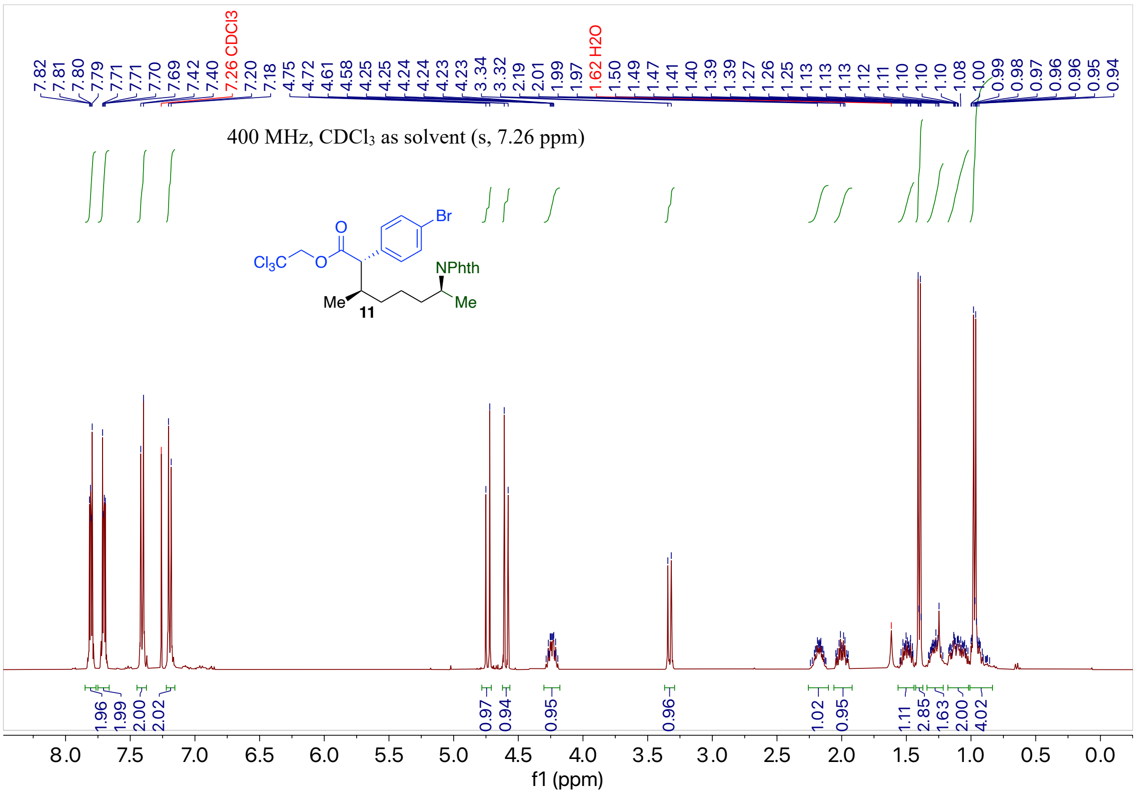
400 MHz, CDCl3 as solvent (s, 7.26 ppm)
7.26 CDCl3
1.62 H2O
Cl3C
O
Br
NPhth
Me
Me
11
f1 (ppm)

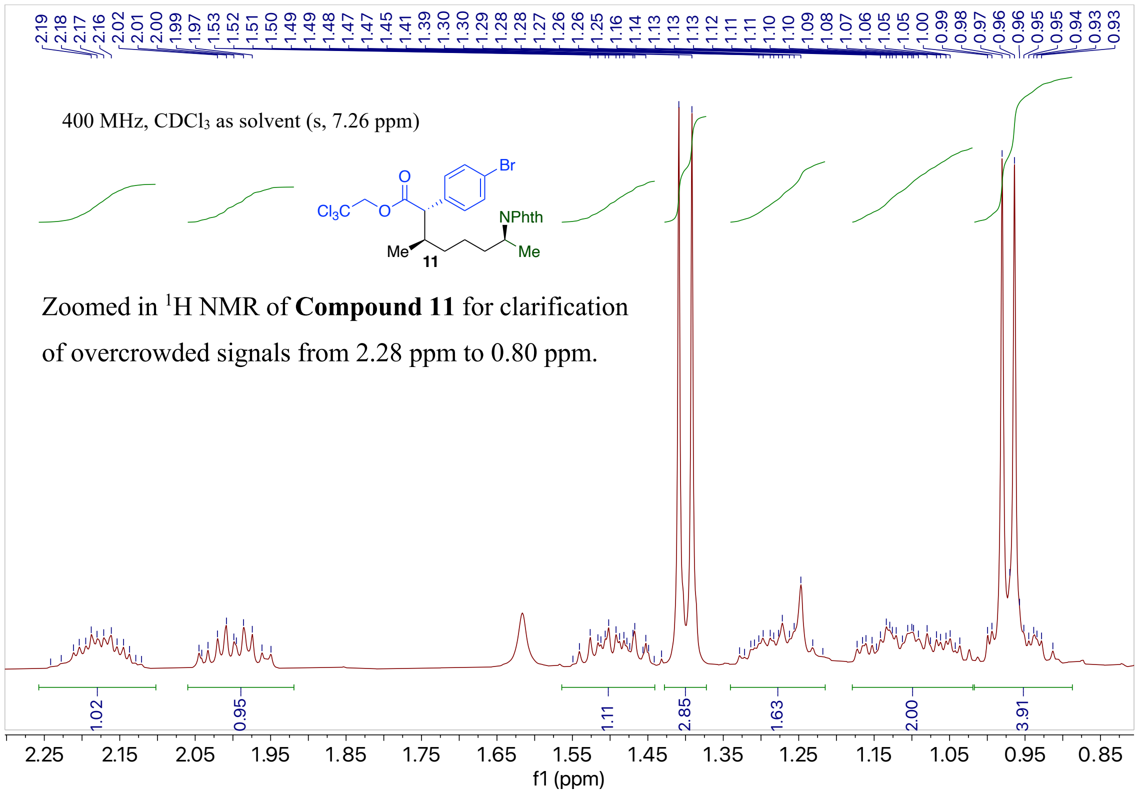
400 MHz, CDCl3 as solvent (s, 7.26 ppm)
Cl3C
O
Br
NPhth
Me
Me
11
Zoomed in 1H NMR of Compound 11 for clarification
of overcrowded signals from 2.28 ppm to 0.80 ppm.
f1 (ppm)

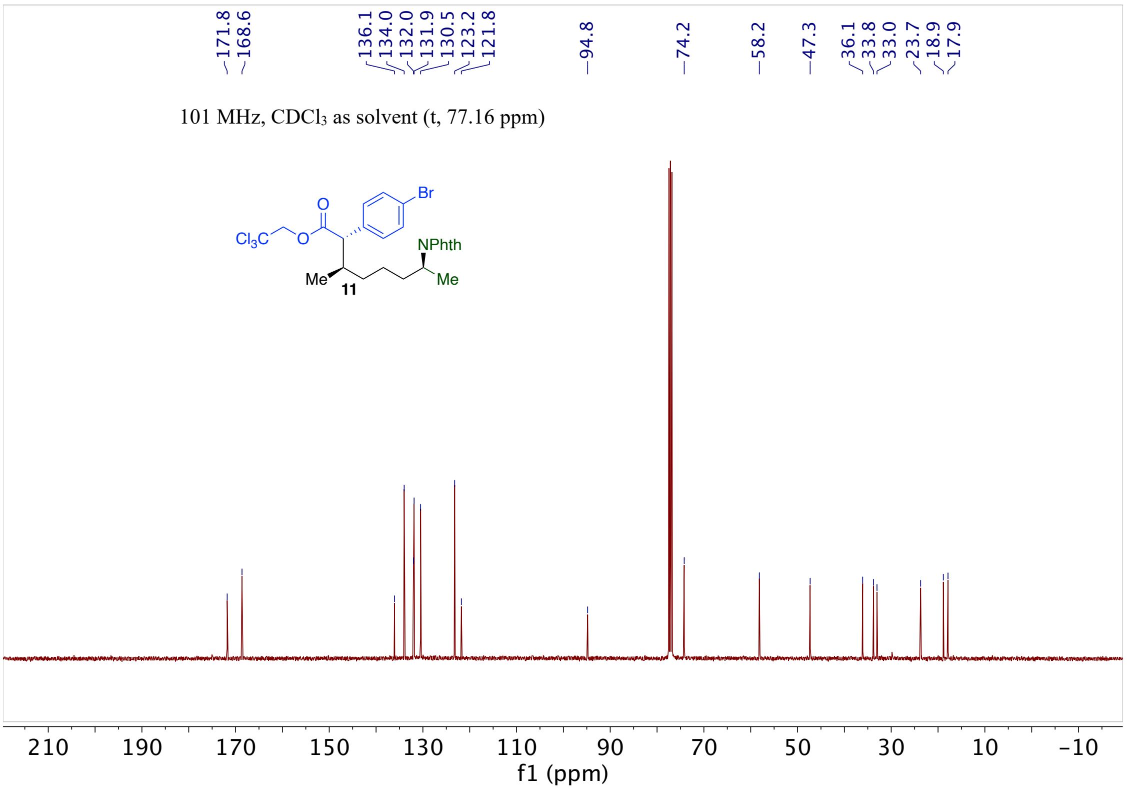
171.8
168.6
136.1
134.0
132.0
131.9
130.5
123.2
121.8
94.8
74.2
58.2
47.3
36.1
33.8
33.0
23.7
18.9
17.9
101 MHz, CDCl3 as solvent (t, 77.16 ppm)
Br
O
Cl3C
O
NPhth
Me
Me
11
210
190
170
150
130
110
90
70
50
30
10
-10
f1 (ppm)

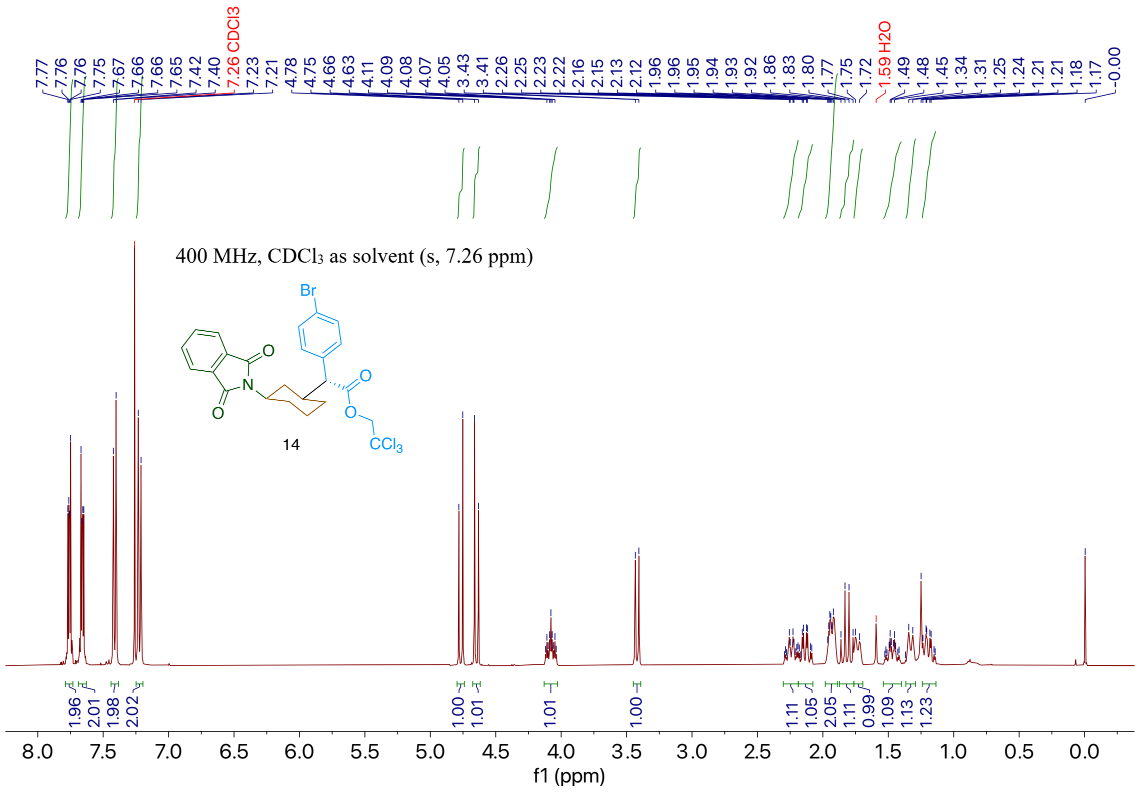
7.77
7.76
7.76
7.75
7.67
7.66
7.66
7.65
7.42
7.40
7.26 CDCl3
7.23
7.21
4.78
4.75
4.66
4.63
4.11
4.09
4.08
4.07
4.05
3.43
3.41
2.26
2.25
2.23
2.22
2.16
2.15
2.13
2.12
1.96
1.96
1.95
1.94
1.93
1.92
1.86
1.83
1.80
1.77
1.75
1.72
1.59 H2O
1.49
1.48
1.45
1.34
1.31
1.25
1.24
1.21
1.21
1.18
1.17
-0.00
400 MHz, CDCl3 as solvent (s, 7.26 ppm)
Br
O
N
O
O
O
CCl3
14
1.96
2.01
1.98
2.02
1.00
1.01
1.01
1.00
1.11
1.05
2.05
1.11
0.99
1.09
1.13
1.23
8.0
7.5
7.0
6.5
6.0
5.5
5.0
4.5
4.0
3.5
3.0
2.5
2.0
1.5
1.0
0.5
0.0
f1 (ppm)

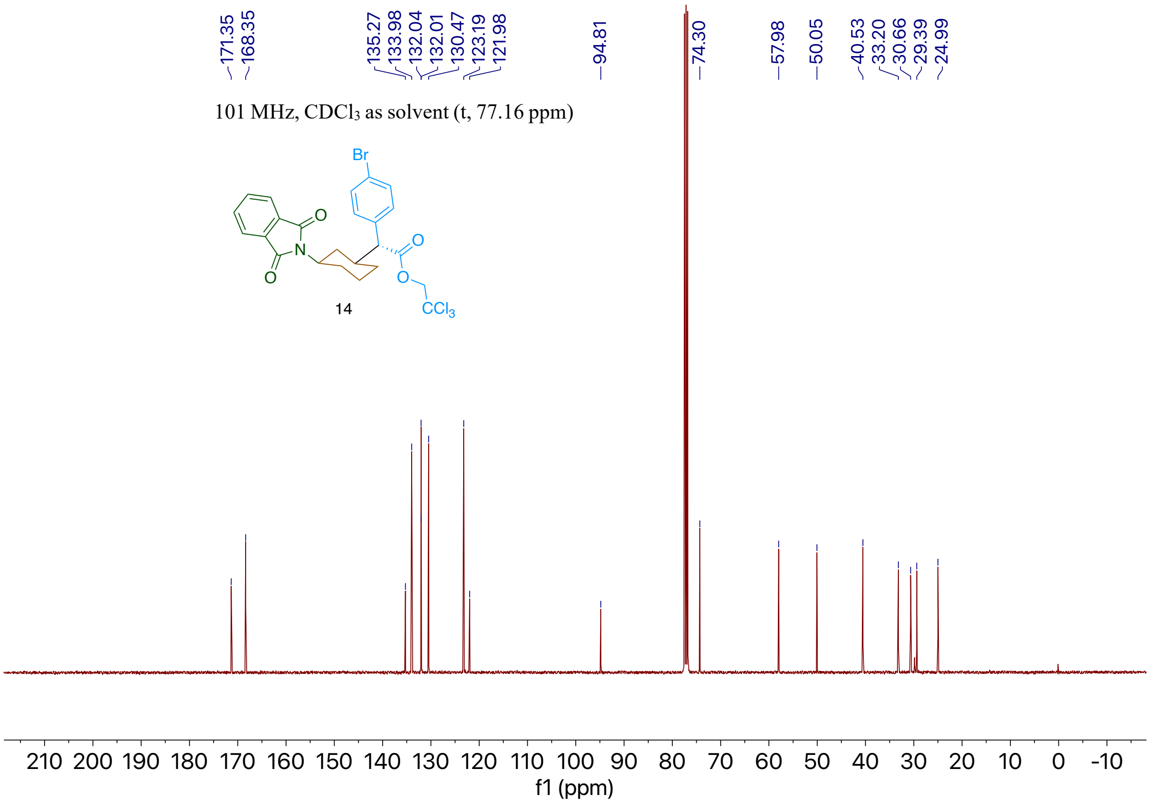
171.35
168.35
135.27
133.98
132.04
132.01
130.47
123.19
121.98
94.81
74.30
57.98
50.05
40.53
33.20
30.66
29.39
24.99
101 MHz, CDCl3 as solvent (t, 77.16 ppm)
Br
O
N
O
O
O
CCl3
14
210 200 190 180 170 160 150 140 130 120 110 100 90 80 70 60 50 40 30 20 10 0 -10
f1 (ppm)

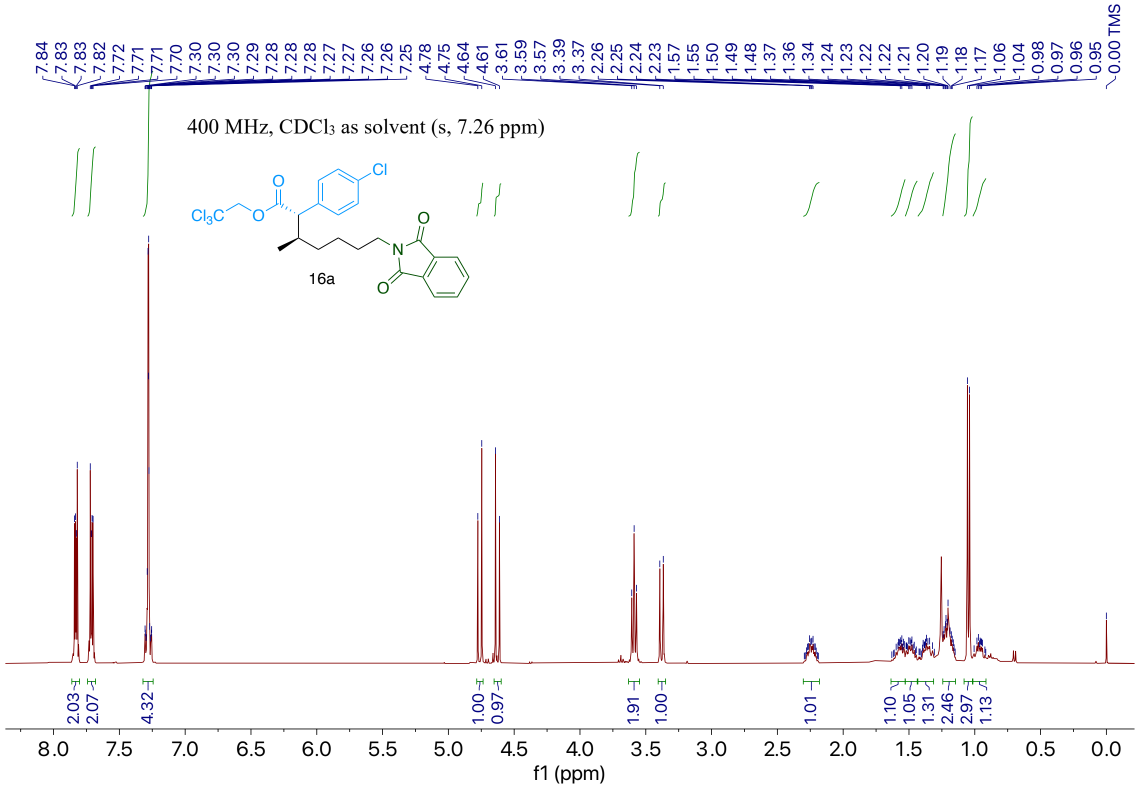
7.84
7.83
7.83
7.82
7.72
7.71
7.71
7.70
7.30
7.30
7.30
7.29
7.28
7.28
7.28
7.27
7.27
7.26
7.25
4.78
4.75
4.64
4.61
3.61
3.59
3.57
3.39
3.37
2.26
2.25
2.24
2.23
1.57
1.55
1.50
1.49
1.48
1.37
1.36
1.34
1.24
1.23
1.22
1.22
1.21
1.20
1.19
1.18
1.17
1.06
1.04
0.98
0.97
0.96
0.95
0.00 TMS
400 MHz, CDCl3 as solvent (s, 7.26 ppm)
Cl
O
Cl3C
O
N
O
O
16a
2.03
2.07
4.32
1.00
0.97
1.91
1.00
1.01
1.10
1.05
1.31
2.46
2.97
1.13
8.0 7.5 7.0 6.5 6.0 5.5 5.0 4.5 4.0 3.5 3.0 2.5 2.0 1.5 1.0 0.5 0.0
f1 (ppm)

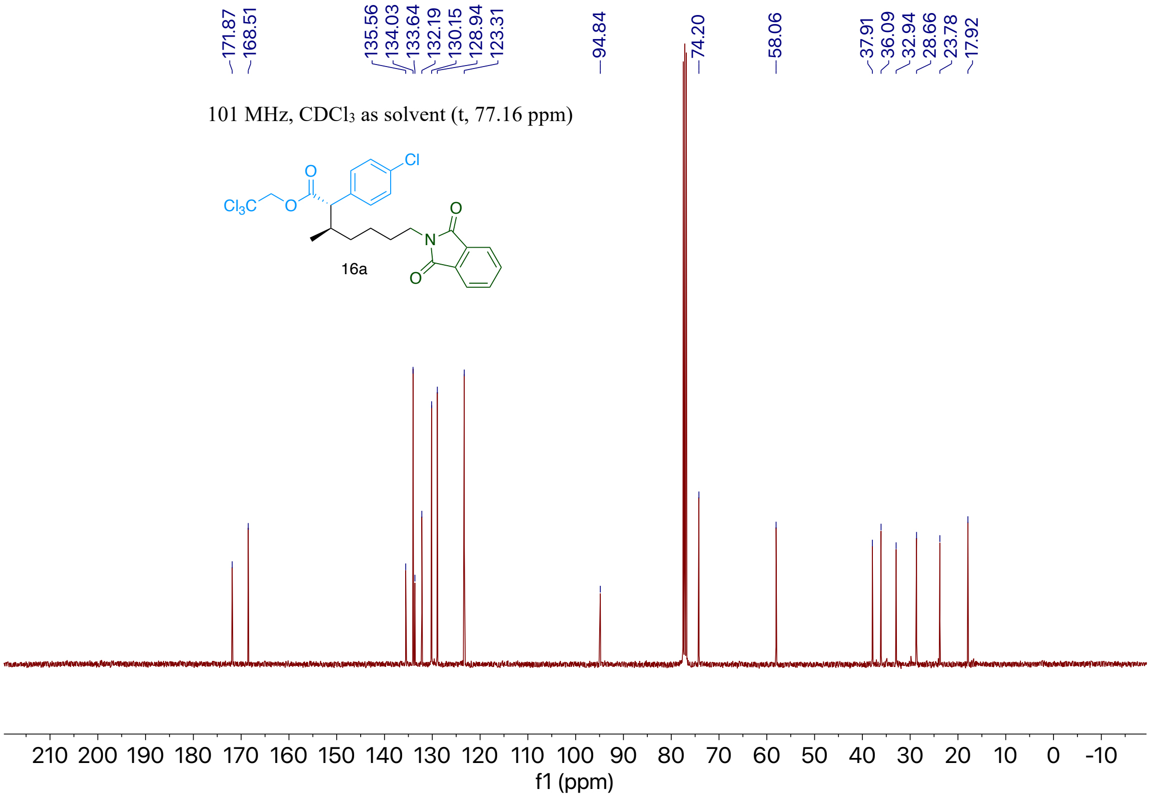
171.87
168.51
135.56
134.03
133.64
132.19
130.15
128.94
123.31
94.84
74.20
58.06
37.91
36.09
32.94
28.66
23.78
17.92
101 MHz, CDCl3 as solvent (t, 77.16 ppm)
16a
210 200 190 180 170 160 150 140 130 120 110 100 90 80 70 60 50 40 30 20 10 0 -10
f1 (ppm)

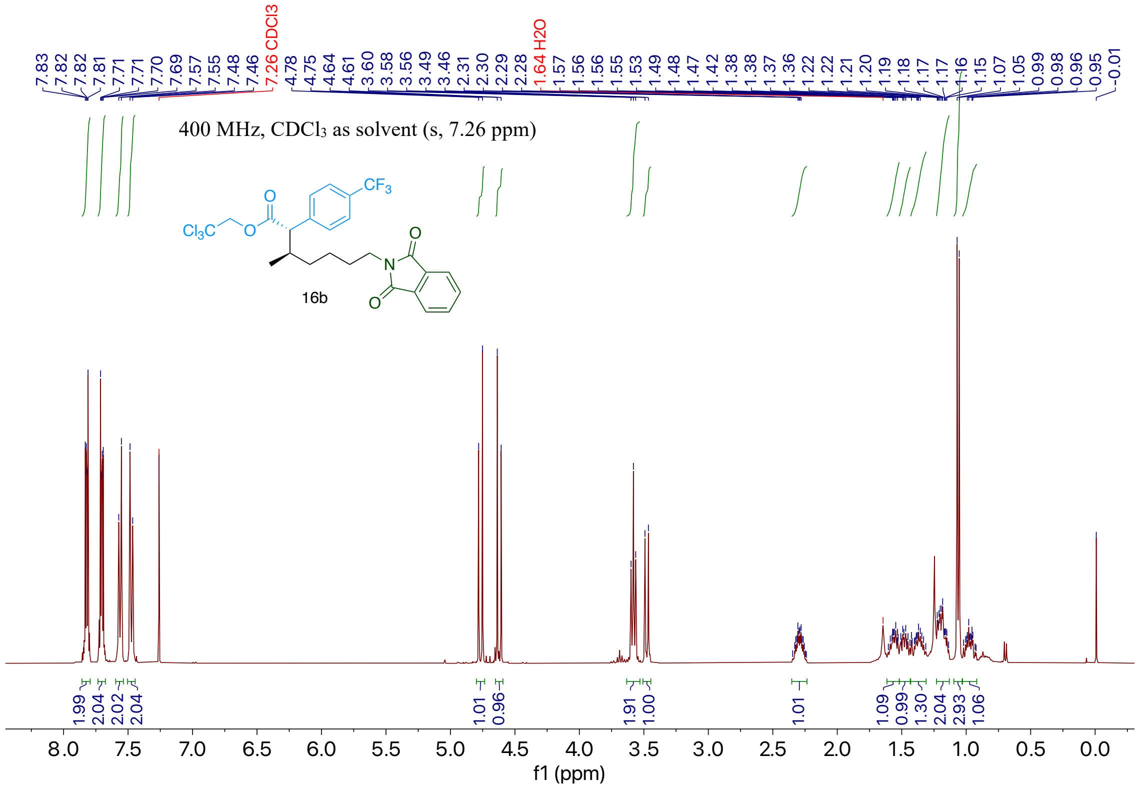
7.83
7.82
7.82
7.81
7.71
7.71
7.70
7.69
7.57
7.55
7.48
7.46
7.26 CDCl3
4.78
4.75
4.64
4.61
3.60
3.58
3.56
3.49
3.46
2.31
2.30
2.29
2.28
1.64 H2O
1.57
1.56
1.56
1.55
1.53
1.49
1.48
1.47
1.42
1.38
1.38
1.37
1.36
1.22
1.22
1.21
1.20
1.19
1.18
1.17
1.17
1.16
1.15
1.07
1.05
0.99
0.98
0.96
0.95
-0.01
400 MHz, CDCl3 as solvent (s, 7.26 ppm)
16b
1.99
2.04
2.02
2.04
1.01
0.96
1.91
1.00
1.01
1.09
0.99
1.30
2.04
2.93
1.06
8.0 7.5 7.0 6.5 6.0 5.5 5.0 4.5 4.0 3.5 3.0 2.5 2.0 1.5 1.0 0.5 0.0
f1 (ppm)

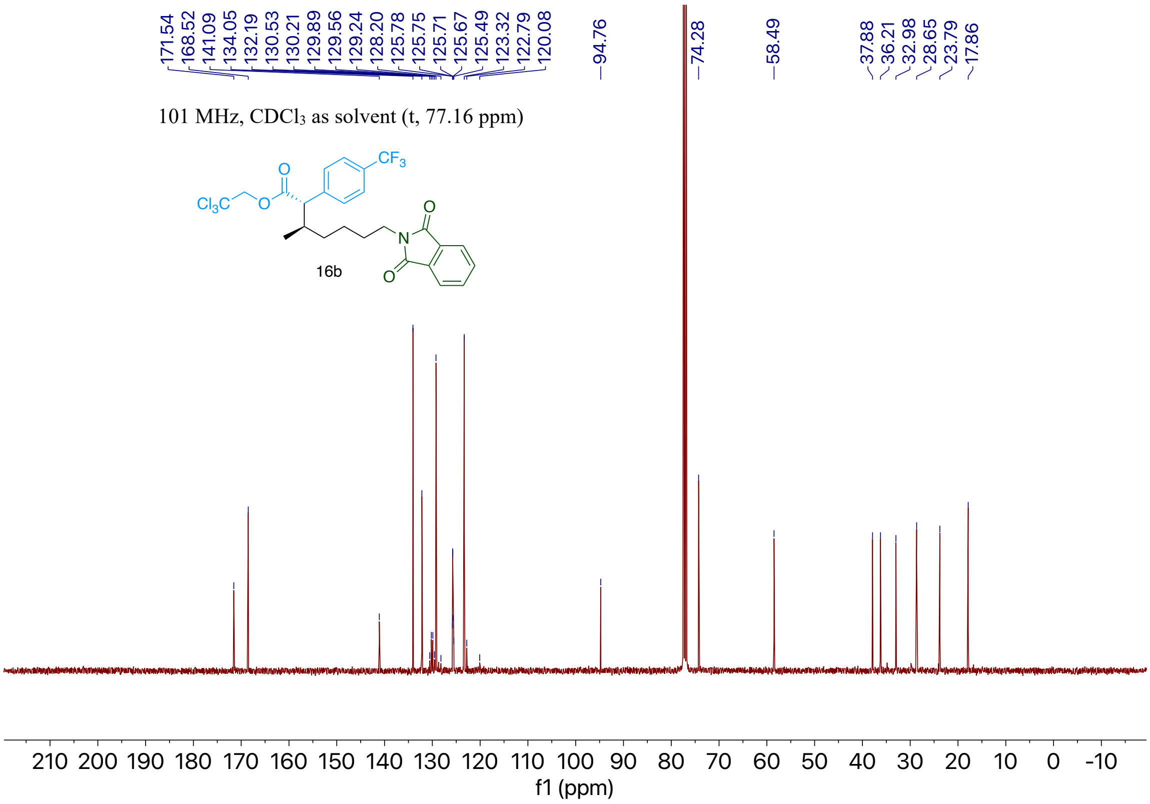
171.54
168.52
141.09
134.05
132.19
130.53
130.21
129.89
129.56
129.24
128.20
125.78
125.75
125.71
125.67
125.49
123.32
122.79
120.08
94.76
74.28
58.49
37.88
36.21
32.98
28.65
23.79
17.86
101 MHz, CDCl3 as solvent (t, 77.16 ppm)
CF3
O
Cl3C
O
O
N
O
16b
210 200 190 180 170 160 150 140 130 120 110 100 90 80 70 60 50 40 30 20 10 0 -10
f1 (ppm)

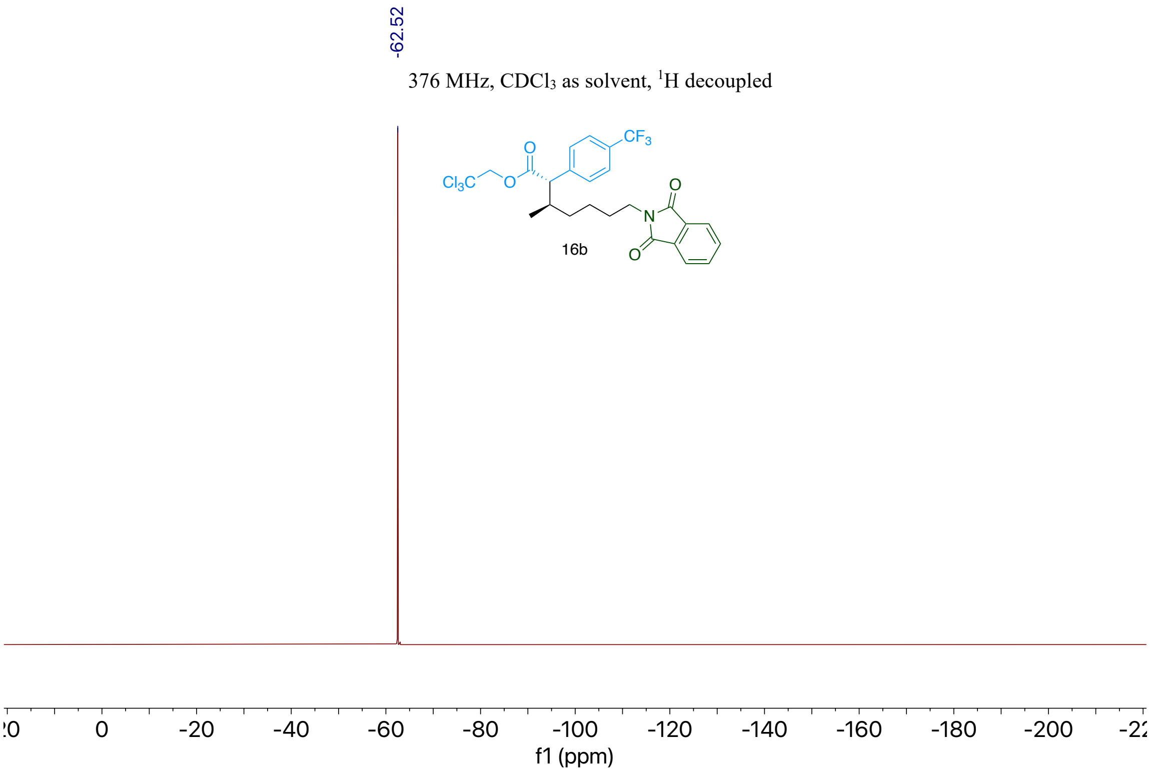
-62.52
376 MHz, CDCl3 as solvent, 1H decoupled
CF3
O
Cl3C
O
O
N
O
16b
0 -20 -40 -60 -80 -100 -120 -140 -160 -180 -200
f1 (ppm)

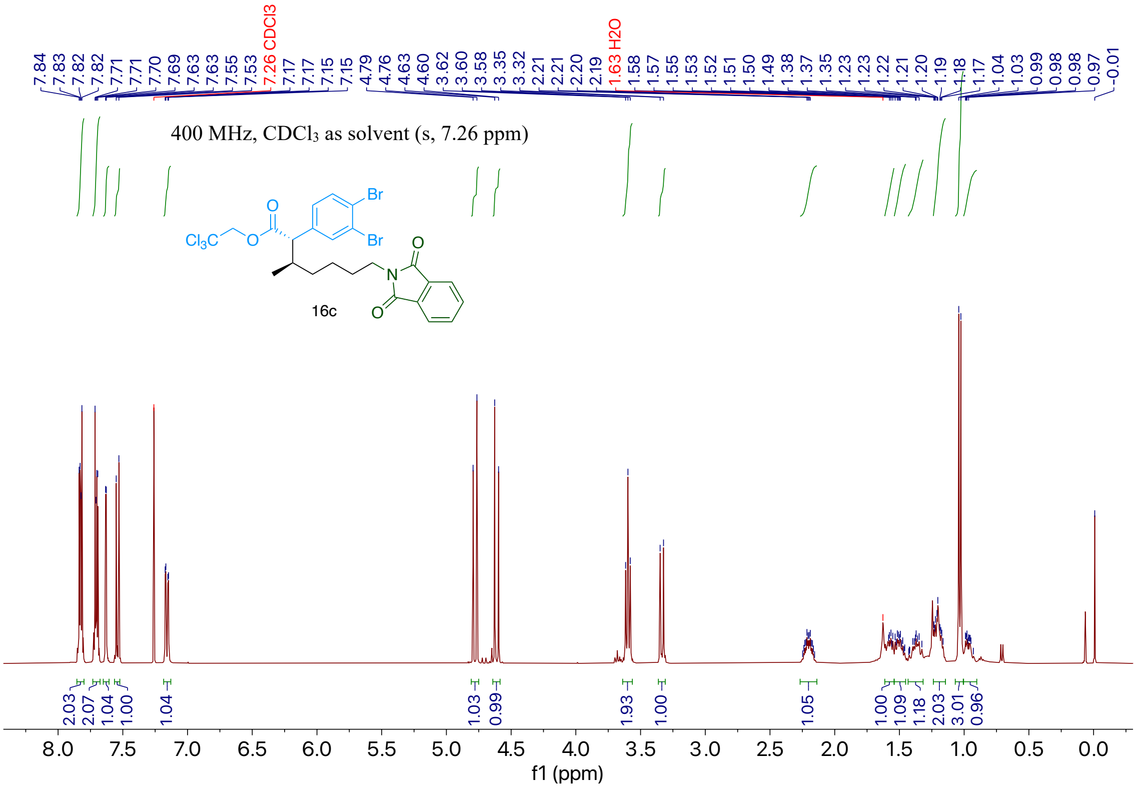

7.84 7.83 7.82 7.82 7.71 7.71 7.70 7.69 7.63 7.63 7.55 7.53 7.26 CDCl3 7.17 7.17 7.15 7.15 4.79 4.76 4.63 4.60 3.62 3.60 3.58 3.35 3.32 2.21 2.21 2.20 2.19 1.63 H2O 1.58 1.57 1.55 1.53 1.52 1.51 1.50 1.49 1.38 1.37 1.35 1.23 1.23 1.22 1.21 1.20 1.19 1.18 1.17 1.04 1.03 0.99 0.98 0.98 0.97 -0.01
400 MHz, CDCl3 as solvent (s, 7.26 ppm)
Br
Br
O
Cl3C
O
N
O
O
16c
2.03 2.07 1.04 1.00 1.04 1.03 0.99 1.93 1.00 1.05 1.00 1.09 1.18 2.03 3.01 0.96
8.0 7.5 7.0 6.5 6.0 5.5 5.0 4.5 4.0 3.5 3.0 2.5 2.0 1.5 1.0 0.5 0.0
f1 (ppm)

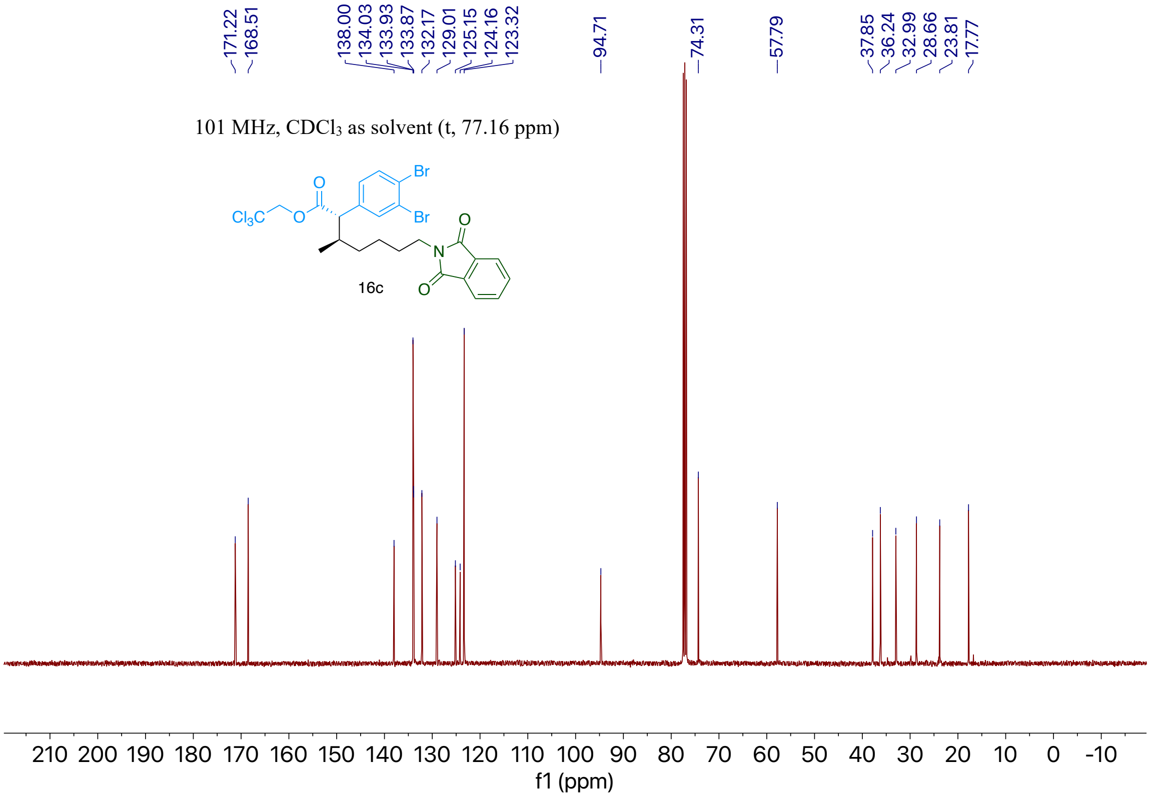

171.22 168.51 138.00 134.03 133.93 133.87 132.17 129.01 125.15 124.16 123.32 94.71 74.31 57.79 37.85 36.24 32.99 28.66 23.81 17.77
101 MHz, CDCl3 as solvent (t, 77.16 ppm)
Br
Br
O
Cl3C
O
N
O
O
16c
210 200 190 180 170 160 150 140 130 120 110 100 90 80 70 60 50 40 30 20 10 0 -10
f1 (ppm)

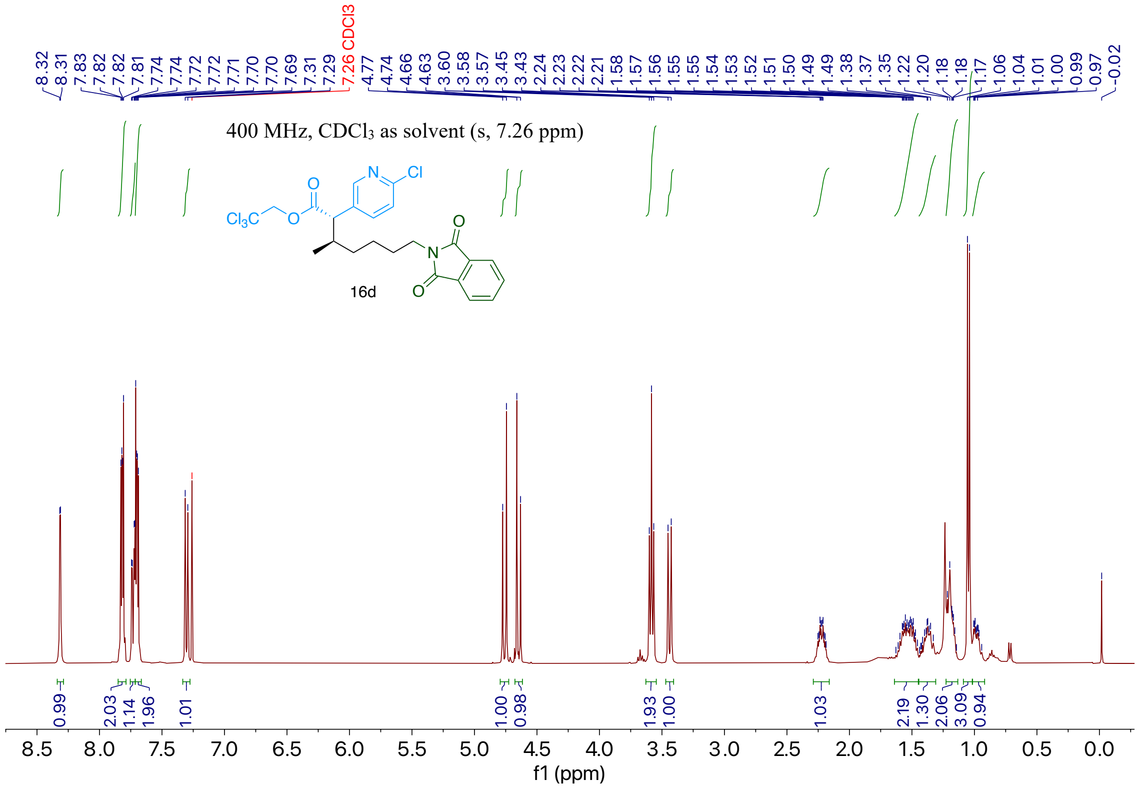

8.32
8.31
7.83
7.82
7.82
7.81
7.74
7.74
7.72
7.72
7.71
7.70
7.70
7.69
7.31
7.29
7.26 CDCl3
4.77
4.74
4.66
4.63
3.60
3.58
3.57
3.45
3.43
2.24
2.23
2.22
2.21
1.58
1.57
1.56
1.55
1.55
1.54
1.53
1.52
1.51
1.50
1.49
1.49
1.38
1.37
1.35
1.22
1.20
1.18
1.18
1.17
1.06
1.04
1.01
1.00
0.99
0.97
-0.02
400 MHz, CDCl3 as solvent (s, 7.26 ppm)
Cl3C
O
O
N
Cl
N
O
O
16d
0.99
2.03
1.14
1.96
1.01
1.00
0.98
1.93
1.00
1.03
2.19
1.30
2.06
3.09
0.94
8.5 8.0 7.5 7.0 6.5 6.0 5.5 5.0 4.5 4.0 3.5 3.0 2.5 2.0 1.5 1.0 0.5 0.0
f1 (ppm)

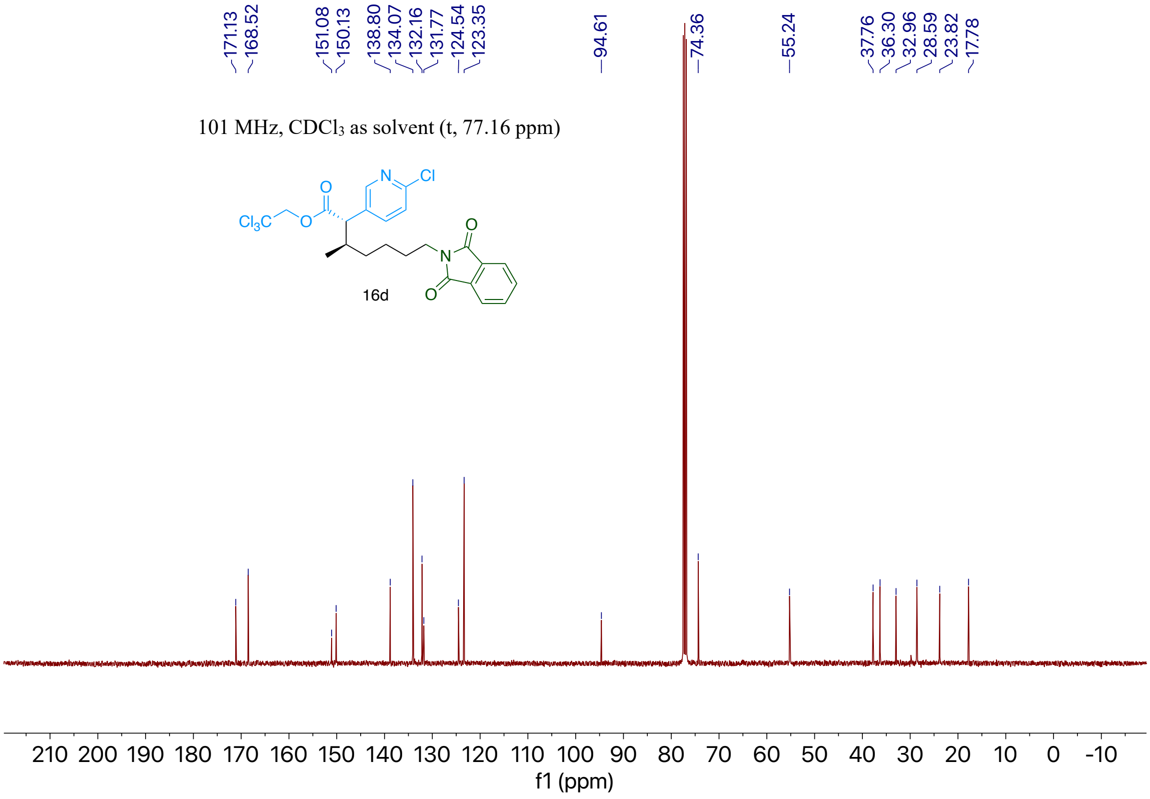

171.13
168.52
151.08
150.13
138.80
134.07
132.16
131.77
124.54
123.35
94.61
74.36
55.24
37.76
36.30
32.96
28.59
23.82
17.78
101 MHz, CDCl3 as solvent (t, 77.16 ppm)
Cl3C
O
O
N
Cl
N
O
O
16d
210 200 190 180 170 160 150 140 130 120 110 100 90 80 70 60 50 40 30 20 10 0 -10
f1 (ppm)

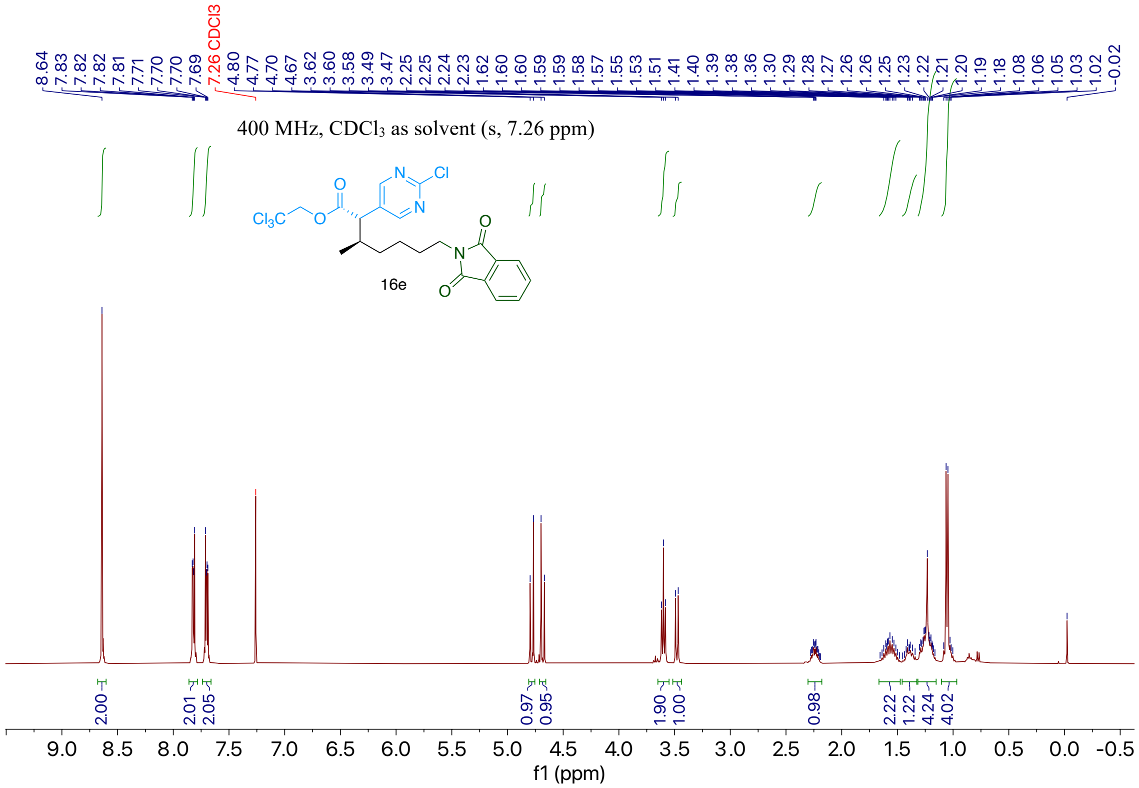
8.64 7.83 7.82 7.82 7.81 7.71 7.70 7.70 7.69 7.26 CDCl3 4.80 4.77 4.70 4.67 3.62 3.60 3.58 3.49 3.47 2.25 2.25 2.24 2.23 1.62 1.60 1.60 1.59 1.59 1.58 1.57 1.55 1.53 1.51 1.41 1.40 1.39 1.38 1.36 1.30 1.29 1.28 1.27 1.26 1.26 1.25 1.23 1.22 1.21 1.20 1.19 1.18 1.08 1.06 1.05 1.03 1.02 -0.02
400 MHz, CDCl3 as solvent (s, 7.26 ppm)
16e
2.00 2.01 2.05 0.97 0.95 1.90 1.00 0.98 2.22 1.22 4.24 4.02
f1 (ppm)

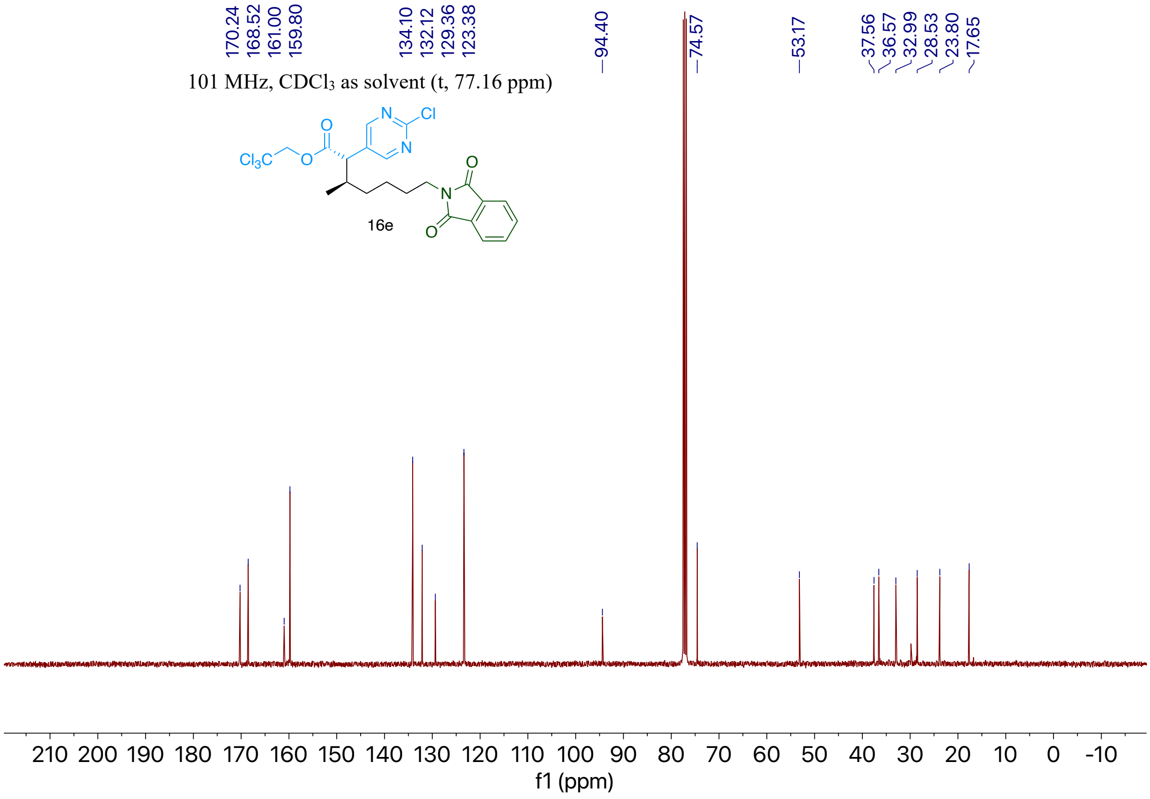
170.24 168.52 161.00 159.80 134.10 132.12 129.36 123.38 94.40 74.57 53.17 37.56 36.57 32.99 28.53 23.80 17.65
101 MHz, CDCl3 as solvent (t, 77.16 ppm)
16e
f1 (ppm)

6. *Crude NMR for determination of regioselectivity and diastereoselectivity*

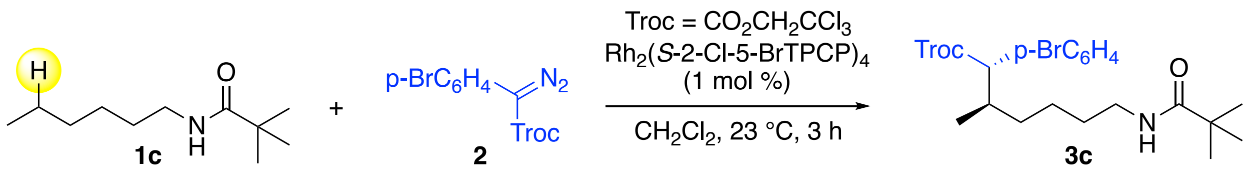

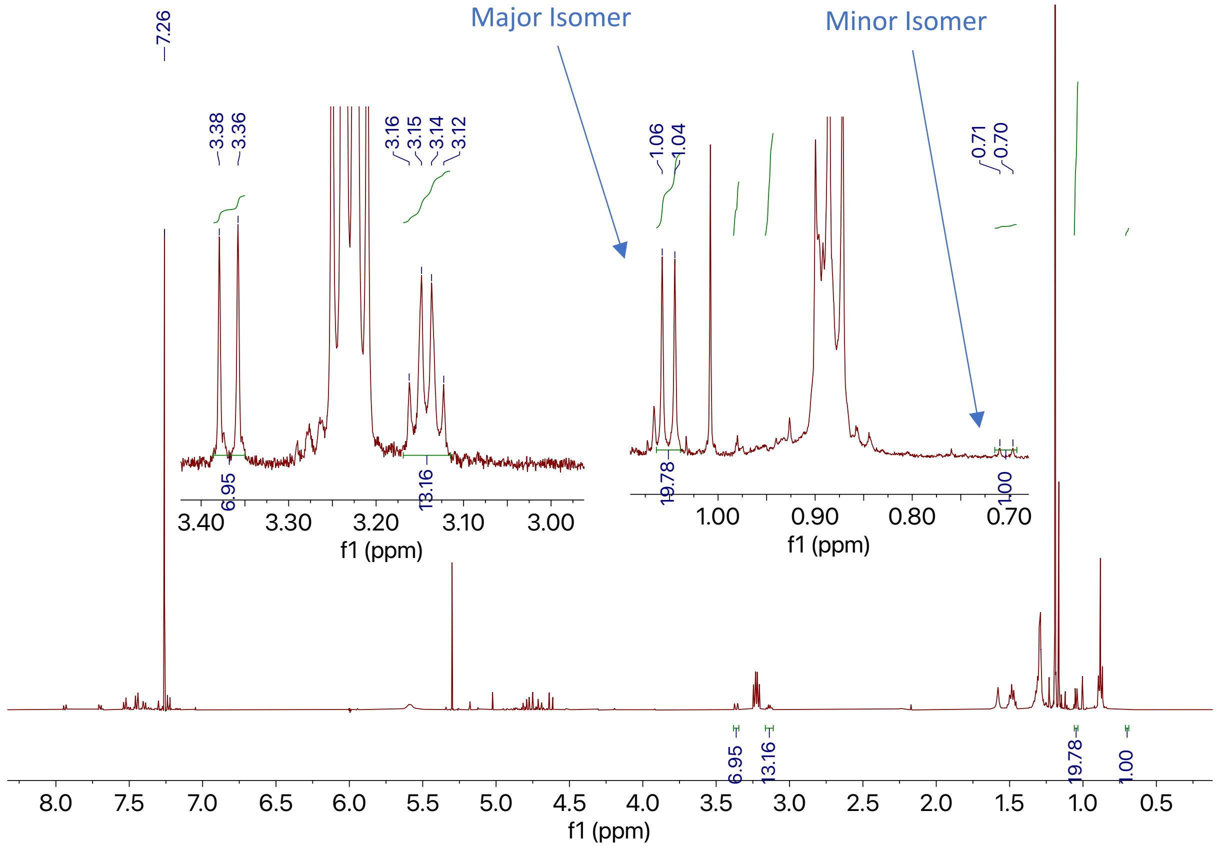

The C2 selectivity is assigned based on the doublet at 3.38 (benzylic H) and the methyl doublets at 1.05 (major) and 0.70 (minor) as previously discussed for related systems[15].

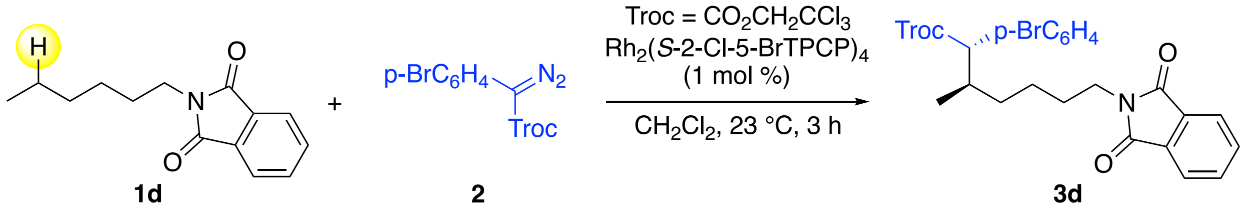

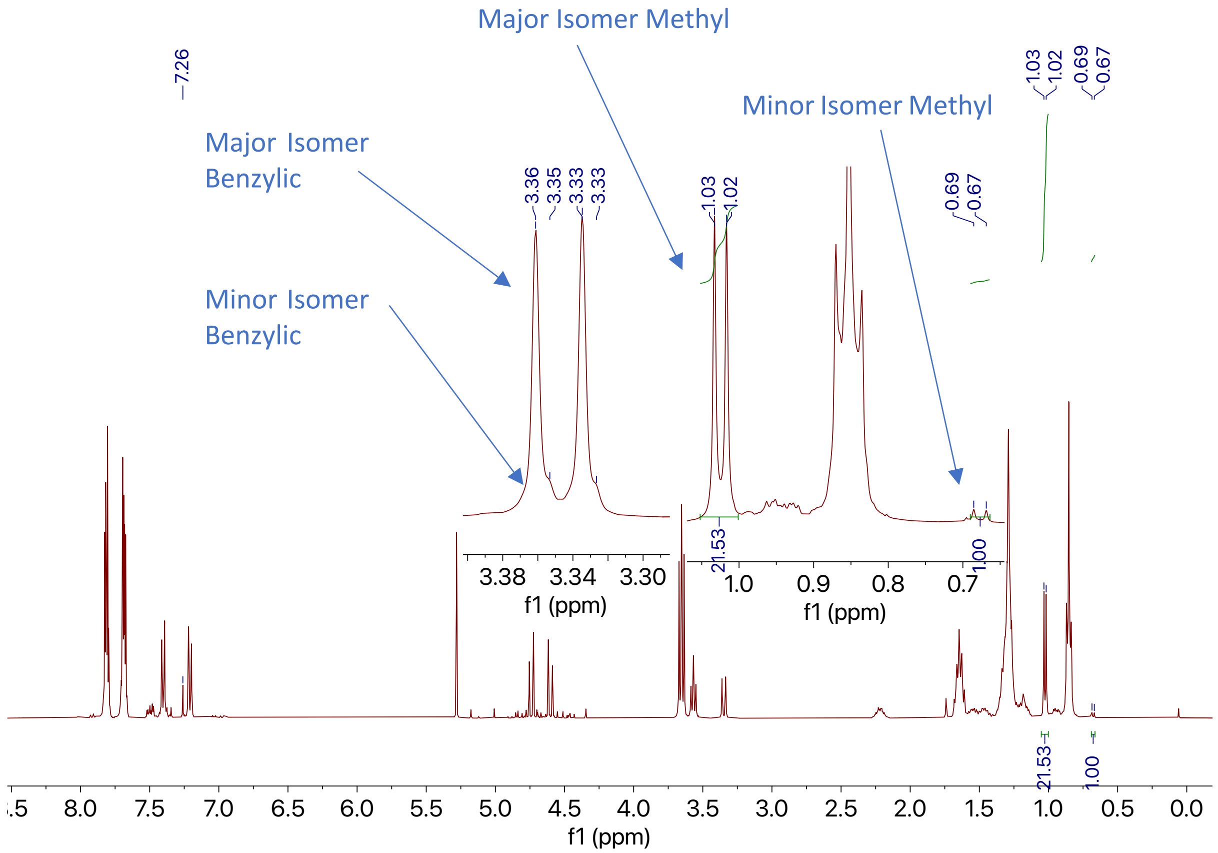

The C2 selectivity is assigned based on the doublet at 3.34 (benzylic H) and the methyl doublets at 1.02 (major) and 0.68 (minor) as previously discussed for related systems[15].

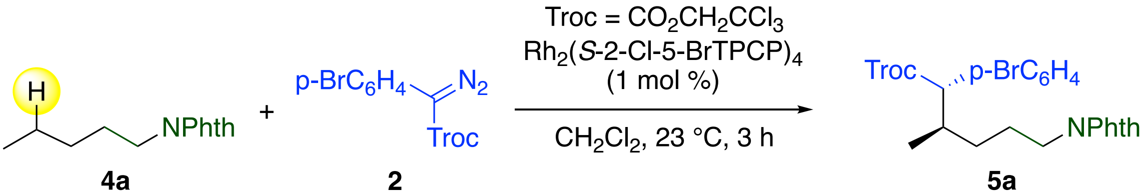

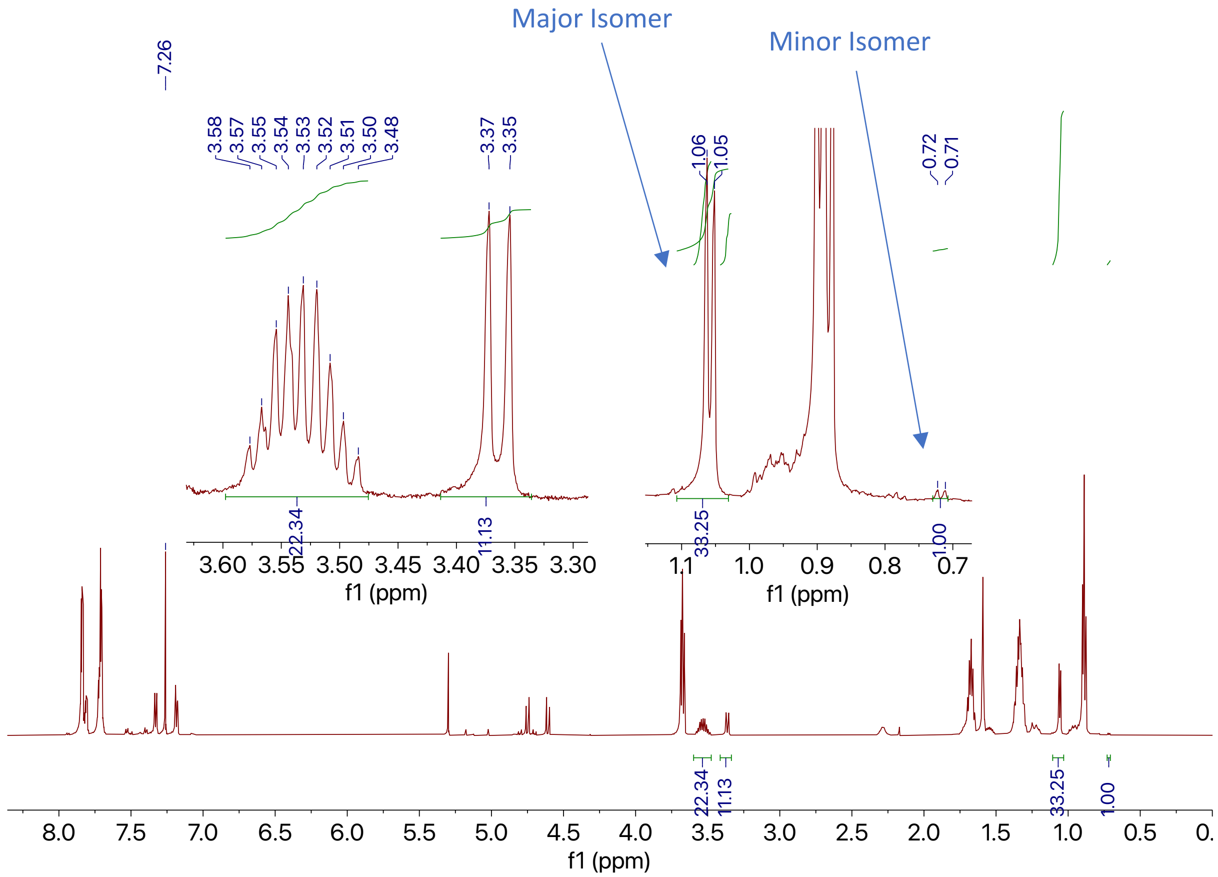

The C2 selectivity is assigned based on the doublet at 3.36 (benzylic H) and the methyl doublets at 1.05 (major) and 0.71 (minor) as previously discussed for related systems[15].

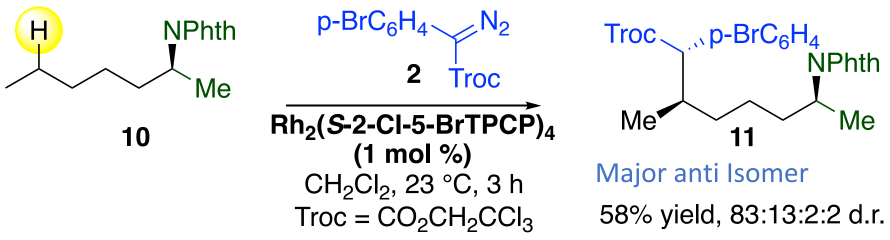

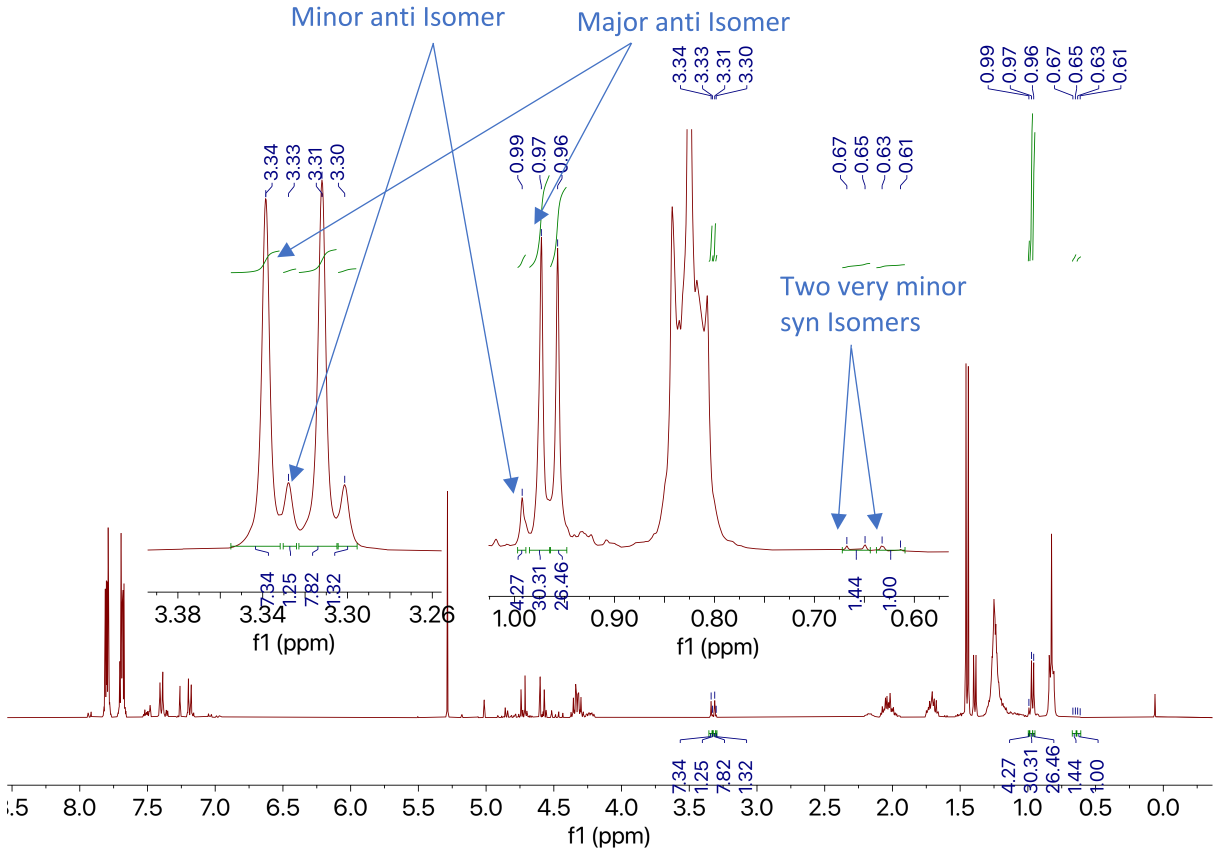

The C2 selectivity is assigned based on the doublet at 3.32 (benzylic H) and the less shielded methyl doublets at 0.98 (major) and 0.96 (minor) as two major diastereomers with benzylic carbon as *R* conformation from **Rh$_2$(*S*-2-Cl-5-BrTPCP)$_4$**, which is similar to previously discussed for related systems[15]. Note that the more shielded methyl doublets at 0.66 (minor) and 0.62 (minor) are distinctive for the two very minor syn diastereomers. The d.r. was determined as 83:13:2:2 from the doublets of the methyl signals.

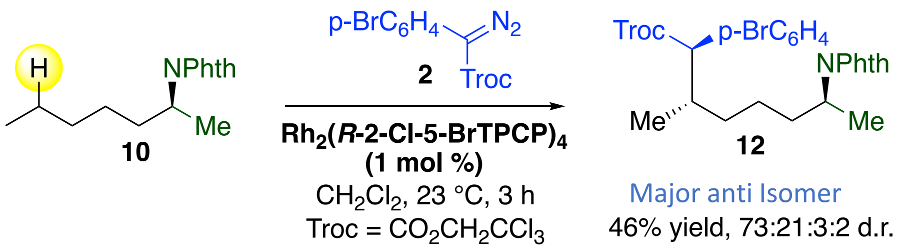

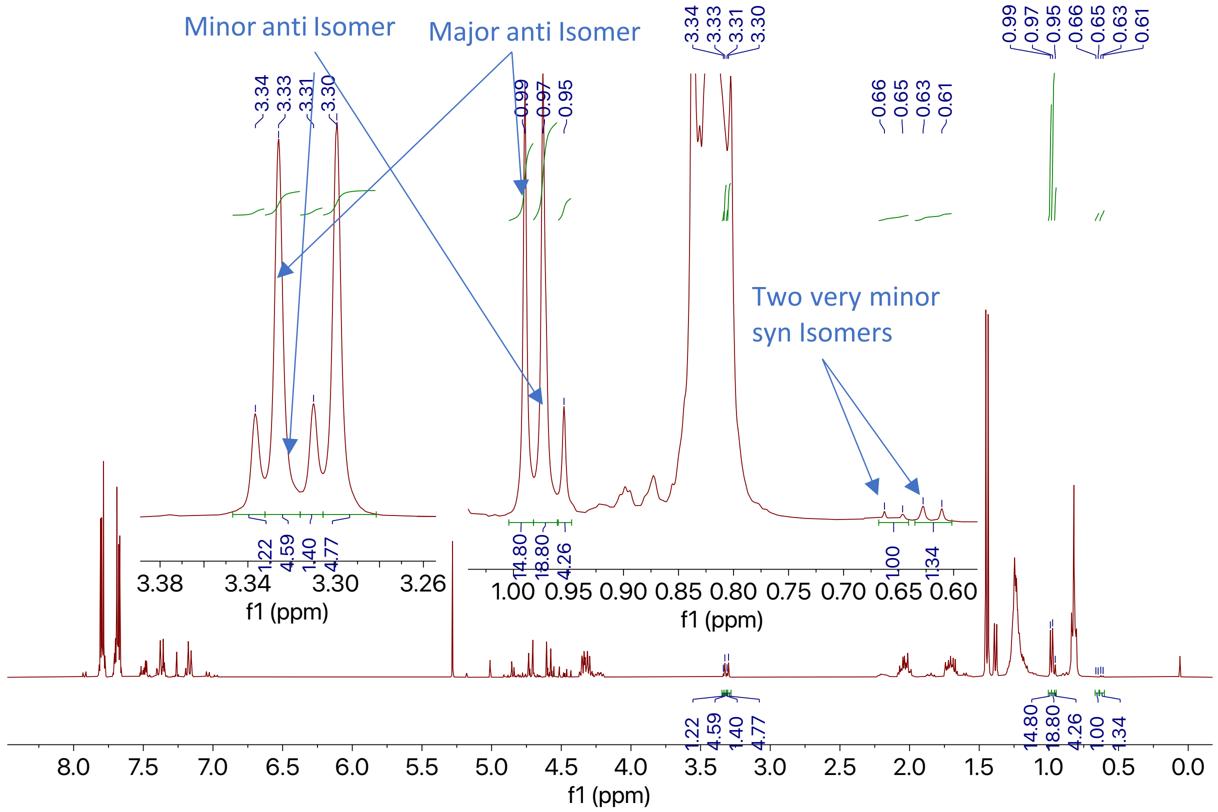

The C2 selectivity is assigned based on the doublet at 3.32 (benzylic H) and the methyl doublets at 0.98 (major) and 0.96 (minor) as two major diastereomers with benzylic carbon as *S* conformation from **Rh_2(*R*-2-Cl-5-BrTPCP)$_4$**, which is similar to previously discussed for related systems[15]. Note that the more shielded methyl doublets at 0.66 (minor) and 0.62 (minor) are distinctive for the two very minor syn diastereomers. The d.r. was determined as 73:21:3:2 from the doublets of the methyl signals.

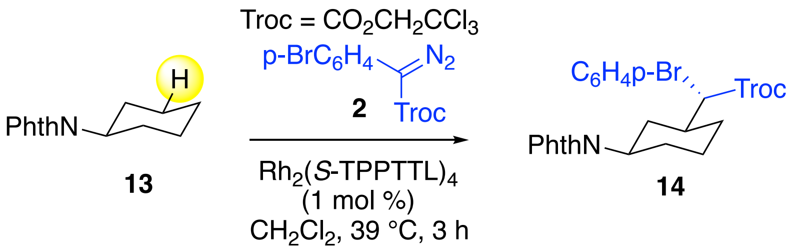

63% yield, 17:1 r.r., >20:1 d.r., 97:3 e.r.

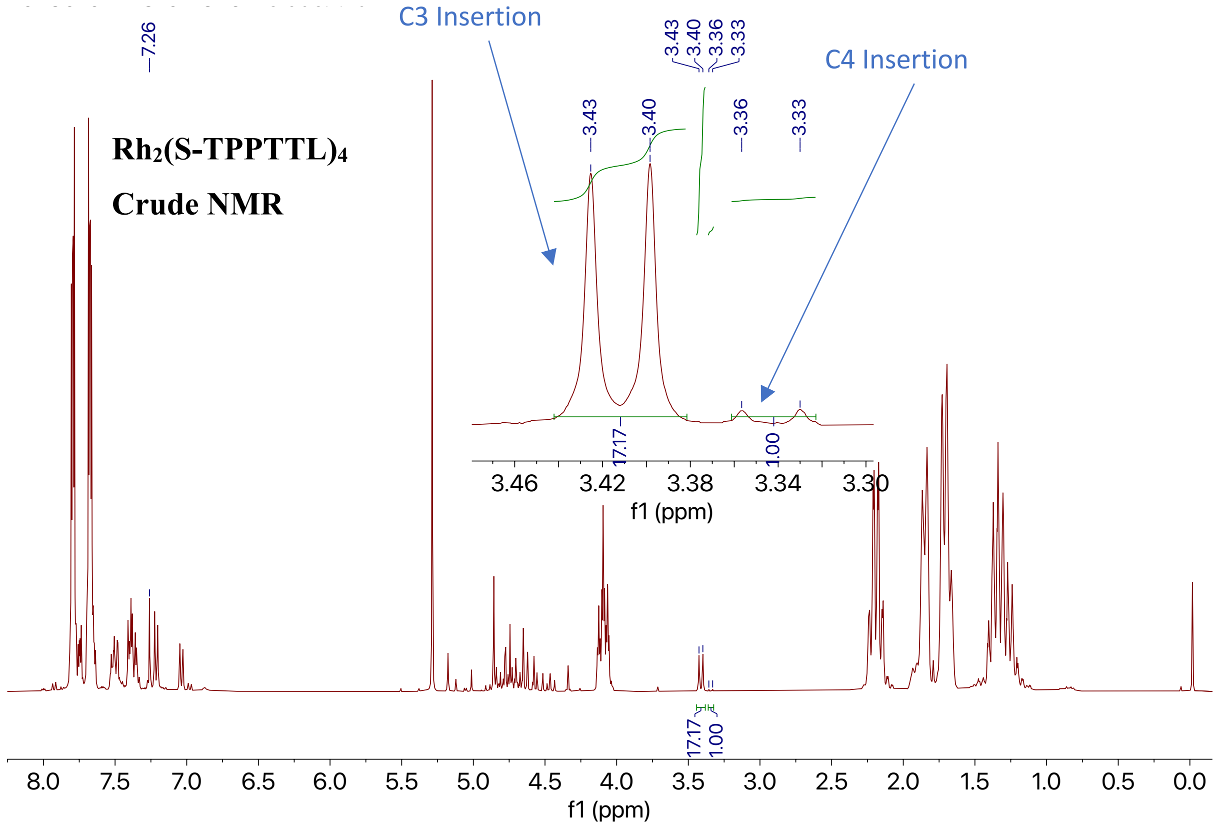

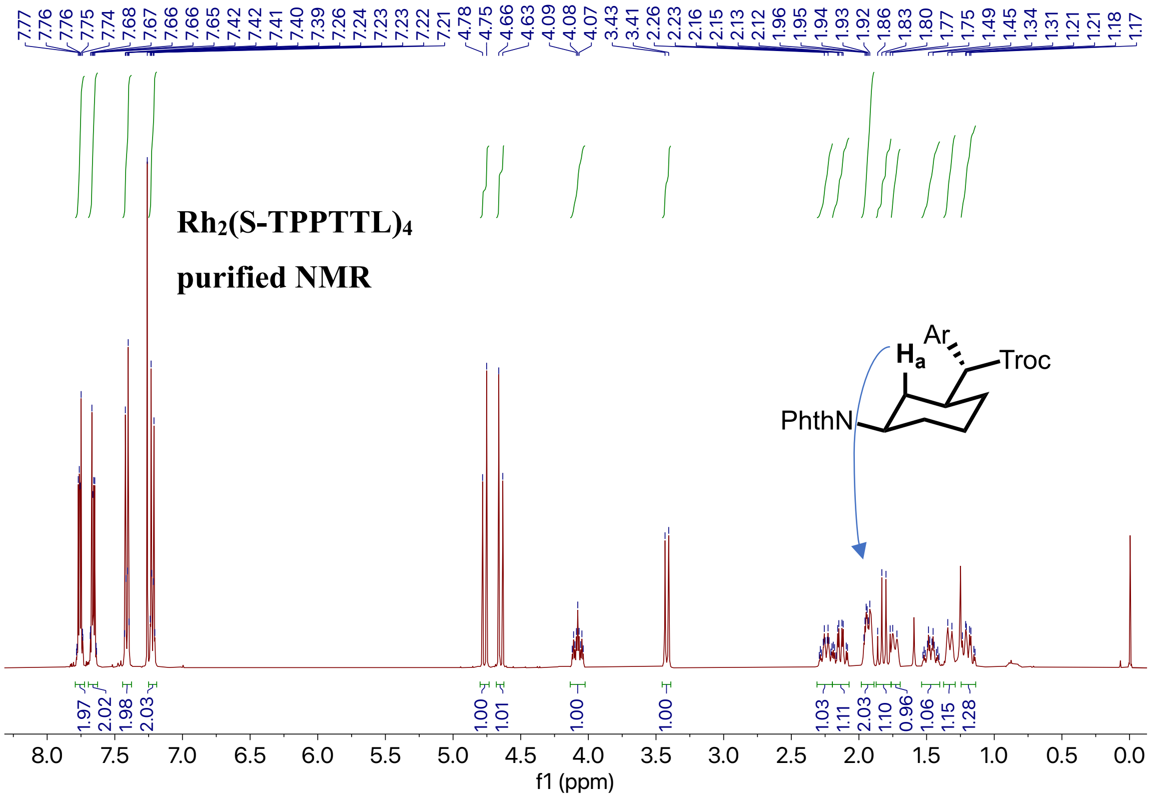

The C3 selectivity is assigned based on the doublet at 3.37 (benzylic H) and the quartet at 1.82 (J = 12.3 Hz) that indicate $\mathbf{H_a}$ in C3 C–H functionalization product as previously discussed for related systems[1]. The following reaction with **Rh_2(*S*-BNP)$_4$ [*S*-BNP: *S*-binaphtholphosphate]** showing all C3, C4 and C5 C–H functionalization products.

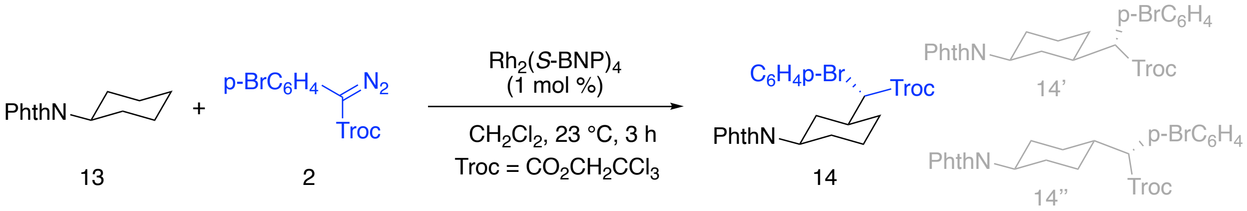

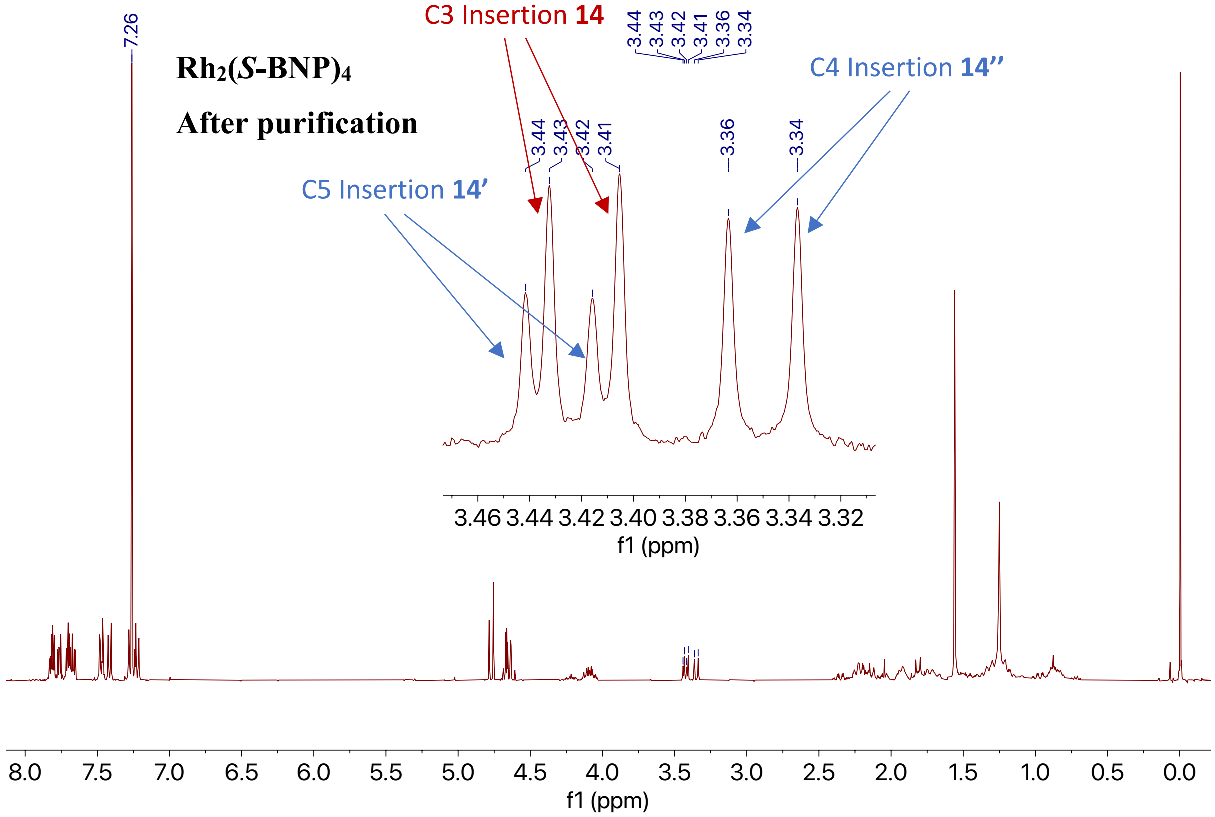

Note that **Rh$_2$(*S*-BNP)$_4$ [*S*-BNP: *S*-binaphtholphosphate]**, which was tested with poor regioselectivity, was used to determine the benzylic proton signal for C5 (3.43 ppm, d, J = 10.8 Hz) and C4 (3.35 ppm, d, J = 10.8 Hz) C–H functionalization products.

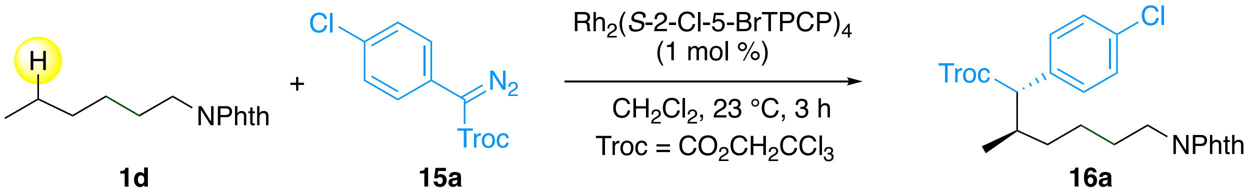

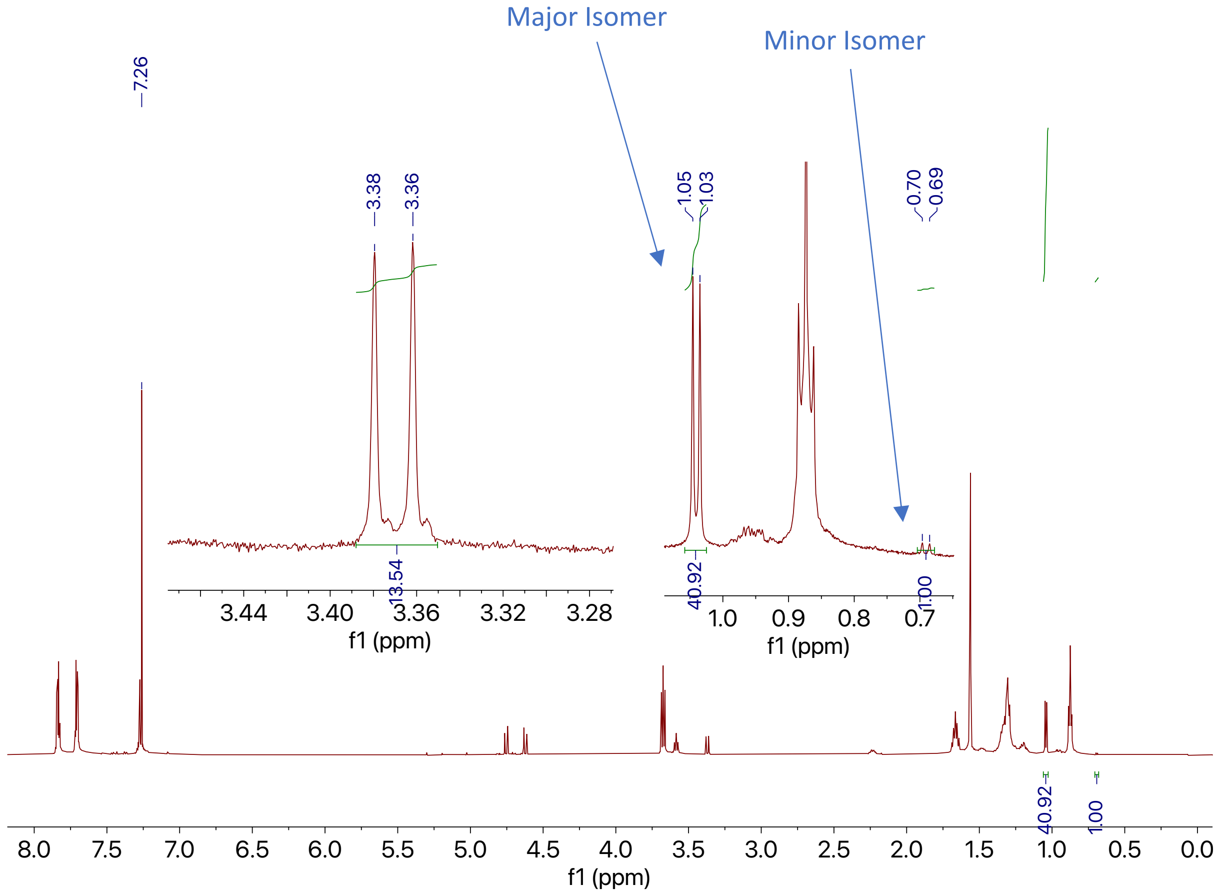

The C2 selectivity is assigned based on the doublet at 3.37 (benzylic H) and the methyl doublets at 1.04 (major) and 0.69 (minor) as previously discussed for related systems[15].

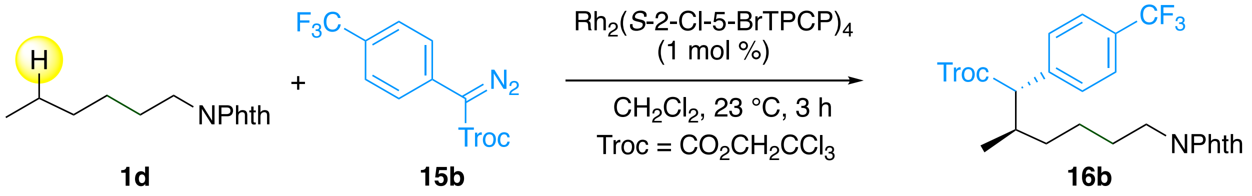

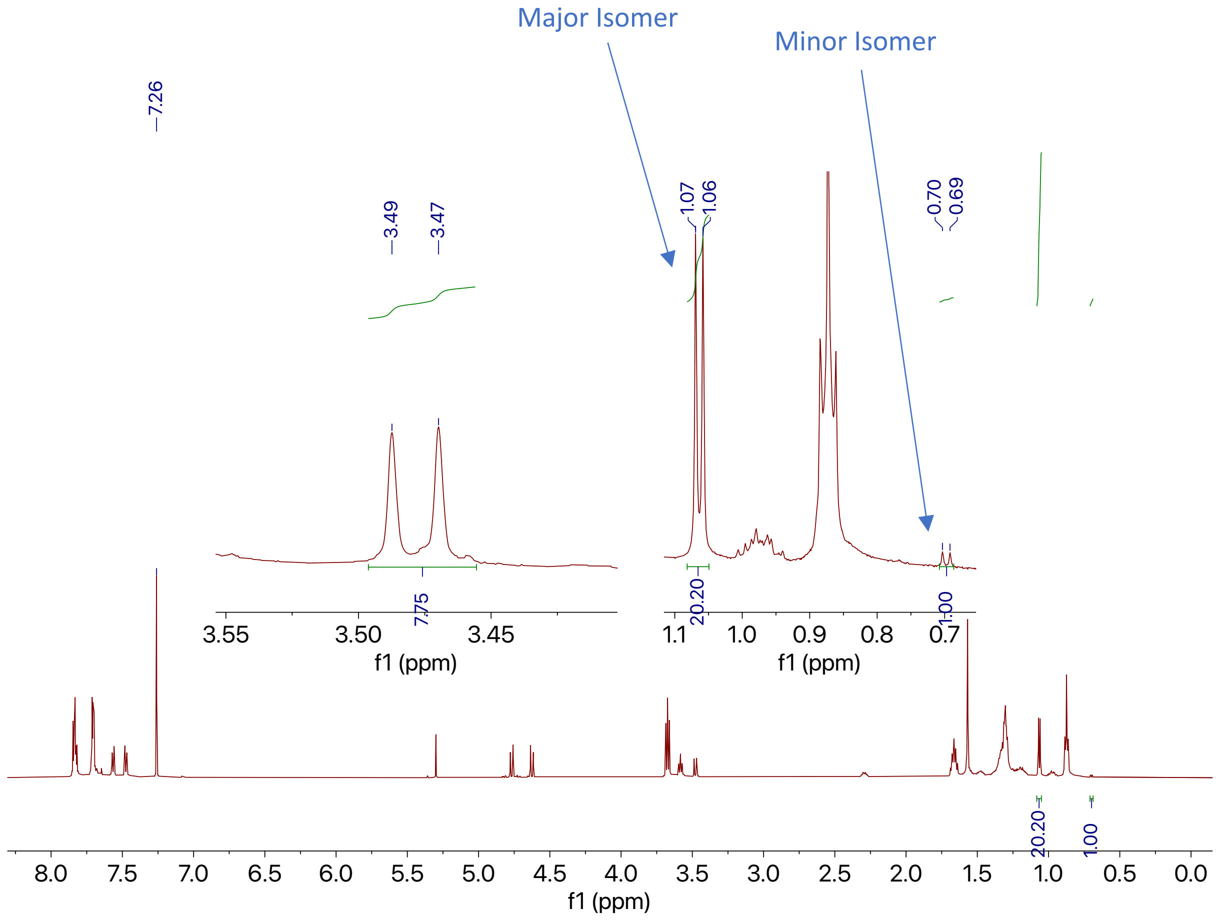

The C2 selectivity is assigned based on the doublet at 3.48 (benzylic H) and the methyl doublets at 1.06 (major) and 0.69 (minor) as previously discussed for related systems[15].

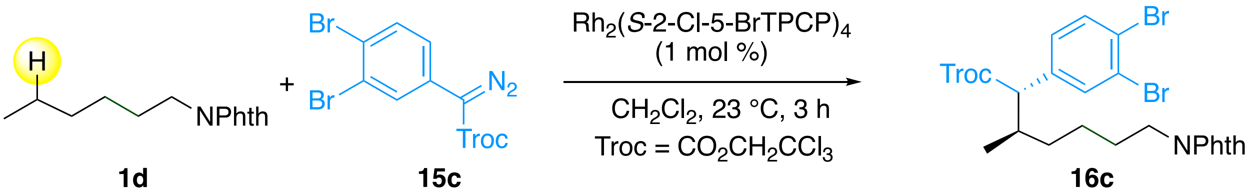

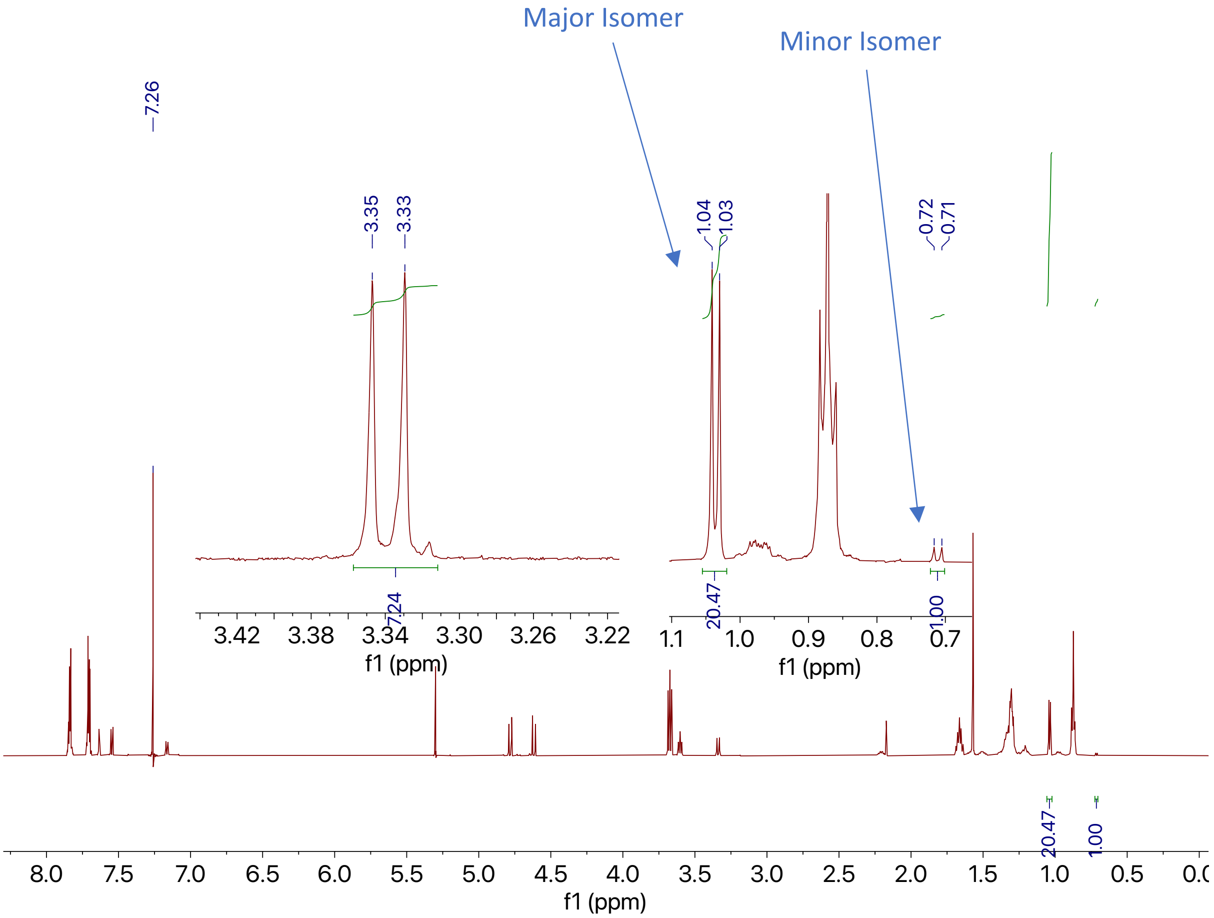

The C2 selectivity is assigned based on the doublet at 3.34 (benzylic H) and the methyl doublets at 1.03 (major) and 0.71 (minor) as previously discussed for related systems[15].

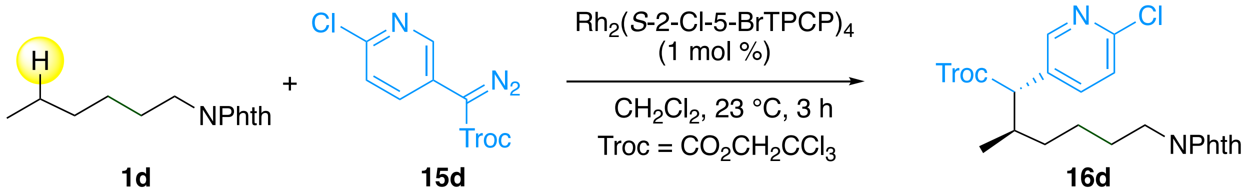

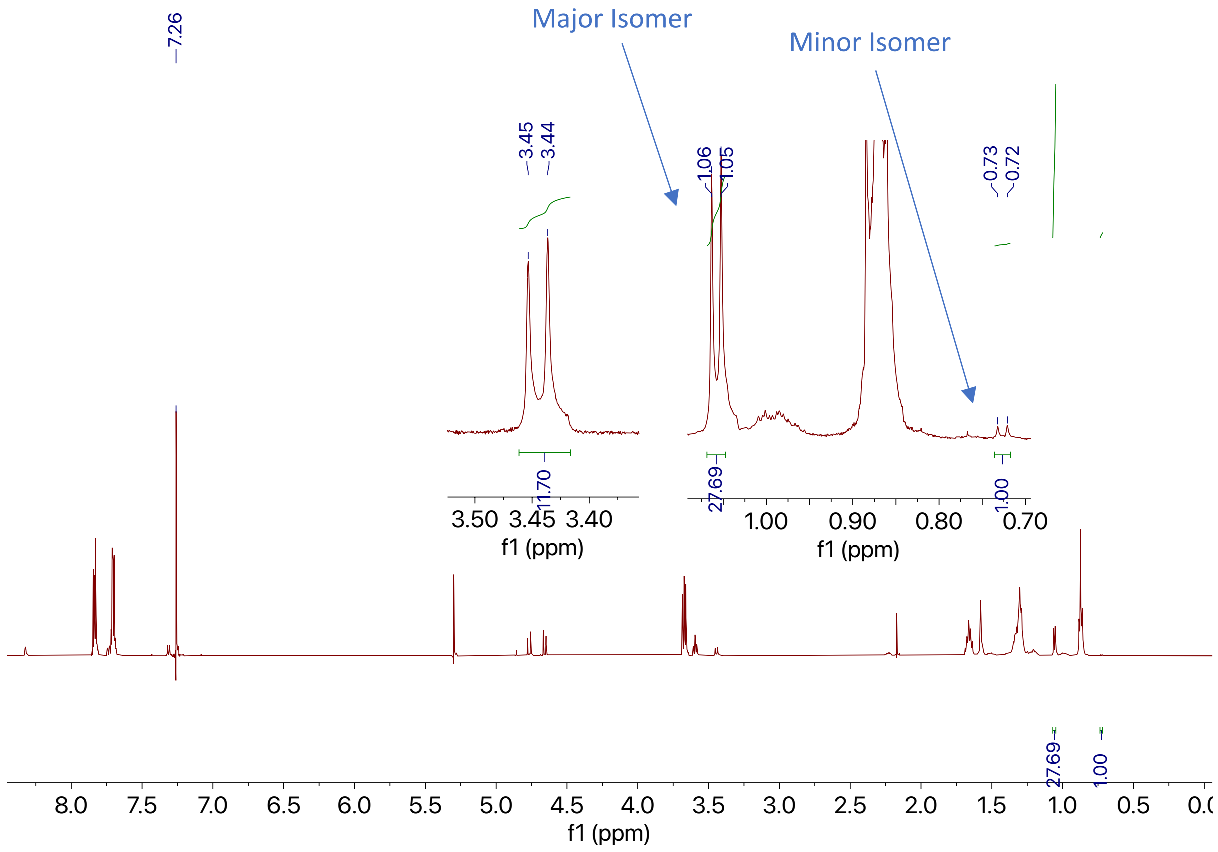

The C2 selectivity is assigned based on the doublet at 3.44 (benzylic H) and the methyl doublets at 1.05 (major) and 0.72 (minor) as previously discussed for related systems[15].

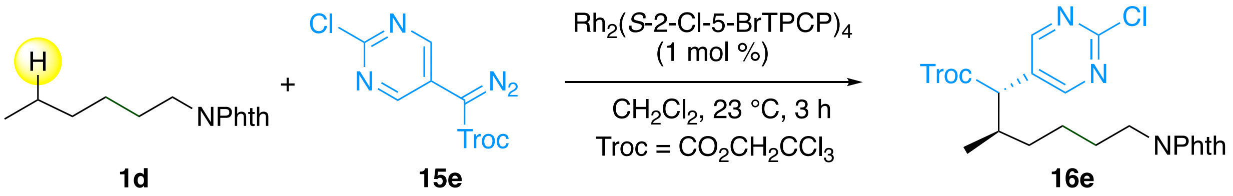

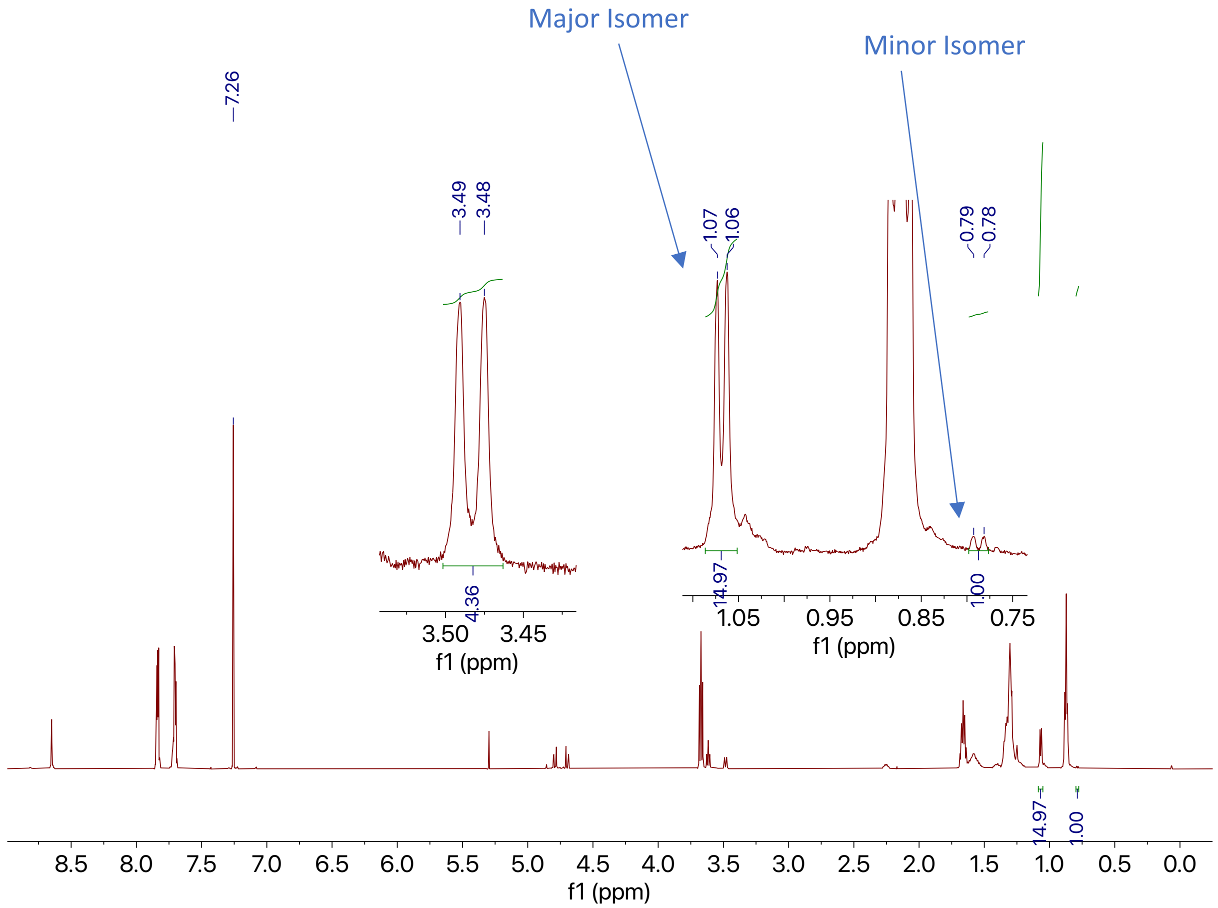

The C2 selectivity is assigned based on the doublet at 3.48 (benzylic H) and the methyl doublets at 1.06 (major) and 0.78 (minor) as previously discussed for related systems[15].

7. Enantioselectivity Determination by HPLC or SFC

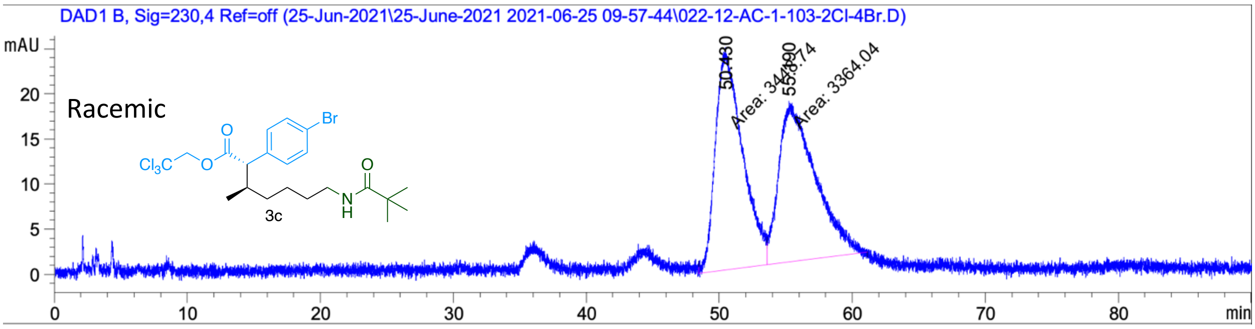

Signal 2: DAD1 B, Sig=230,4 Ref=off

Peak #	RetTime [min]	Type	Width [min]	Area [mAU*s]	Height [mAU]	Area %
1	50.430	MF	2.3259	3448.74316	24.71285	50.6217
2	55.190	FM	3.1333	3364.03662	17.89408	49.3783

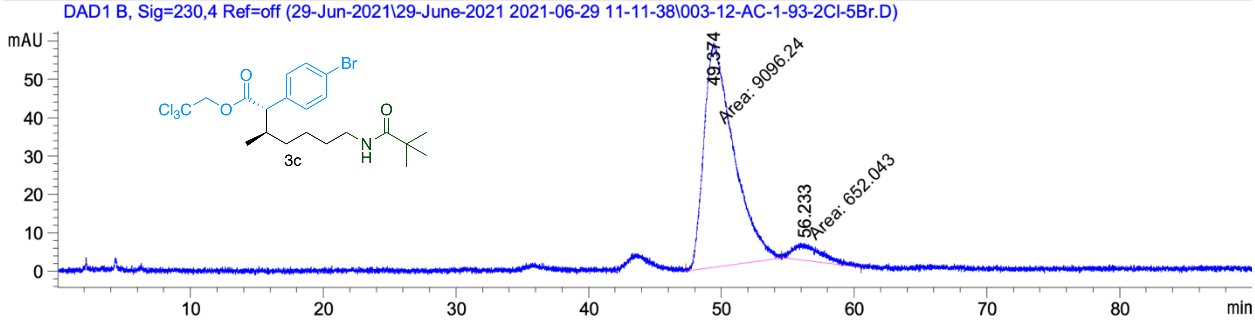

Signal 2: DAD1 B, Sig=230,4 Ref=off

Peak #	RetTime [min]	Type	Width [min]	Area [mAU*s]	Height [mAU]	Area %
1	49.374	MM	2.5889	9096.23730	58.55880	93.3112
2	56.233	MM	2.3747	652.04340	4.57636	6.6888
Totals :				9748.28070	63.13516	

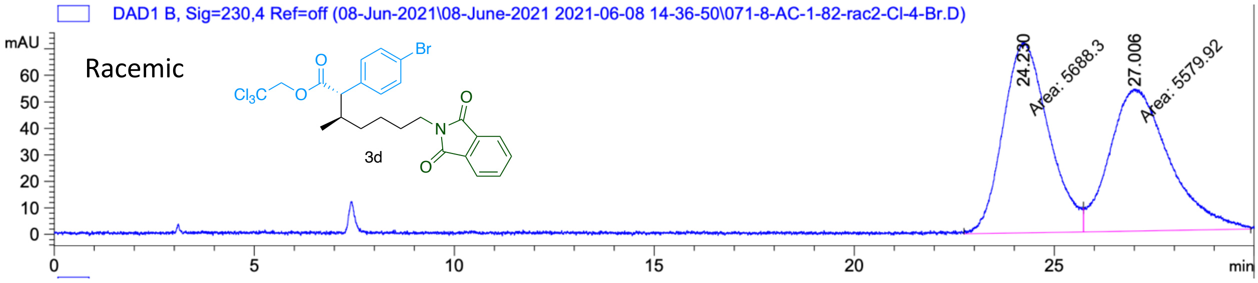

Signal 2: DAD1 B, Sig=230,4 Ref=off

Peak #	RetTime [min]	Type	Width [min]	Area [mAU*s]	Height [mAU]	Area %
1	24.230	MF	1.3197	5688.30420	71.83873	50.4809
2	27.006	FM	1.7350	5579.91699	53.60282	49.5191

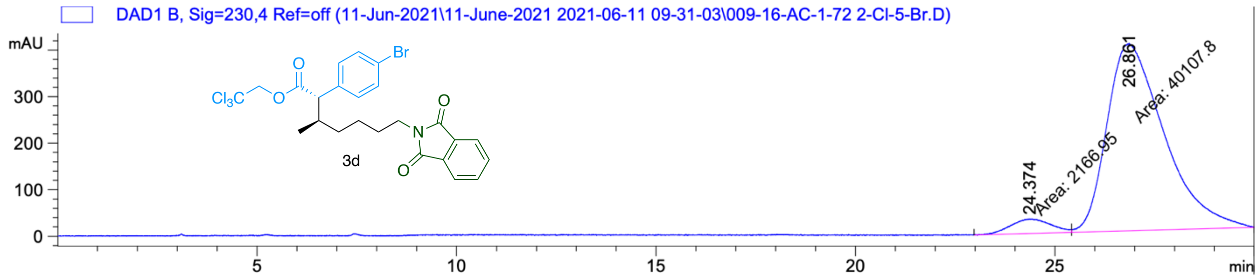

Signal 2: DAD1 B, Sig=230,4 Ref=off

Peak #	RetTime [min]	Type	Width [min]	Area [mAU*s]	Height [mAU]	Area %
1	24.374	MF	1.1637	2166.94727	31.03625	5.1259
2	26.861	FM	1.6594	4.01077e4	402.84210	94.8741

OJ3_1%MeOH_IPA_0_2% Formic Acid_2.5mL/min_20min
AC01273_P7B1_0813_5 Sm (Mn, 2x3)

Diode Array
230
Range: 1.617e-1
Area, Height

Time	Height	Area	Area%
9.44	157538	48050.21	48.36
10.92	144498	51318.25	51.64

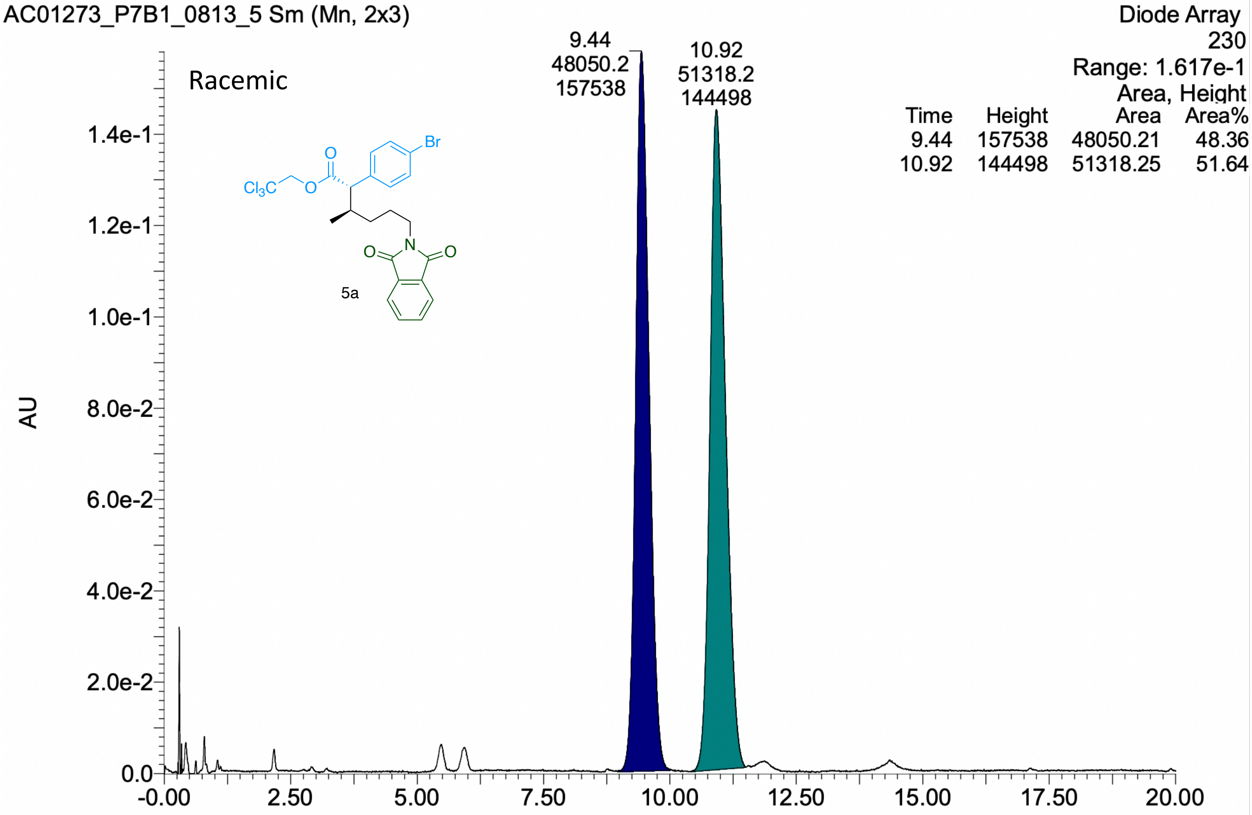

OJ3_1%MeOH_IPA_0_2% Formic Acid_2.5mL/min_20min
AC01200_5a_P7B1_0815_4 Sm (Mn, 2x3)

Diode Array
230
Range: 9.644e-1
Area, Height

Time	Height	Area	Area%
9.31	143204	40062.27	5.65
10.03	959115	669017.63	94.35

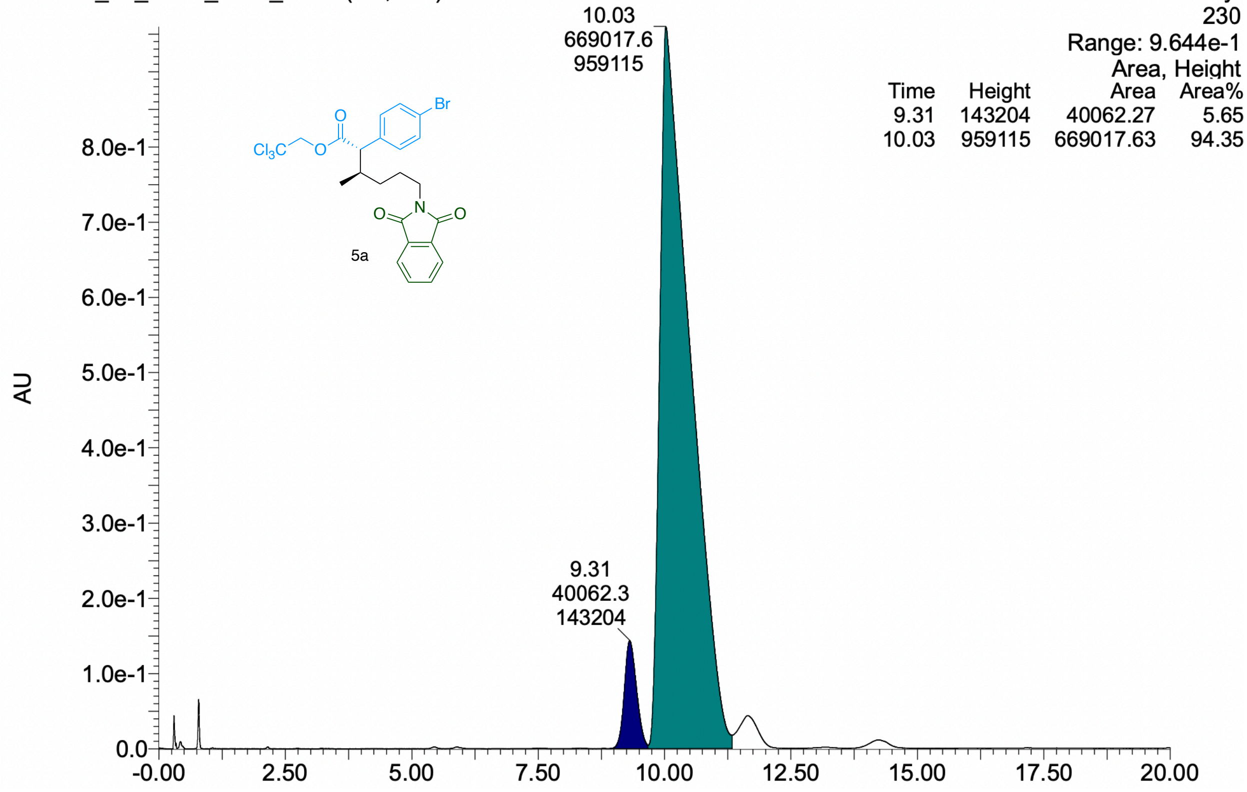

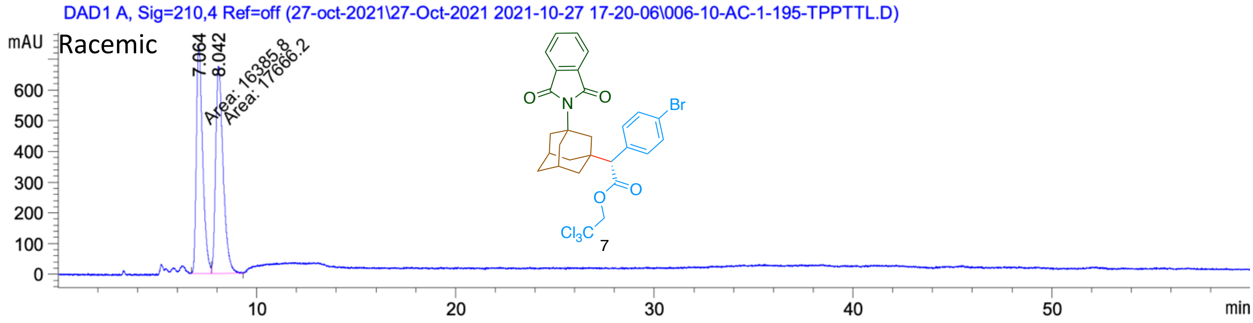

Signal 1: DAD1 A, Sig=210,4 Ref=off

Peak #	RetTime [min]	Type	Width [min]	Area [mAU*s]	Height [mAU]	Area %
1	7.064	MF	0.3653	1.63858e4	747.56378	48.1199
2	8.042	FM	0.4361	1.76662e4	675.20355	51.8801

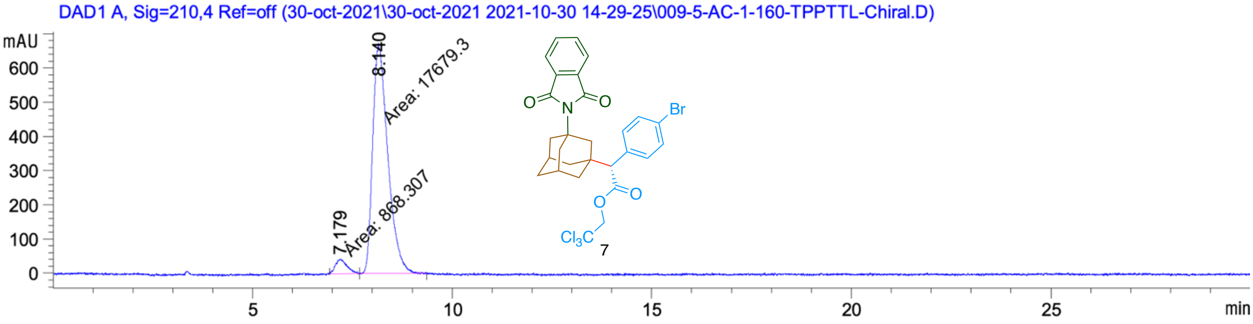

Signal 1: DAD1 A, Sig=210,4 Ref=off

Peak #	RetTime [min]	Type	Width [min]	Area [mAU*s]	Height [mAU]	Area %
1	7.179	MM	0.3348	868.30676	43.23137	4.6815
2	8.140	MM	0.4372	1.76793e4	673.93988	95.3185

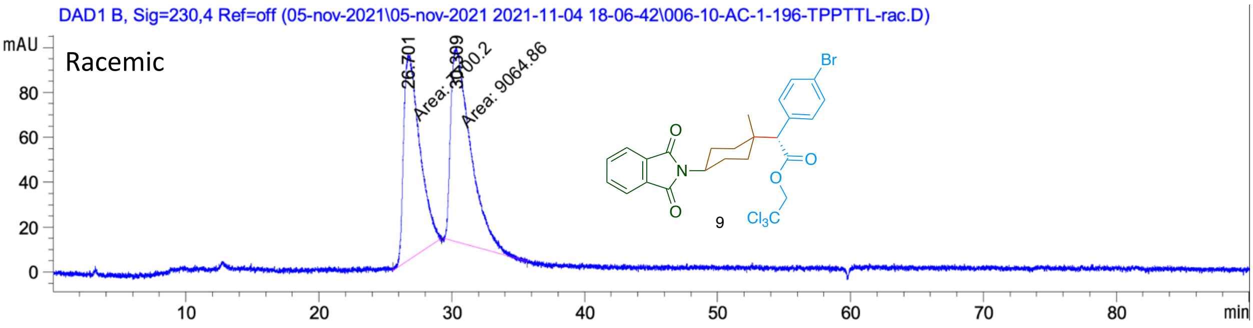

Signal 2: DAD1 B, Sig=230,4 Ref=off

Peak #	RetTime [min]	Type	Width [min]	Area [mAU*s]	Height [mAU]	Area %
1	26.701	MM	1.4012	7700.20410	91.59026	45.9301
2	30.309	MM	1.7358	9064.86035	87.03948	54.0699

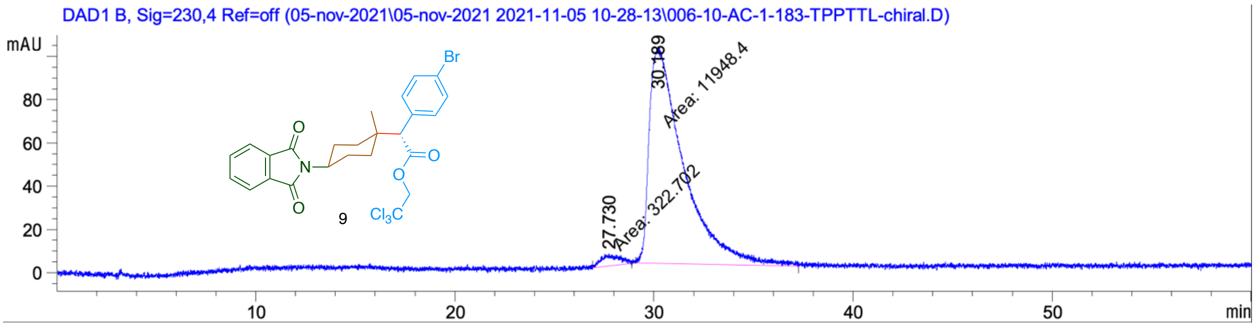

Signal 2: DAD1 B, Sig=230,4 Ref=off

Peak #	RetTime [min]	Type	Width [min]	Area [mAU*s]	Height [mAU]	Area %
1	27.730	MM	0.9398	322.70178	5.72272	2.6298
2	30.189	MM	1.9872	1.19484e4	100.20964	97.3702

Minor diastereomers

OJ3_5%MeOH_IPA_0_2% Formic Acid_2.5mL/min_10min

ZC01272A_P7B1_01032023_2 Sm (Mn, 2x3)

Diode Array
230
Range: 1.042
Area, Height

Time	Height	Area	Area%
4.33	1039308	177723.13	48.88
7.74	557489	185866.36	51.12

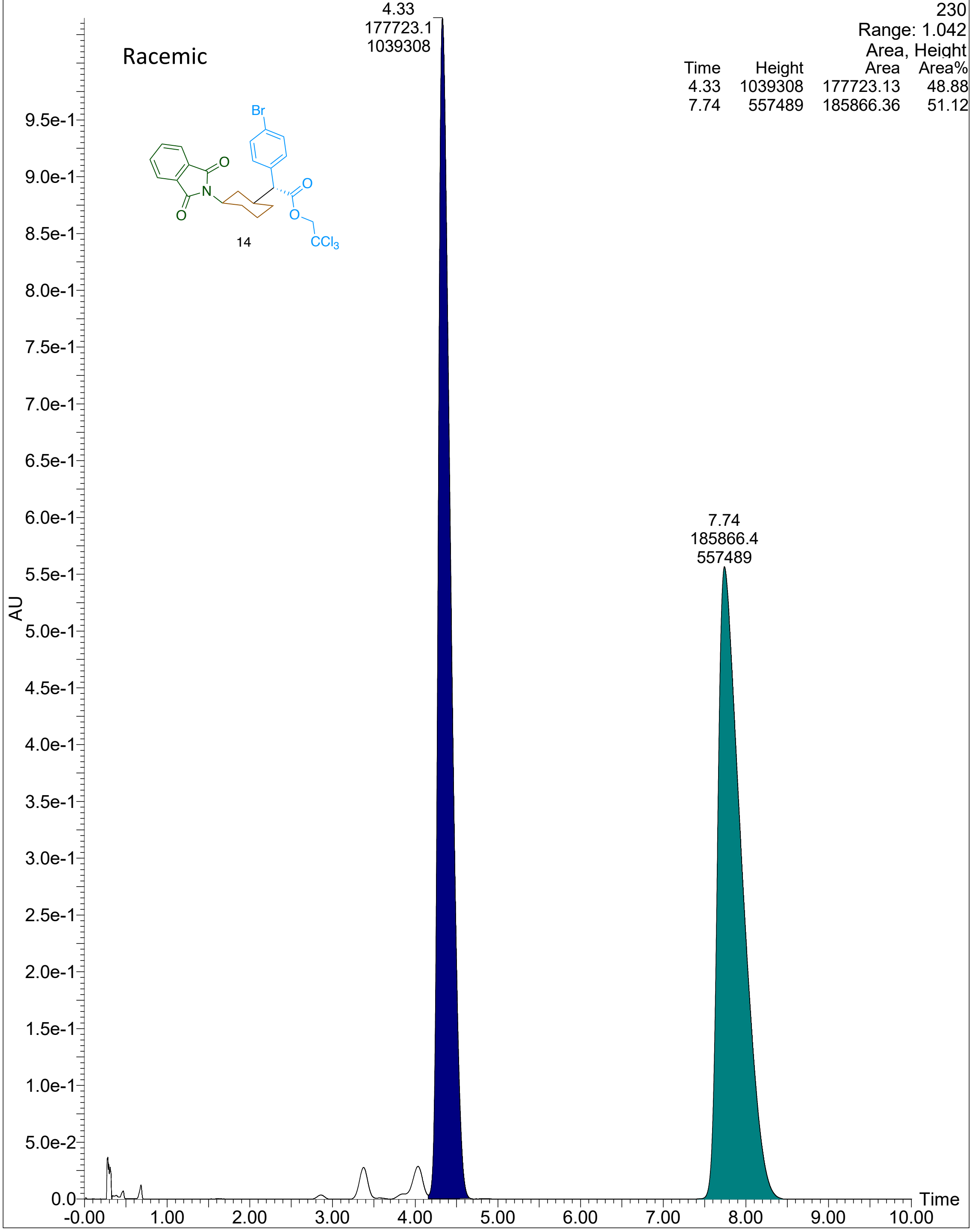

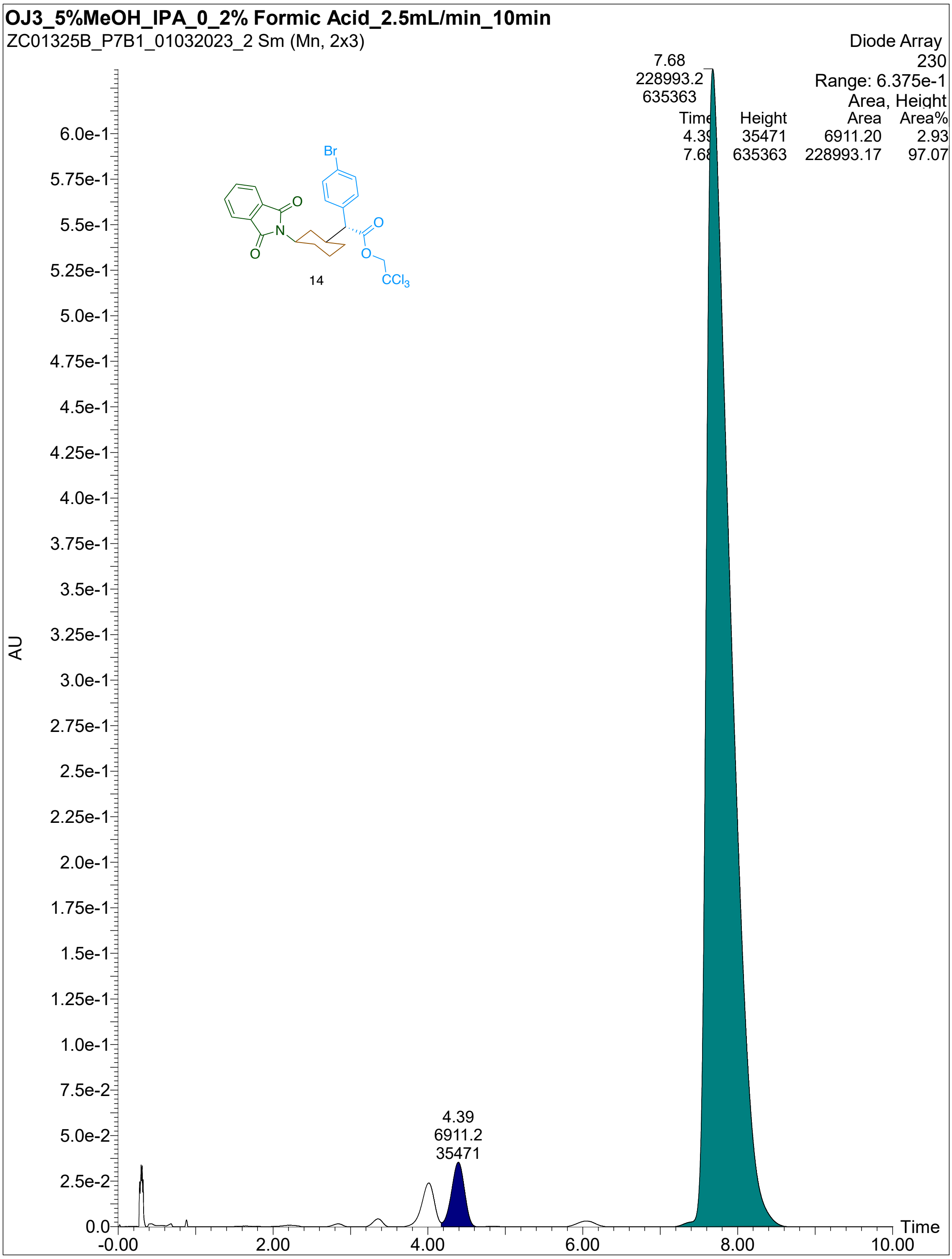

OJ3_5%MeOH_IPA_0_2% Formic Acid_2.5mL/min_10min
ZC01325B_P7B1_01032023_2 Sm (Mn, 2x3)
Diode Array
230
Range: 6.375e-1
Area, Height
Time Height Area Area%
4.39 35471 6911.20 2.93
7.68 635363 228993.17 97.07
7.68
228993.2
635363
4.39
6911.2
35471
Br
O
N
O
O
O
CCl3
14
AU
Time
6.0e-1
5.75e-1
5.5e-1
5.25e-1
5.0e-1
4.75e-1
4.5e-1
4.25e-1
4.0e-1
3.75e-1
3.5e-1
3.25e-1
3.0e-1
2.75e-1
2.5e-1
2.25e-1
2.0e-1
1.75e-1
1.5e-1
1.25e-1
1.0e-1
7.5e-2
5.0e-2
2.5e-2
0.0
-0.00
2.00
4.00
6.00
8.00
10.00

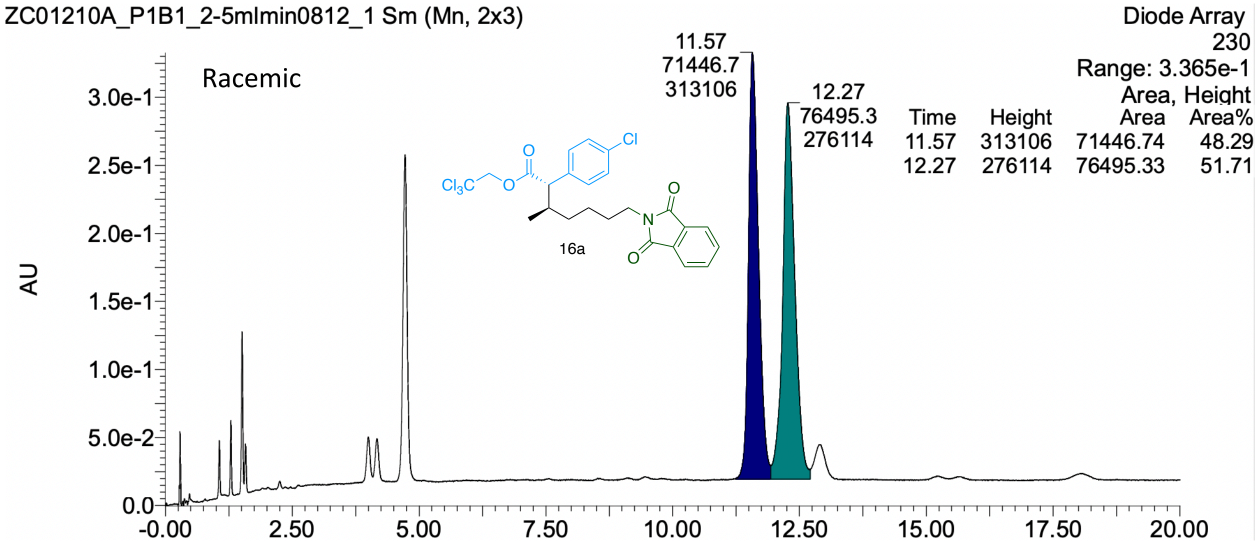

ZC01210A_P1B1_2-5mlmin0812_1 Sm (Mn, 2x3)
Diode Array
230
Range: 3.365e-1
Area, Height
Racemic
11.57
71446.7
313106
12.27
76495.3
276114
Time Height Area Area%
11.57 313106 71446.74 48.29
12.27 276114 76495.33 51.71
16a
AU
3.0e-1
2.5e-1
2.0e-1
1.5e-1
1.0e-1
5.0e-2
0.0
-0.00
2.50
5.00
7.50
10.00
12.50
15.00
17.50
20.00

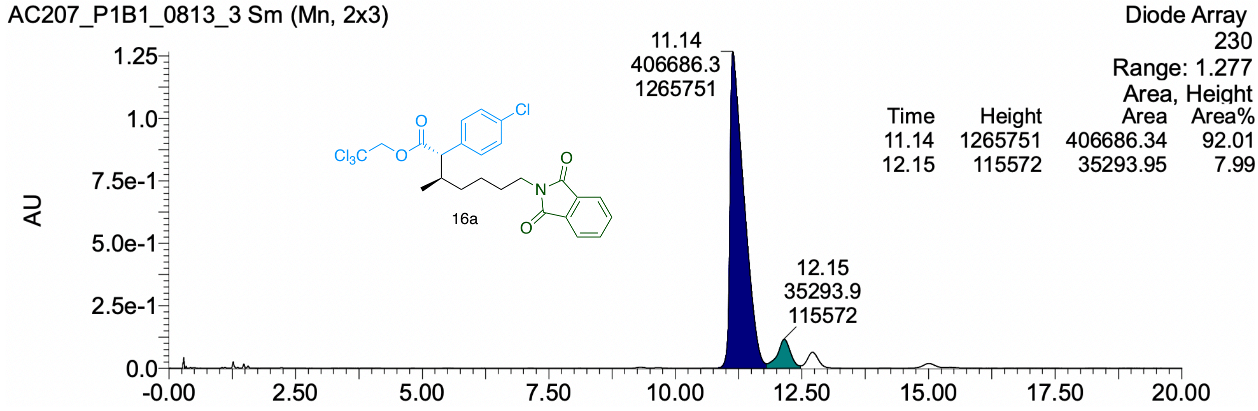

AC207_P1B1_0813_3 Sm (Mn, 2x3)
Diode Array
230
Range: 1.277
Area, Height
11.14
406686.3
1265751
Time Height Area Area%
11.14 1265751 406686.34 92.01
12.15 115572 35293.95 7.99
16a
12.15
35293.9
115572
AU
1.25
1.0
7.5e-1
5.0e-1
2.5e-1
0.0
-0.00
2.50
5.00
7.50
10.00
12.50
15.00
17.50
20.00

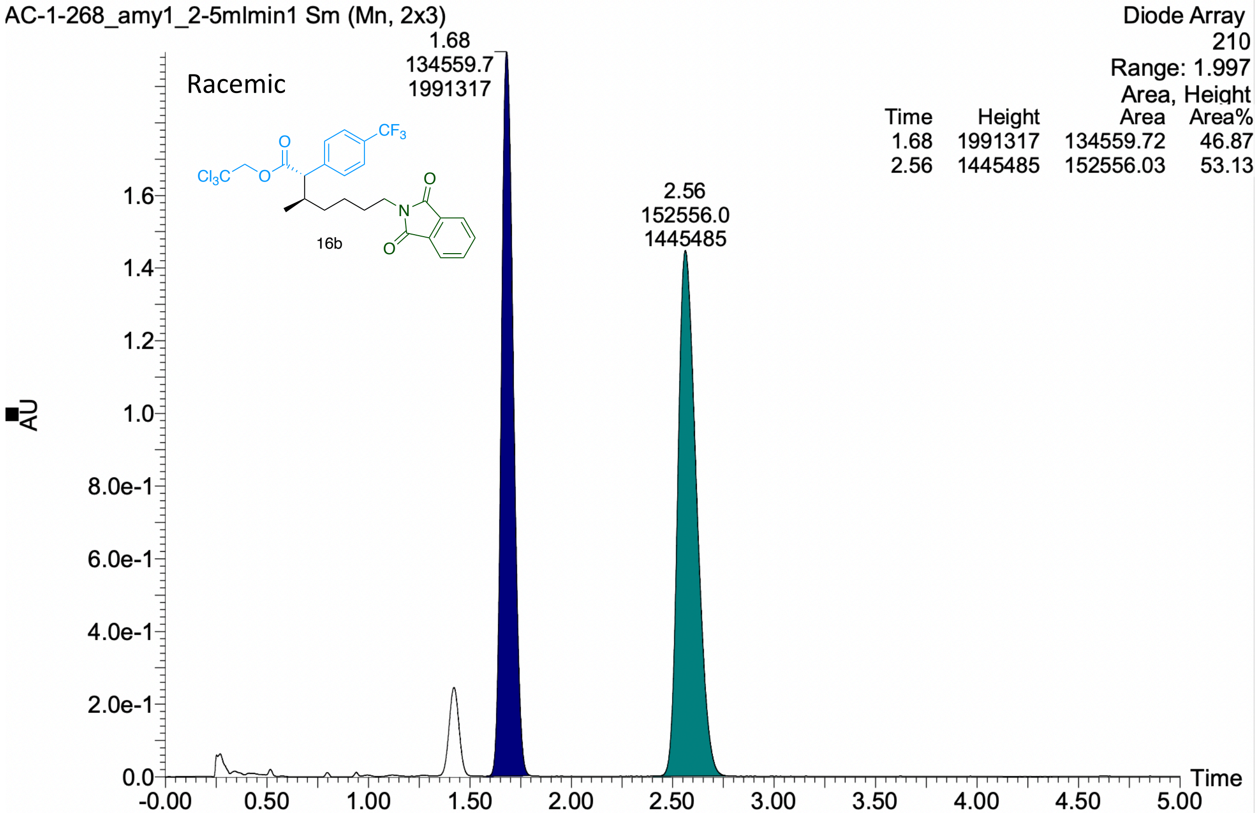

AC-1-268_amy1_2-5mlmin1 Sm (Mn, 2x3)
Diode Array
210
Range: 1.997
Area, Height
Racemic
1.68
134559.7
1991317
Time Height Area Area%
1.68 1991317 134559.72 46.87
2.56 1445485 152556.03 53.13
16b
2.56
152556.0
1445485
AU
1.6
1.4
1.2
1.0
8.0e-1
6.0e-1
4.0e-1
2.0e-1
0.0
-0.00
0.50
1.00
1.50
2.00
2.50
3.00
3.50
4.00
4.50
5.00
Time

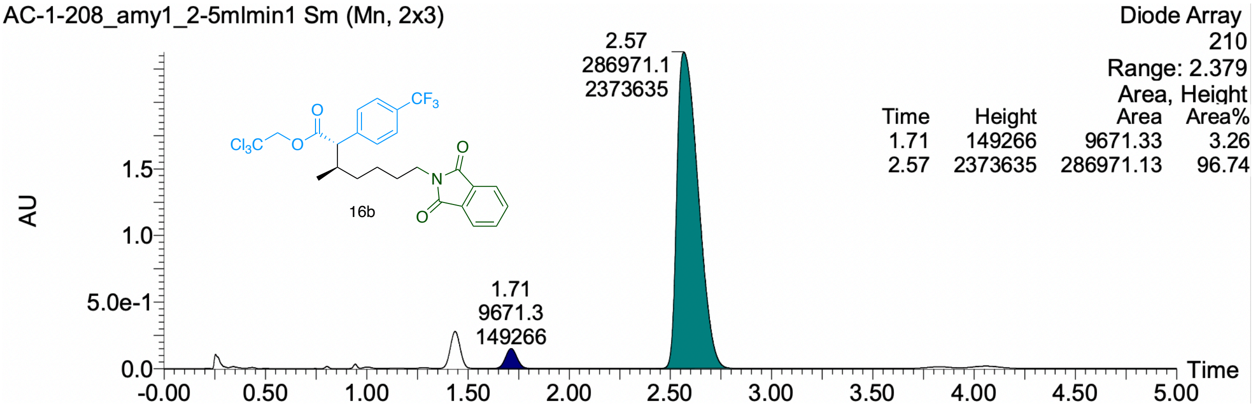

Time	Height	Area	Area%
1.71	149266	9671.33	3.26
2.57	2373635	286971.13	96.74

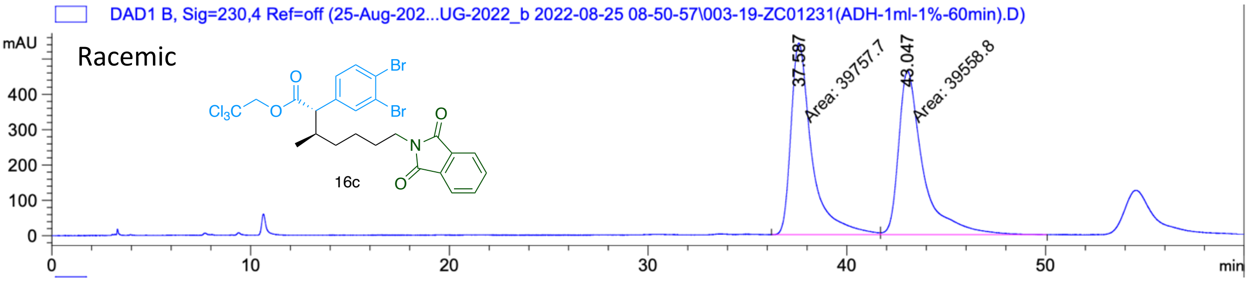

Signal 2: DAD1 B, Sig=230,4 Ref=off

Peak #	RetTime [min]	Type	Width [min]	Area [mAU*s]	Height [mAU]	Area %
1	37.587	MF	1.2214	3.97577e4	542.51288	50.1254
2	43.047	FM	1.4246	3.95588e4	462.79553	49.8746

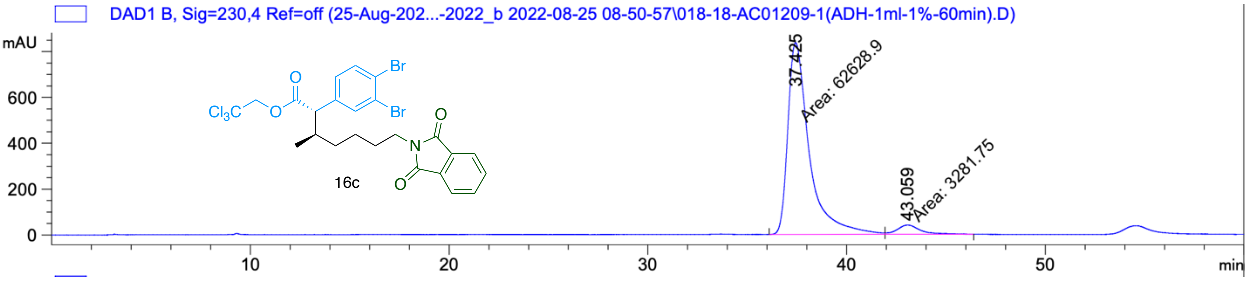

Signal 2: DAD1 B, Sig=230,4 Ref=off

Peak #	RetTime [min]	Type	Width [min]	Area [mAU*s]	Height [mAU]	Area %
1	37.425	MF	1.2473	6.26289e4	836.83557	95.0209
2	43.059	FM	1.3479	3281.75195	40.57981	4.9791

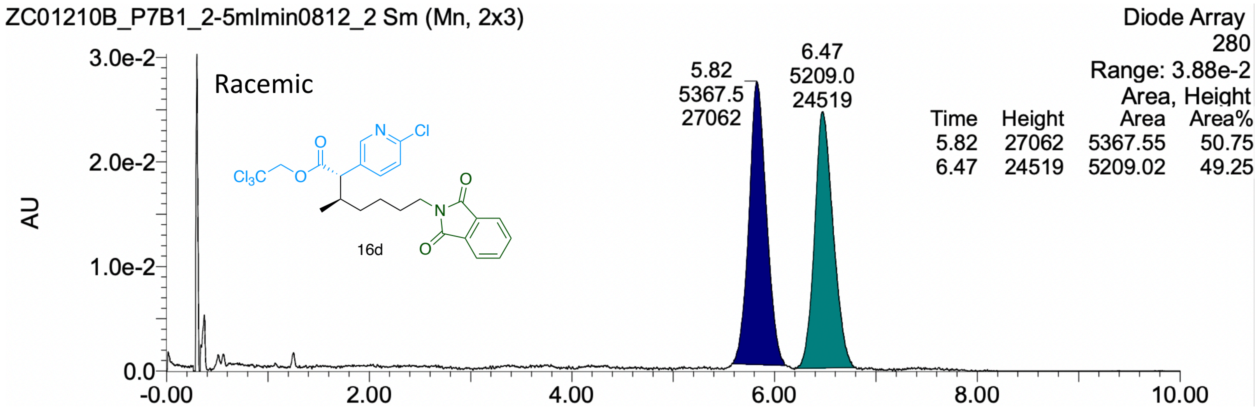

ZC01210B_P7B1_2-5mlmin0812_2 Sm (Mn, 2x3)
Diode Array
280
Range: 3.88e-2
Area, Height
Racemic
16d
AU
5.82
5367.5
27062
6.47
5209.0
24519
Time Height Area Area%
5.82 27062 5367.55 50.75
6.47 24519 5209.02 49.25

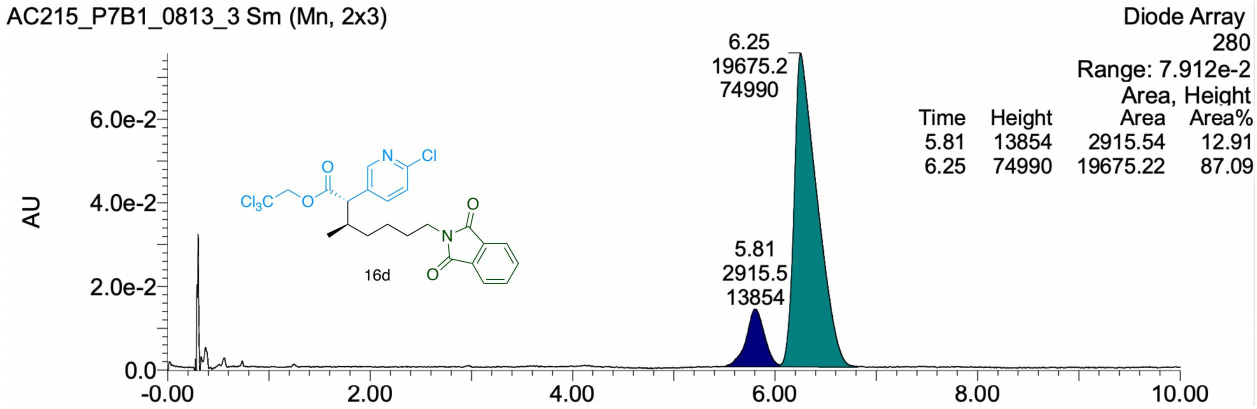

AC215_P7B1_0813_3 Sm (Mn, 2x3)
Diode Array
280
Range: 7.912e-2
Area, Height
16d
AU
6.25
19675.2
74990
5.81
2915.5
13854
Time Height Area Area%
5.81 13854 2915.54 12.91
6.25 74990 19675.22 87.09

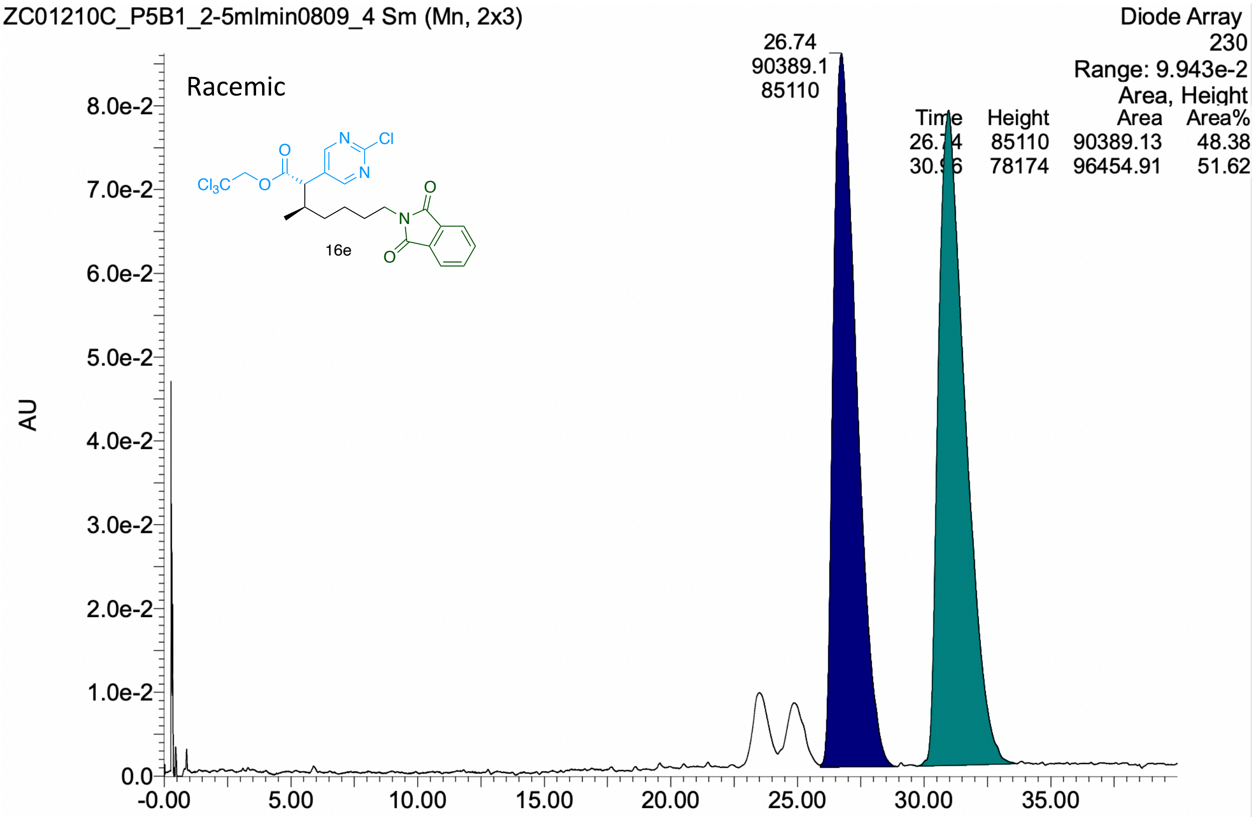
ZC01210C_P5B1_2-5mlmin0809_4 Sm (Mn, 2x3)
Diode Array
230
Range: 9.943e-2
Area, Height
Racemic
16e
26.74
90389.1
85110
Time Height Area Area%
26.74 85110 90389.13 48.38
30.96 78174 96454.91 51.62
AU
8.0e-2
7.0e-2
6.0e-2
5.0e-2
4.0e-2
3.0e-2
2.0e-2
1.0e-2
0.0
-0.00
5.00
10.00
15.00
20.00
25.00
30.00
35.00

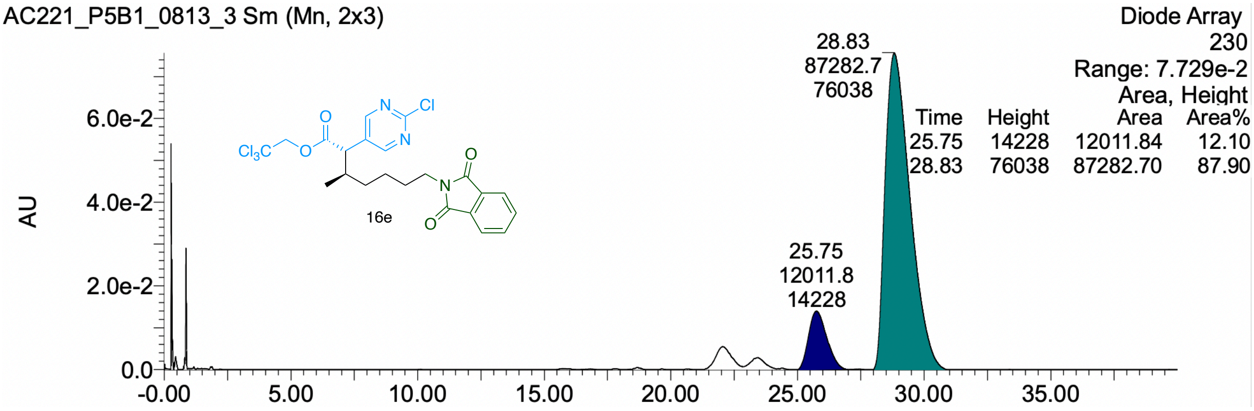
AC221_P5B1_0813_3 Sm (Mn, 2x3)
Diode Array
230
Range: 7.729e-2
Area, Height
16e
28.83
87282.7
76038
25.75
12011.8
14228
Time Height Area Area%
25.75 14228 12011.84 12.10
28.83 76038 87282.70 87.90
AU
6.0e-2
4.0e-2
2.0e-2
0.0
-0.00
5.00
10.00
15.00
20.00
25.00
30.00
35.00

8. *X-Ray Crystallographic Data for Compound 9*

Compound 9 (zc-01-255b)

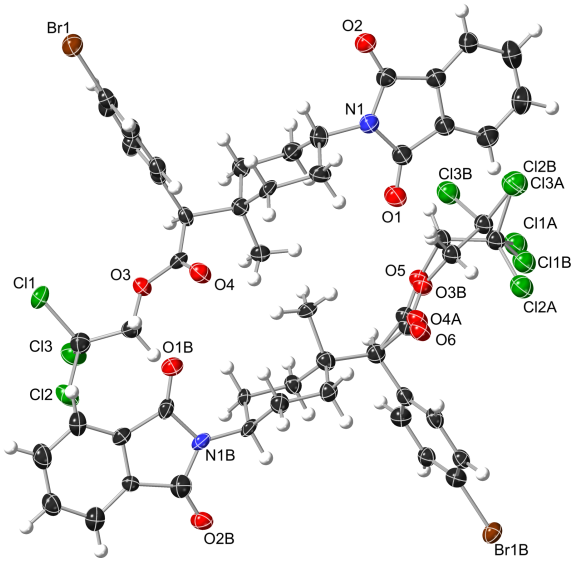

Thermal ellipsoidal (50% probability) representation of the molecular structure

Experimental: The material was recrystallised from a mixture of CH_2Cl_2 and n-Hexane by slow evaporation as supplied. The data for zc-01-255b were collected from a single crystal at 100(2) K on an XtaLAB Synergy, Dualflex, HyPix four-circle diffractometer with a micro-focus sealed X-ray tube using a multilayer mirror as monochromator and a HyPix detector. The diffractometer was equipped with an Oxford Cryostream 700 low temperature device and used Cu K_α radiation (λ = 1.54178 Å). All data were integrated with CrysAlisPro and a analytical absorption correction using SCALE3 ABSPACK was applied. [1,2] The structure was solved by dual methods with SHELXT and refined by full-matrix least-squares methods against F^2 using SHELXL-2014.[3,4] All non-hydrogen atoms were refined with anisotropic displacement parameters. The hydrogen atoms were refined isotropically on calculated positions using a riding model with their U_{iso} values constrained to 1.5 times the U_{eq} of their pivot atoms for terminal sp^3 carbon atoms and 1.2 times for all other carbon atoms. Crystallographic data for the structures reported in this paper have been deposited with the Cambridge Crystallographic Data Centre.[5] CCDC 2247866 contain the supplementary crystallographic data for this paper. These data can be obtained free of charge from The Cambridge Crystallographic Data Centre via www.ccdc.cam.ac.uk/structures. This report and the CIF file were generated using FinalCif.[6]

Table 1. Crystal data and structure refinement for zc-01-255b

CCDC number	2247866
Empirical formula	$C_{25}H_{23}BrCl_3NO_4$
Formula weight	587.70
Temperature [K]	100(2)
Crystal system	triclinic
Space group (number)	$P1$ (1)
a [Å]	10.1505(6)
b [Å]	10.3802(4)
c [Å]	13.4889(4)
α [°]	99.627(3)
β [°]	102.406(4)
γ [°]	111.913(5)
Volume [Å^3]	1238.84(11)
Z	2
ρ_{calc} [gcm^{-3}]	1.576
μ [mm^{-1}]	5.512
F(000)	596
Crystal size [mm^3]	0.052×0.111×0.178
Crystal colour	colourless
Crystal shape	prism
Radiation	Cu K_α (λ=1.54178 Å)
2θ range [°]	6.97 to 136.49 (0.83 Å)
Index ranges	$-12 \le h \le 12$<br>$-12 \le k \le 12$<br>$-16 \le l \le 14$
Reflections collected	16868
Independent reflections	6357<br>R_{int} = 0.0642<br>R_{sigma} = 0.0703
Completeness to θ = 67.684°	98.3 %
Data / Restraints / Parameters	6357 / 238 / 622
Goodness-of-fit on F^2	1.159
Final R indexes [$I \ge 2\sigma(I)$]	R_1 = 0.1004<br>wR_2 = 0.2547
Final R indexes [all data]	R_1 = 0.1039<br>wR_2 = 0.2606
Largest peak/hole [eÅ^{-3}]	3.44/−1.64
Flack X parameter	0.00(3)

Table 2. Atomic coordinates and Ueq [Å^2] for zc-01-255b

Atom	*x*	*y*	*z*	U_{eq}
Cl1A	0.6999(5)	0.0508(6)	−0.2785(5)	0.0481(7)
O5	0.6329(14)	0.2509(13)	−0.1199(13)	0.0316(17)
C19A	0.5404(5)	0.0045(6)	−0.2349(4)	0.032(2)
Cl3B	0.7605(9)	0.0656(10)	−0.0954(6)	0.0481(7)
Cl2B	0.4885(11)	−0.1796(7)	−0.2209(9)	0.0481(7)
C18B	0.5016(14)	0.0870(11)	−0.1610(10)	0.032(2)
H18A	0.408539	0.057916	−0.218565	0.039
H18B	0.474146	0.059606	−0.098921	0.039
Br1	0.9719(3)	0.4076(3)	0.7922(2)	0.0464(4)
C23	0.9899(17)	0.4819(15)	0.6735(11)	0.033(2)
C22	1.1294(17)	0.5558(16)	0.6641(12)	0.038(2)
H22	1.215800	0.568633	0.716621	0.045
C24	0.8632(17)	0.4595(16)	0.5972(12)	0.036(2)
H24	0.767270	0.411377	0.604507	0.044
C21	1.1436(17)	0.6102(16)	0.5805(11)	0.037(2)
H21	1.240209	0.665871	0.577134	0.044
C25	0.8799(16)	0.5105(14)	0.5066(11)	0.033(2)
H25	0.793786	0.491771	0.451418	0.040
C20	1.0169(15)	0.5852(14)	0.4982(11)	0.0308(19)
C15	1.0396(14)	0.6445(12)	0.4016(8)	0.029(2)
H15	1.140804	0.727045	0.427113	0.035
C12	1.0397(9)	0.5413(9)	0.3067(8)	0.032(3)
C17	0.9303(13)	0.7096(13)	0.3751(9)	0.025(2)
C11	0.9015(11)	0.3978(10)	0.2661(9)	0.029(3)
H11C	0.813528	0.416464	0.239012	0.035
H11D	0.887679	0.356380	0.326112	0.035
C13	1.1751(11)	0.5095(13)	0.3408(9)	0.030(3)
H13C	1.169892	0.470916	0.402846	0.036
H13D	1.265998	0.601475	0.363350	0.036
C16	1.0548(19)	0.6204(15)	0.2206(11)	0.038(3)
H16D	0.974057	0.651133	0.205329	0.057
H16E	1.048970	0.555416	0.156408	0.057
H16F	1.150998	0.705223	0.244878	0.057
O3	0.9970(11)	0.8531(9)	0.4228(8)	0.0323(19)
O4	0.8037(11)	0.6496(10)	0.3200(9)	0.039(2)
C10	0.9061(13)	0.2850(13)	0.1782(11)	0.040(3)
H10C	0.817394	0.192351	0.161302	0.048
H10D	0.904606	0.318485	0.113636	0.048
C14	1.1906(12)	0.4018(11)	0.2554(10)	0.036(3)
H14A	1.209716	0.444197	0.196590	0.044
H14B	1.275754	0.380766	0.285681	0.044
C18	0.9016(15)	0.9242(14)	0.4033(11)	0.032(2)
H18E	0.799621	0.861113	0.401505	0.038
H18F	0.896886	0.946227	0.334284	0.038
C9	1.0472(10)	0.2629(12)	0.2146(10)	0.035(3)
H9	1.038640	0.217095	0.273954	0.043
C19	0.9643(16)	1.0626(15)	0.4910(12)	0.036(2)
N1	1.0599(13)	0.1617(13)	0.1288(10)	0.0333(15)
Cl1	0.9770(5)	1.0270(5)	0.6156(3)	0.0437(8)
Cl2	0.8491(5)	1.1506(5)	0.4663(4)	0.0498(9)
Cl3	1.1489(5)	1.1770(4)	0.4944(4)	0.0464(8)
C1	1.082(2)	0.1908(17)	0.0337(13)	0.045(3)

C4	1.0686(16)	0.0348(15)	0.1390(11)	0.032(2)
O1	1.088(2)	0.2965(14)	0.0070(11)	0.066(4)
C2	1.101(2)	0.0663(16)	−0.0228(13)	0.041(3)
O2	1.0552(12)	−0.0164(11)	0.2129(9)	0.0383(19)
C3	1.0938(15)	−0.0295(14)	0.0398(12)	0.032(2)
C5	1.127(2)	0.0360(17)	−0.1185(13)	0.045(4)
H5	1.137681	0.101515	−0.160886	0.054
C8	1.1111(15)	−0.1527(15)	0.0099(11)	0.033(3)
H8	1.108327	−0.215237	0.054196	0.040
C6	1.1382(17)	−0.0926(19)	−0.1508(13)	0.045(4)
H6	1.149732	−0.118066	−0.218421	0.054
C7	1.1330(15)	−0.1834(17)	−0.0881(13)	0.042(3)
H7	1.144508	−0.268950	−0.111631	0.051
C22B	0.7219(15)	0.6772(14)	−0.3053(11)	0.029(3)
H22B	0.808340	0.749101	−0.311930	0.035
Cl2A	0.3928(5)	0.0049(6)	−0.3319(5)	0.0481(7)
Cl3A	0.4889(8)	−0.1705(7)	−0.2170(7)	0.0481(7)
C18A	0.574(2)	0.1002(11)	−0.1265(7)	0.032(2)
H18C	0.480637	0.072950	−0.106413	0.039
H18D	0.645728	0.082447	−0.074800	0.039
C17A	0.5389(15)	0.3153(12)	−0.1288(19)	0.0308(16)
O4A	0.4044(13)	0.2469(16)	−0.1562(16)	0.035(2)
C15B	0.6131(13)	0.4781(10)	−0.1035(9)	0.0335(17)
H15B	0.721509	0.503836	−0.073455	0.040
H15A	0.716349	0.486770	−0.075002	0.040
C12B	0.5740(9)	0.5528(8)	−0.0143(7)	0.034(2)
C20B	0.6019(15)	0.5234(13)	−0.2051(11)	0.030(2)
C11B	0.4195(10)	0.5502(13)	−0.0486(10)	0.032(3)
H11A	0.343666	0.449085	−0.068890	0.038
H11B	0.409874	0.586766	−0.111822	0.038
C13B	0.6919(12)	0.7085(9)	0.0282(10)	0.028(3)
H13A	0.697599	0.753476	−0.030877	0.033
H13B	0.790178	0.708939	0.058001	0.033
C16B	0.5812(18)	0.4729(14)	0.0705(9)	0.037(3)
H16A	0.513619	0.370366	0.039914	0.056
H16B	0.551468	0.513705	0.128086	0.056
H16C	0.683316	0.483328	0.097982	0.056
C21B	0.7288(15)	0.6294(16)	−0.2165(11)	0.033(3)
H21B	0.820756	0.668508	−0.161776	0.039
C25B	0.4659(16)	0.4674(13)	−0.2855(11)	0.033(3)
H25B	0.378121	0.397793	−0.278623	0.039
C10B	0.3875(16)	0.6405(14)	0.0368(11)	0.034(3)
H10A	0.288665	0.639565	0.008331	0.041
H10B	0.385366	0.598070	0.097263	0.041
C26	0.6592(16)	0.7991(14)	0.1135(9)	0.029(3)
H26A	0.665134	0.761381	0.176291	0.035
H26B	0.735404	0.900219	0.135042	0.035
C24B	0.4627(16)	0.5172(13)	−0.3772(11)	0.032(3)
H24B	0.372545	0.478653	−0.433622	0.038
C9B	0.5068(16)	0.7950(14)	0.0738(10)	0.030(3)
H9B	0.504658	0.835527	0.011502	0.036
C23B	0.5890(14)	0.6206(15)	−0.3848(12)	0.030(3)
N1B	0.4809(13)	0.8888(13)	0.1561(9)	0.030(2)
Br1B	0.5791(3)	0.6852(3)	−0.5092(2)	0.0349(4)
C1C	0.4729(15)	0.8653(14)	0.2569(10)	0.031(3)

C4B	0.4840(16)	1.0223(17)	0.1527(13)	0.038(3)
O1B	0.4782(13)	0.7637(11)	0.2850(8)	0.040(2)
C2B	0.4667(15)	0.9940(14)	0.3187(10)	0.029(3)
O2B	0.5009(13)	1.0743(11)	0.0782(9)	0.040(2)
C3B	0.4737(15)	1.0900(14)	0.2555(10)	0.029(3)
C5B	0.4591(15)	1.0303(17)	0.4180(12)	0.037(3)
H5B	0.447899	0.963517	0.459351	0.044
C8B	0.4794(14)	1.2269(16)	0.2923(11)	0.035(3)
H8B	0.485806	1.292188	0.249941	0.042
C6B	0.4677(19)	1.1631(19)	0.4575(14)	0.047(4)
H6B	0.468782	1.192373	0.528347	0.056
C7B	0.4752(18)	1.2591(17)	0.3920(13)	0.042(3)
H7B	0.477234	1.350261	0.420391	0.050
C17B	0.5081(19)	0.3204(9)	−0.143(3)	0.0308(16)
O6	0.373(2)	0.254(3)	−0.173(3)	0.035(2)
Cl1B	0.6294(11)	0.0468(10)	−0.3121(5)	0.0481(7)
O3B	0.5862(11)	0.2400(13)	−0.134(2)	0.0316(17)
C19B	0.5911(7)	0.0084(7)	−0.1960(6)	0.032(2)

U_{eq} is defined as 1/3 of the trace of the orthogonalized U_{ij} tensor.

Table 3. Anisotropic displacement parameters (Å^2) for zc-01-255b. The anisotropic displacement factor exponent takes the form: $-2\pi^2[h^2(a^*)^2U_{11} + k^2(b^*)^2U_{22} + \ldots + 2hka^*b^*U_{12}]$

Atom	U_{11}	U_{22}	U_{33}	U_{23}	U_{13}	U_{12}
Cl1A	0.0578(16)	0.0397(12)	0.0509(16)	0.0134(11)	0.0222(12)	0.0216(12)
O5	0.032(2)	0.0322(19)	0.032(2)	0.0089(14)	0.0090(15)	0.0157(15)
C19A	0.038(6)	0.033(2)	0.032(5)	0.0099(18)	0.016(5)	0.018(2)
Cl3B	0.0578(16)	0.0397(12)	0.0509(16)	0.0134(11)	0.0222(12)	0.0216(12)
Cl2B	0.0578(16)	0.0397(12)	0.0509(16)	0.0134(11)	0.0222(12)	0.0216(12)
C18B	0.038(6)	0.033(2)	0.032(5)	0.0099(18)	0.016(5)	0.018(2)
Br1	0.0658(10)	0.0381(8)	0.0355(8)	0.0140(6)	0.0227(7)	0.0166(7)
C23	0.041(3)	0.027(5)	0.031(4)	0.001(3)	0.011(2)	0.016(3)
C22	0.040(3)	0.040(5)	0.030(3)	0.006(4)	0.009(3)	0.016(3)
C24	0.040(3)	0.035(5)	0.033(3)	0.004(3)	0.011(2)	0.016(3)
C21	0.037(3)	0.042(6)	0.030(3)	0.007(3)	0.009(2)	0.017(3)
C25	0.037(3)	0.030(5)	0.031(3)	0.002(3)	0.010(2)	0.016(3)
C20	0.037(3)	0.027(4)	0.028(3)	0.000(3)	0.009(2)	0.016(3)
C15	0.032(5)	0.026(5)	0.027(3)	0.000(3)	0.007(3)	0.015(4)
C12	0.044(7)	0.033(6)	0.030(6)	0.014(5)	0.020(6)	0.021(6)
C17	0.026(6)	0.031(6)	0.018(5)	0.009(4)	0.005(4)	0.013(5)
C11	0.035(6)	0.044(7)	0.016(5)	0.014(5)	0.011(5)	0.020(6)
C13	0.039(7)	0.031(6)	0.026(6)	0.009(5)	0.020(5)	0.015(5)
C16	0.058(9)	0.030(6)	0.031(7)	0.013(5)	0.016(6)	0.020(6)
O3	0.036(5)	0.024(4)	0.034(5)	0.005(4)	0.009(4)	0.013(4)
O4	0.036(5)	0.031(5)	0.046(6)	0.006(4)	0.005(4)	0.016(4)
C10	0.048(8)	0.025(6)	0.041(8)	0.014(6)	0.005(6)	0.012(6)
C14	0.042(7)	0.050(8)	0.033(7)	0.015(6)	0.016(6)	0.032(7)
C18	0.034(3)	0.033(3)	0.031(3)	0.0120(19)	0.008(2)	0.015(2)
C9	0.041(7)	0.042(7)	0.033(7)	0.008(6)	0.024(6)	0.022(6)
C19	0.037(3)	0.035(3)	0.035(3)	0.0094(19)	0.012(2)	0.013(2)
N1	0.037(3)	0.034(2)	0.031(2)	0.0122(16)	0.0129(19)	0.0148(18)

Cl1	0.059(2)	0.0506(19)	0.0215(14)	0.0092(13)	0.0131(14)	0.0237(16)
Cl2	0.062(2)	0.0390(17)	0.057(2)	0.0087(15)	0.0161(18)	0.0324(17)
Cl3	0.0388(17)	0.0305(16)	0.059(2)	0.0131(15)	0.0109(15)	0.0054(13)
C1	0.073(9)	0.035(3)	0.036(3)	0.016(3)	0.026(4)	0.025(4)
C4	0.033(6)	0.034(2)	0.034(3)	0.014(2)	0.016(3)	0.014(3)
O1	0.134(12)	0.046(4)	0.046(5)	0.026(4)	0.049(6)	0.051(6)
C2	0.061(8)	0.030(3)	0.036(3)	0.015(3)	0.025(4)	0.018(4)
O2	0.051(5)	0.039(4)	0.037(3)	0.018(3)	0.023(3)	0.023(4)
C3	0.035(6)	0.028(3)	0.036(3)	0.014(2)	0.019(3)	0.009(4)
C5	0.062(10)	0.039(8)	0.039(8)	0.021(6)	0.028(7)	0.016(7)
C8	0.029(6)	0.036(7)	0.034(7)	0.006(5)	0.010(5)	0.016(5)
C6	0.038(8)	0.054(9)	0.036(8)	0.002(7)	0.015(6)	0.014(7)
C7	0.025(6)	0.047(8)	0.048(9)	−0.003(7)	0.012(6)	0.014(6)
C22B	0.034(7)	0.032(6)	0.029(6)	0.007(5)	0.017(5)	0.017(5)
Cl2A	0.0578(16)	0.0397(12)	0.0509(16)	0.0134(11)	0.0222(12)	0.0216(12)
Cl3A	0.0578(16)	0.0397(12)	0.0509(16)	0.0134(11)	0.0222(12)	0.0216(12)
C18A	0.038(6)	0.033(2)	0.032(5)	0.0099(18)	0.016(5)	0.018(2)
C17A	0.0318(19)	0.0307(18)	0.031(2)	0.0081(13)	0.0086(14)	0.0147(13)
O4A	0.0319(19)	0.031(3)	0.042(4)	0.009(3)	0.0087(16)	0.0148(15)
C15B	0.036(3)	0.0307(18)	0.034(4)	0.0079(16)	0.012(3)	0.0145(15)
C12B	0.040(6)	0.027(4)	0.036(4)	0.009(3)	0.015(4)	0.013(4)
C20B	0.036(6)	0.023(4)	0.032(3)	0.004(3)	0.011(3)	0.013(4)
C11B	0.029(6)	0.028(6)	0.030(7)	0.003(5)	0.005(5)	0.008(5)
C13B	0.028(6)	0.022(6)	0.030(7)	0.004(5)	0.011(5)	0.006(5)
C16B	0.061(9)	0.040(7)	0.026(6)	0.021(6)	0.020(6)	0.029(7)
C21B	0.022(5)	0.043(7)	0.037(7)	0.009(6)	0.006(5)	0.020(5)
C25B	0.035(7)	0.019(5)	0.034(7)	0.001(5)	0.007(5)	0.007(5)
C10B	0.042(7)	0.028(6)	0.028(7)	0.008(5)	0.012(5)	0.009(6)
C26	0.050(8)	0.035(7)	0.015(6)	0.005(5)	0.014(5)	0.029(6)
C24B	0.040(7)	0.021(5)	0.030(7)	0.003(5)	0.010(5)	0.009(5)
C9B	0.042(7)	0.030(6)	0.013(5)	0.010(5)	0.006(5)	0.009(5)
C23B	0.023(5)	0.032(6)	0.039(7)	0.013(5)	0.016(5)	0.012(5)
N1B	0.034(5)	0.040(6)	0.022(5)	0.014(4)	0.009(4)	0.018(5)
Br1B	0.0369(7)	0.0375(7)	0.0277(7)	0.0113(5)	0.0123(5)	0.0106(5)
C1C	0.037(7)	0.030(6)	0.018(6)	0.005(5)	0.009(5)	0.007(5)
C4B	0.036(7)	0.048(8)	0.039(8)	0.017(6)	0.023(6)	0.020(6)
O1B	0.056(6)	0.036(5)	0.035(5)	0.020(4)	0.022(5)	0.019(5)
C2B	0.033(6)	0.033(6)	0.023(6)	0.012(5)	0.011(5)	0.013(5)
O2B	0.057(6)	0.044(5)	0.042(6)	0.023(5)	0.029(5)	0.034(5)
C3B	0.038(7)	0.029(6)	0.024(6)	0.008(5)	0.006(5)	0.021(5)
C5B	0.029(6)	0.042(7)	0.033(7)	−0.001(6)	0.011(5)	0.013(6)
C8B	0.024(6)	0.044(7)	0.033(7)	0.004(6)	0.007(5)	0.012(6)
C6B	0.051(9)	0.051(9)	0.042(9)	0.002(7)	0.016(7)	0.029(7)
C7B	0.046(8)	0.045(8)	0.038(8)	0.002(6)	0.010(6)	0.028(7)
C17B	0.0318(19)	0.0307(18)	0.031(2)	0.0081(13)	0.0086(14)	0.0147(13)
O6	0.0319(19)	0.031(3)	0.042(4)	0.009(3)	0.0087(16)	0.0148(15)
Cl1B	0.0578(16)	0.0397(12)	0.0509(16)	0.0134(11)	0.0222(12)	0.0216(12)
O3B	0.032(2)	0.0322(19)	0.032(2)	0.0089(14)	0.0090(15)	0.0157(15)

C19B	0.038(6)	0.033(2)	0.032(5)	0.0099(18)	0.016(5)	0.018(2)

Table 4. Bond lengths and angles for zc-01-255b

Atom–Atom	Length [Å]
Cl1A–C19A	1.7665(19)
O5–C18A	1.430(3)
O5–C17A	1.351(3)
C19A–Cl2A	1.7666(19)
C19A–Cl3A	1.7666(19)
C19A–C18A	1.514(3)
Cl3B–C19B	1.765(3)
Cl2B–C19B	1.766(3)
C18B–H18A	0.9900
C18B–H18B	0.9900
C18B–O3B	1.430(3)
C18B–C19B	1.5243(11)
Br1–C23	1.904(15)
C23–C22	1.38(2)
C23–C24	1.38(2)
C22–H22	0.9500
C22–C21	1.35(2)
C24–H24	0.9500
C24–C25	1.43(2)
C21–H21	0.9500
C21–C20	1.41(2)
C25–H25	0.9500
C25–C20	1.36(2)
C20–C15	1.557(18)
C15–H15	1.0000
C15–C12	1.526(3)
C15–C17	1.512(16)
C12–C11	1.5251(13)
C12–C13	1.5251(13)
C12–C16	1.532(17)
C17–O3	1.353(15)
C17–O4	1.195(16)
C11–H11C	0.9900
C11–H11D	0.9900
C11–C10	1.543(12)
C13–H13C	0.9900
C13–H13D	0.9900
C13–C14	1.542(12)
C16–H16D	0.9800
C16–H16E	0.9800
C16–H16F	0.9800
O3–C18	1.428(16)
C10–H10C	0.9900
C10–H10D	0.9900
C10–C9	1.5250(13)
C14–H14A	0.9900
C14–H14B	0.9900
C14–C9	1.5248(13)
C18–H18E	0.9900
C18–H18F	0.9900
C18–C19	1.515(19)
C9–H9	1.0000
C9–N1	1.490(16)
C19–Cl1	1.768(15)
C19–Cl2	1.745(15)

C19–Cl3	1.791(14)
N1–C1	1.41(2)
N1–C4	1.380(17)
C1–O1	1.196(19)
C1–C2	1.48(2)
C4–O2	1.215(17)
C4–C3	1.509(19)
C2–C3	1.401(19)
C2–C5	1.38(2)
C3–C8	1.36(2)
C5–H5	0.9500
C5–C6	1.39(2)
C8–H8	0.9500
C8–C7	1.39(2)
C6–H6	0.9500
C6–C7	1.36(2)
C7–H7	0.9500
C22B–H22B	0.9500
C22B–C21B	1.37(2)
C22B–C23B	1.375(19)
C18A–H18C	0.9900
C18A–H18D	0.9900
C17A–O4A	1.215(15)
C17A–C15B	1.512(9)
C15B–H15B	1.0000
C15B–H15A	1.0000
C15B–C12B	1.526(3)
C15B–C20B	1.516(18)
C15B–C17B	1.504(9)
C12B–C11B	1.5253(13)
C12B–C13B	1.5251(13)
C12B–C16B	1.5253(13)
C20B–C21B	1.407(19)
C20B–C25B	1.402(19)
C11B–H11A	0.9900
C11B–H11B	0.9900
C11B–C10B	1.529(18)
C13B–H13A	0.9900
C13B–H13B	0.9900
C13B–C26	1.534(15)
C16B–H16A	0.9800
C16B–H16B	0.9800
C16B–H16C	0.9800
C21B–H21B	0.9500
C25B–H25B	0.9500
C25B–C24B	1.42(2)
C10B–H10A	0.9900
C10B–H10B	0.9900
C10B–C9B	1.521(18)
C26–H26A	0.9900
C26–H26B	0.9900
C26–C9B	1.507(19)
C24B–H24B	0.9500
C24B–C23B	1.368(18)
C9B–H9B	1.0000
C9B–N1B	1.484(18)
C23B–Br1B	1.906(14)
N1B–C1C	1.435(16)
N1B–C4B	1.383(19)
C1C–O1B	1.194(17)

C1C–C2B	1.482(19)
C4B–O2B	1.232(18)
C4B–C3B	1.490(19)
C2B–C3B	1.408(17)
C2B–C5B	1.356(19)
C3B–C8B	1.400(19)
C5B–H5B	0.9500
C5B–C6B	1.36(2)
C8B–H8B	0.9500
C8B–C7B	1.34(2)
C6B–H6B	0.9500
C6B–C7B	1.43(2)
C7B–H7B	0.9500
C17B–O6	1.219(18)
C17B–O3B	1.352(3)
Cl1B–C19B	1.765(3)

**Atom–Atom–Atom**	**Angle [°]**
C17A–O5–C18A	118.5(12)
Cl1A–C19A–Cl2A	109.01(17)
Cl1A–C19A–Cl3A	109.06(17)
Cl3A–C19A–Cl2A	109.10(17)
C18A–C19A–Cl1A	111.6(8)
C18A–C19A–Cl2A	113.7(9)
C18A–C19A–Cl3A	104.2(6)
H18A–C18B–H18B	108.1
O3B–C18B–H18A	109.5
O3B–C18B–H18B	109.5
O3B–C18B–C19B	110.7(10)
C19B–C18B–H18A	109.5
C19B–C18B–H18B	109.5
C22–C23–Br1	119.8(11)
C22–C23–C24	120.7(14)
C24–C23–Br1	119.5(11)
C23–C22–H22	119.8
C21–C22–C23	120.3(14)
C21–C22–H22	119.8
C23–C24–H24	120.9
C23–C24–C25	118.2(14)
C25–C24–H24	120.9
C22–C21–H21	119.5
C22–C21–C20	120.9(14)
C20–C21–H21	119.5
C24–C25–H25	119.5
C20–C25–C24	121.1(13)
C20–C25–H25	119.5
C21–C20–C15	118.9(12)
C25–C20–C21	118.6(13)
C25–C20–C15	122.5(12)
C20–C15–H15	105.9
C12–C15–C20	116.6(10)
C12–C15–H15	105.9
C17–C15–C20	107.7(9)
C17–C15–H15	105.9
C17–C15–C12	113.9(9)
C15–C12–C16	106.9(9)
C11–C12–C15	113.6(9)
C11–C12–C16	110.9(10)
C13–C12–C15	107.6(8)
C13–C12–C11	107.8(9)

C13–C12–C16	109.9(9)
O3–C17–C15	109.8(10)
O4–C17–C15	127.8(11)
O4–C17–O3	122.5(11)
C12–C11–H11C	108.6
C12–C11–H11D	108.6
C12–C11–C10	114.8(9)
H11C–C11–H11D	107.6
C10–C11–H11C	108.6
C10–C11–H11D	108.6
C12–C13–H13C	108.5
C12–C13–H13D	108.5
C12–C13–C14	115.0(9)
H13C–C13–H13D	107.5
C14–C13–H13C	108.5
C14–C13–H13D	108.5
C12–C16–H16D	109.5
C12–C16–H16E	109.5
C12–C16–H16F	109.5
H16D–C16–H16E	109.5
H16D–C16–H16F	109.5
H16E–C16–H16F	109.5
C17–O3–C18	113.9(10)
C11–C10–H10C	109.7
C11–C10–H10D	109.7
H10C–C10–H10D	108.2
C9–C10–C11	109.8(10)
C9–C10–H10C	109.7
C9–C10–H10D	109.7
C13–C14–H14A	109.8
C13–C14–H14B	109.8
H14A–C14–H14B	108.3
C9–C14–C13	109.2(9)
C9–C14–H14A	109.8
C9–C14–H14B	109.8
O3–C18–H18E	110.1
O3–C18–H18F	110.1
O3–C18–C19	108.2(11)
H18E–C18–H18F	108.4
C19–C18–H18E	110.1
C19–C18–H18F	110.1
C10–C9–H9	107.4
C14–C9–C10	114.3(10)
C14–C9–H9	107.4
N1–C9–C10	111.0(10)
N1–C9–C14	109.0(9)
N1–C9–H9	107.4
C18–C19–Cl1	111.1(10)
C18–C19–Cl2	108.2(10)
C18–C19–Cl3	110.2(10)
Cl1–C19–Cl3	107.0(8)
Cl2–C19–Cl1	110.9(8)
Cl2–C19–Cl3	109.4(8)
C1–N1–C9	124.7(11)
C4–N1–C9	123.0(11)
C4–N1–C1	111.9(12)
N1–C1–C2	105.8(12)
O1–C1–N1	126.1(15)
O1–C1–C2	128.1(15)
N1–C4–C3	106.9(11)

O2–C4–N1	126.3(13)
O2–C4–C3	126.8(12)
C3–C2–C1	108.9(13)
C5–C2–C1	131.9(14)
C5–C2–C3	119.1(14)
C2–C3–C4	106.5(12)
C8–C3–C4	130.9(12)
C8–C3–C2	122.5(13)
C2–C5–H5	120.9
C6–C5–C2	118.2(14)
C6–C5–H5	120.9
C3–C8–H8	121.3
C3–C8–C7	117.4(14)
C7–C8–H8	121.3
C5–C6–H6	119.2
C7–C6–C5	121.6(14)
C7–C6–H6	119.2
C8–C7–H7	119.5
C6–C7–C8	121.0(14)
C6–C7–H7	119.5
C21B–C22B–H22B	120.2
C23B–C22B–H22B	120.2
C23B–C22B–C21B	119.6(12)
O5–C18A–C19A	113.8(10)
O5–C18A–H18C	108.8
O5–C18A–H18D	108.8
C19A–C18A–H18C	108.8
C19A–C18A–H18D	108.8
H18C–C18A–H18D	107.7
O5–C17A–C15B	115.2(11)
O4A–C17A–O5	122.3(11)
O4A–C17A–C15B	122.6(11)
C17A–C15B–H15B	104.3
C17A–C15B–C12B	113.1(11)
C17A–C15B–C20B	109.6(12)
C12B–C15B–H15B	104.3
C12B–C15B–H15A	108.3
C20B–C15B–H15B	104.3
C20B–C15B–H15A	108.3
C20B–C15B–C12B	119.6(9)
C17B–C15B–H15A	108.3
C17B–C15B–C12B	110.3(17)
C17B–C15B–C20B	101.5(17)
C11B–C12B–C15B	113.4(9)
C11B–C12B–C16B	109.4(9)
C13B–C12B–C15B	107.5(7)
C13B–C12B–C11B	109.8(8)
C13B–C12B–C16B	110.2(9)
C16B–C12B–C15B	106.4(8)
C21B–C20B–C15B	119.3(12)
C21B–C20B–C25B	119.3(12)
C25B–C20B–C15B	121.4(11)
C12B–C11B–H11A	108.9
C12B–C11B–H11B	108.9
C12B–C11B–C10B	113.5(10)
H11A–C11B–H11B	107.7
C10B–C11B–H11A	108.9
C10B–C11B–H11B	108.9
C12B–C13B–H13A	109.0
C12B–C13B–H13B	109.0

C12B–C13B–C26	112.8(9)
H13A–C13B–H13B	107.8
C26–C13B–H13A	109.0
C26–C13B–H13B	109.0
C12B–C16B–H16A	109.5
C12B–C16B–H16B	109.5
C12B–C16B–H16C	109.5
H16A–C16B–H16B	109.5
H16A–C16B–H16C	109.5
H16B–C16B–H16C	109.5
C22B–C21B–C20B	121.0(13)
C22B–C21B–H21B	119.5
C20B–C21B–H21B	119.5
C20B–C25B–H25B	120.8
C20B–C25B–C24B	118.4(12)
C24B–C25B–H25B	120.8
C11B–C10B–H10A	109.6
C11B–C10B–H10B	109.6
H10A–C10B–H10B	108.1
C9B–C10B–C11B	110.2(11)
C9B–C10B–H10A	109.6
C9B–C10B–H10B	109.6
C13B–C26–H26A	109.4
C13B–C26–H26B	109.4
H26A–C26–H26B	108.0
C9B–C26–C13B	111.1(11)
C9B–C26–H26A	109.4
C9B–C26–H26B	109.4
C25B–C24B–H24B	119.9
C23B–C24B–C25B	120.2(13)
C23B–C24B–H24B	119.9
C10B–C9B–H9B	107.7
C26–C9B–C10B	110.7(11)
C26–C9B–H9B	107.7
N1B–C9B–C10B	112.8(11)
N1B–C9B–C26	109.9(10)
N1B–C9B–H9B	107.7
C22B–C23B–C24B	121.4(13)
C22B–C23B–Br1B	119.8(9)
C24B–C23B–Br1B	118.8(10)
C1C–N1B–C9B	124.9(11)
C4B–N1B–C9B	124.2(10)
C4B–N1B–C1C	110.2(11)
N1B–C1C–C2B	106.7(11)
O1B–C1C–N1B	125.2(12)
O1B–C1C–C2B	128.0(12)
N1B–C4B–C3B	107.7(11)
O2B–C4B–N1B	123.9(13)
O2B–C4B–C3B	128.3(13)
C3B–C2B–C1C	107.6(11)
C5B–C2B–C1C	132.3(12)
C5B–C2B–C3B	120.1(13)
C2B–C3B–C4B	107.8(11)
C8B–C3B–C4B	129.5(13)
C8B–C3B–C2B	122.6(12)
C2B–C5B–H5B	120.4
C2B–C5B–C6B	119.1(15)
C6B–C5B–H5B	120.4
C3B–C8B–H8B	122.5
C7B–C8B–C3B	115.0(14)

C7B–C8B–H8B	122.5
C5B–C6B–H6B	120.1
C5B–C6B–C7B	119.8(15)
C7B–C6B–H6B	120.1
C8B–C7B–C6B	123.3(14)
C8B–C7B–H7B	118.3
C6B–C7B–H7B	118.3
O6–C17B–C15B	134.1(19)
O6–C17B–O3B	115.9(15)
O3B–C17B–C15B	109.8(12)
C17B–O3B–C18B	116.7(3)
Cl3B–C19B–Cl2B	109.1(2)
Cl3B–C19B–Cl1B	109.3(2)
C18B–C19B–Cl3B	109.6(6)
C18B–C19B–Cl2B	109.77(18)
C18B–C19B–Cl1B	109.84(18)
Cl1B–C19B–Cl2B	109.2(2)

Table 5. Torsion angles for zc-01-255b

Atom–Atom–Atom–Atom	Torsion Angle [°]
Cl1A–C19A–C18A–O5	56.4(16)
O5–C17A–C15B–C12B	−121.6(18)
O5–C17A–C15B–C20B	102.2(19)
Br1–C23–C22–C21	−179.4(11)
Br1–C23–C24–C25	−177.1(9)
C23–C22–C21–C20	−4(2)
C23–C24–C25–C20	−3.2(19)
C22–C23–C24–C25	2.1(19)
C22–C21–C20–C25	3(2)
C22–C21–C20–C15	−177.4(12)
C24–C23–C22–C21	1(2)
C24–C25–C20–C21	0.9(19)
C24–C25–C20–C15	−179.1(11)
C21–C20–C15–C12	93.7(14)
C21–C20–C15–C17	−136.8(12)
C25–C20–C15–C12	−86.4(14)
C25–C20–C15–C17	43.1(14)
C20–C15–C12–C11	54.3(13)
C20–C15–C12–C13	−65.0(13)
C20–C15–C12–C16	176.9(11)
C20–C15–C17–O3	94.6(11)
C20–C15–C17–O4	−86.3(15)
C15–C12–C11–C10	−173.6(9)
C15–C12–C13–C14	177.9(10)
C15–C17–O3–C18	−179.3(10)
C12–C15–C17–O3	−134.5(10)
C12–C15–C17–O4	44.7(18)
C12–C11–C10–C9	53.9(14)
C12–C13–C14–C9	−54.6(14)
C17–C15–C12–C11	−72.2(13)
C17–C15–C12–C13	168.5(10)
C17–C15–C12–C16	50.5(13)
C17–O3–C18–C19	157.6(10)
C11–C12–C13–C14	55.0(12)
C11–C10–C9–C14	−52.4(15)
C11–C10–C9–N1	−176.2(10)
C13–C12–C11–C10	−54.4(12)
C13–C14–C9–C10	52.5(15)
C13–C14–C9–N1	177.4(10)
C16–C12–C11–C10	66.1(12)
C16–C12–C13–C14	−66.0(12)
O3–C18–C19–Cl1	−58.8(13)
O3–C18–C19–Cl2	179.3(9)
O3–C18–C19–Cl3	59.7(12)
O4–C17–O3–C18	1.5(17)
C10–C9–N1–C1	66.4(17)
C10–C9–N1–C4	−121.4(14)
C14–C9–N1–C1	−60.4(17)
C14–C9–N1–C4	111.8(14)
C9–N1–C1–O1	−3(3)
C9–N1–C1–C2	175.3(12)
C9–N1–C4–O2	6(2)
C9–N1–C4–C3	−175.1(10)
N1–C1–C2–C3	−2(2)
N1–C1–C2–C5	−179.0(19)
N1–C4–C3–C2	0.9(16)
N1–C4–C3–C8	179.6(14)
C1–N1–C4–O2	179.4(15)
C1–N1–C4–C3	−2.1(17)
C1–C2–C3–C4	0.6(18)
C1–C2–C3–C8	−178.3(14)
C1–C2–C5–C6	−179.6(18)
C4–N1–C1–O1	−176.1(19)
C4–N1–C1–C2	2.4(19)
C4–C3–C8–C7	−179.9(14)
O1–C1–C2–C3	177(2)
O1–C1–C2–C5	−1(4)
C2–C3–C8–C7	−1(2)
C2–C5–C6–C7	−4(3)
O2–C4–C3–C2	179.4(15)
O2–C4–C3–C8	−2(3)
C3–C2–C5–C6	3(3)
C3–C8–C7–C6	1(2)
C5–C2–C3–C4	178.2(15)
C5–C2–C3–C8	−1(3)
C5–C6–C7–C8	2(2)
Cl2A–C19A–C18A–O5	−67.4(15)
Cl3A–C19A–C18A–O5	174.0(12)

C18A–O5–C17A–O4A	−10(3)
C18A–O5–C17A–C15B	170.5(14)
C17A–O5–C18A–C19A	97(2)
C17A–C15B–C12B–C11B	−77.4(13)
C17A–C15B–C12B–C13B	161.0(11)
C17A–C15B–C12B–C16B	42.9(14)
C17A–C15B–C20B–C21B	−134.2(12)
C17A–C15B–C20B–C25B	49.3(15)
O4A–C17A–C15B–C12B	59(3)
O4A–C17A–C15B–C20B	−77(3)
C15B–C12B–C11B–C10B	−172.9(9)
C15B–C12B–C13B–C26	175.8(10)
C15B–C20B–C21B–C22B	−177.5(11)
C15B–C20B–C25B–C24B	178.4(11)
C15B–C17B–O3B–C18B	175(2)
C12B–C15B–C20B–C21B	92.9(14)
C12B–C15B–C20B–C25B	−83.6(13)
C12B–C15B–C17B–O6	52(6)
C12B–C15B–C17B–O3B	−122(3)
C12B–C11B–C10B–C9B	55.7(14)
C12B–C13B–C26–C9B	−55.6(14)
C20B–C15B–C12B–C11B	54.0(12)
C20B–C15B–C12B–C13B	−67.6(12)
C20B–C15B–C12B–C16B	174.4(11)
C20B–C15B–C17B–O6	−76(5)
C20B–C15B–C17B–O3B	110(3)
C20B–C25B–C24B–C23B	−1.9(18)
C11B–C12B–C13B–C26	52.1(13)
C11B–C10B–C9B–C26	−57.0(13)
C11B–C10B–C9B–N1B	179.3(10)
C13B–C12B–C11B–C10B	−52.7(13)
C13B–C26–C9B–C10B	57.3(13)
C13B–C26–C9B–N1B	−177.4(10)
C16B–C12B–C11B–C10B	68.4(12)
C16B–C12B–C13B–C26	−68.6(13)
C21B–C22B–C23B–C24B	0(2)
C21B–C22B–C23B–Br1B	−179.3(10)
C21B–C20B–C25B–C24B	1.9(18)
C25B–C20B–C21B–C22B	−0.8(19)
C25B–C24B–C23B–C22B	1(2)
C25B–C24B–C23B–Br1B	−179.6(10)
C10B–C9B–N1B–C1C	59.9(16)
C10B–C9B–N1B–C4B	−130.5(13)
C26–C9B–N1B–C1C	−64.2(16)
C26–C9B–N1B–C4B	105.4(14)
C9B–N1B–C1C–O1B	−4(2)
C9B–N1B–C1C–C2B	172.4(11)
C9B–N1B–C4B–O2B	4(2)
C9B–N1B–C4B–C3B	−172.4(12)
C23B–C22B–C21B–C20B	−0.2(19)
N1B–C1C–C2B–C3B	−1.0(14)
N1B–C1C–C2B–C5B	−179.6(14)
N1B–C4B–C3B–C2B	0.8(15)
N1B–C4B–C3B–C8B	176.7(13)
C1C–N1B–C4B–O2B	174.9(14)
C1C–N1B–C4B–C3B	−1.5(15)
C1C–C2B–C3B–C4B	0.1(15)
C1C–C2B–C3B–C8B	−176.1(12)
C1C–C2B–C5B–C6B	174.4(15)
C4B–N1B–C1C–O1B	−174.9(14)
C4B–N1B–C1C–C2B	1.5(14)
C4B–C3B–C8B–C7B	−176.4(14)
O1B–C1C–C2B–C3B	175.4(14)
O1B–C1C–C2B–C5B	−3(3)
C2B–C3B–C8B–C7B	−1(2)
C2B–C5B–C6B–C7B	4(2)
O2B–C4B–C3B–C2B	−175.4(14)
O2B–C4B–C3B–C8B	0(3)
C3B–C2B–C5B–C6B	−4(2)
C3B–C8B–C7B–C6B	1(2)
C5B–C2B–C3B–C4B	178.9(12)
C5B–C2B–C3B–C8B	3(2)
C5B–C6B–C7B–C8B	−2(2)
C17B–C15B–C12B–C11B	−63.1(16)
C17B–C15B–C12B–C13B	175.4(14)
C17B–C15B–C12B–C16B	57.3(16)
C17B–C15B–C20B–C21B	−145.6(14)
C17B–C15B–C20B–C25B	37.9(16)
O6–C17B–O3B–C18B	0(5)
O3B–C18B–C19B–Cl3B	56.8(15)
O3B–C18B–C19B–Cl2B	176.6(14)
O3B–C18B–C19B–Cl1B	−63.3(15)
C19B–C18B–O3B–C17B	154(3)

Bibliography

[1] Crysalispro, *1.171.39.20a*, **2015**, Rigaku OD.
[2] Crysalispro, *1.171.39.20a*, **2015**, Rigaku OD.
[3] G. M. Sheldrick, *Acta Cryst.* **2015**, *A71*, 3–8, doi:10.1107/S2053273314026370.
[4] G. M. Sheldrick, *Acta Cryst.* **2015**, *C71*, 3–8, doi:10.1107/S2053229614024218.
[5] C. R. Groom, I. J. Bruno, M. P. Lightfoot, S. C. Ward, *Acta Cryst.* **2016**, *B72*, 171–179, doi:10.1107/S2052520616003954.
[6] D. Kratzert, *FinalCif*, *V113*, https://dkratzert.de/finalcif.html.
